# A global analysis of COVID-19 infection fatality rate and its associated factors during the Delta and Omicron variant periods: an ecological study

**DOI:** 10.3389/fpubh.2023.1145138

**Published:** 2023-06-02

**Authors:** Nhi Thi Hong Nguyen, Tsong-Yih Ou, Le Duc Huy, Chung-Liang Shih, Yao-Mao Chang, Thanh-Phuc Phan, Chung-Chien Huang

**Affiliations:** ^1^School of Health Care Administration, College of Management, Taipei Medical University, Taipei, Taiwan; ^2^Health Personnel Training Institute, University of Medicine and Pharmacy, Hue University, Hue, Vietnam; ^3^Division of Infectious Diseases, Department of Internal Medicine, Taipei Municipal Wanfang Hospital-Managed by Taipei Medical University, Taipei, Taiwan; ^4^Department of Nursing, Cardinal Tien Junior College of Healthcare and Management, Taipei, Taiwan; ^5^Department of Medical Quality, Taipei Municipal Wanfang Hospital-Managed by Taipei Medical University, Taipei, Taiwan; ^6^National Health Insurance Administration, Ministry of Health and Welfare, Taipei, Taiwan; ^7^Taiwan Centers for Disease Control, Taipei, Taiwan; ^8^International Ph.D. Program in Biotech and Healthcare Management, College of Management, Taipei Medical University, Taipei, Taiwan; ^9^University of Medical Center, Ho Chi Minh City, Vietnam; ^10^Department of Long-Term Care, School of Gerontology Health Management, College of Nursing, Taipei Medical University, Taipei, Taiwan; ^11^Department and School of Pharmacy, College of Pharmacy, Taipei Medical University, Taipei, Taiwan

**Keywords:** COVID-19, variants, Delta, Omicron, vaccine, NPIs

## Abstract

**Background:**

The Omicron variant of SARS-CoV-2 is more highly infectious and transmissible than prior variants of concern. It was unclear which factors might have contributed to the alteration of COVID-19 cases and deaths during the Delta and Omicron variant periods. This study aimed to compare the COVID-19 average weekly infection fatality rate (AWIFR), investigate factors associated with COVID-19 AWIFR, and explore the factors linked to the increase in COVID-19 AWIFR between two periods of Delta and Omicron variants.

**Materials and methods:**

An ecological study has been conducted among 110 countries over the first 12 weeks during two periods of Delta and Omicron variant dominance using open publicly available datasets. Our analysis included 102 countries in the Delta period and 107 countries in the Omicron period. Linear mixed-effects models and linear regression models were used to explore factors associated with the variation of AWIFR over Delta and Omicron periods.

**Findings:**

During the Delta period, the lower AWIFR was witnessed in countries with better government effectiveness index [β = −0.762, 95% CI (−1.238)–(−0.287)] and higher proportion of the people fully vaccinated [β = −0.385, 95% CI (−0.629)–(−0.141)]. In contrast, a higher burden of cardiovascular diseases was positively associated with AWIFR (β = 0.517, 95% CI 0.102–0.932). Over the Omicron period, while years lived with disability (YLD) caused by metabolism disorders (β = 0.843, 95% CI 0.486–1.2), the proportion of the population aged older than 65 years (β = 0.737, 95% CI 0.237–1.238) was positively associated with poorer AWIFR, and the high proportion of the population vaccinated with a booster dose [β = −0.321, 95% CI (−0.624)–(−0.018)] was linked with the better outcome. Over two periods of Delta and Omicron, the increase in government effectiveness index was associated with a decrease in AWIFR [β = −0.438, 95% CI (−0.750)–(−0.126)]; whereas, higher death rates caused by diabetes and kidney (β = 0.472, 95% CI 0.089–0.855) and percentage of population aged older than 65 years (β = 0.407, 95% CI 0.013–0.802) were associated with a significant increase in AWIFR.

**Conclusion:**

The COVID-19 infection fatality rates were strongly linked with the coverage of vaccination rate, effectiveness of government, and health burden related to chronic diseases. Therefore, proper policies for the improvement of vaccination coverage and support of vulnerable groups could substantially mitigate the burden of COVID-19.

## Introduction

The COVID-19 pandemic has led to significant mortality and morbidity worldwide, with over 661 million infected people and 6.7 million deaths mid-January 2023 (1, 2). In the early stages of the pandemic, governments implemented non-pharmaceutical interventions (NPIs) to contain the virus and later launched vaccination campaigns to achieve herd immunity (3, 4). However, the emergence of new variants resulted in new pandemic waves in many countries, even with high vaccination coverage rates (5, 6). The novel variants, especially Delta and Omicron, exhibited faster transmission and reduced vaccine efficacy, leading to increases in infection, hospitalization, and mortality rates, as well as substantial burden on socioeconomic and healthcare systems (7, 8). The preliminary findings indicated that the Omicron variant was 100 times more transmissible than the Delta variant (9–12). Therefore, it is essential for governments to keep track of country-level pandemic indicators and traverse appropriate COVID-19 control remedies effectively and rapidly.

Although the infection fatality rate of COVID-19 has been investigated as a key metric to evaluate the effectiveness of NPIs in previous studies, there is limited knowledge about the changes in infection fatality rate between different COVID-19 variants (13). In addition, few studies were implemented at the global level to identify the possible factors associated with COVID-19 average weekly infection fatality rate (AWIFR). A comprehensive understanding of how factors such as socioeconomic characteristics, government performance, healthcare capacity, comorbidities, and environmental factors (e.g., PM 2.5) influence the AWIFR would contribute to fostering effective policies and improving success in controlling the COVID-19 pandemic (14, 15). Moreover, the understanding of key factors could provide valuable information to cope with the potential outbreaks related to respiratory viruses with different mutations in future. To address these research gaps, our study aims to compare AWIFR and investigate factors associated with COVID-19 AWIFR over each of the two periods of Delta and Omicron variants. Additionally, we explored the factors contributing to the increase in COVID-19 AWIFR between these two periods.

## Materials and methods

### Study design

An ecological study was conducted among 110 countries during the Delta (102 countries) and Omicron (107 countries) variant dominance periods.

### Inclusion criteria

We selected countries providing the available data on COVID-19 outcomes (i.e., total cases and total deaths) from Our World^®^ in Data (16), the prevalence of variant of concern on GISAID^®^ databases, and vaccination data from the global vaccination database (17).

### Study period

The period of variant dominance is recorded as the week when the variant prevalence is over 50% (18) and followed up for 12 weeks. We selected 12 weeks because it is the maximum day we can collect the data across 102 countries in the Delta variant period and 107 countries in the Omicron variant period as of the data collection date (14 April 2022).

**Figure 1:**
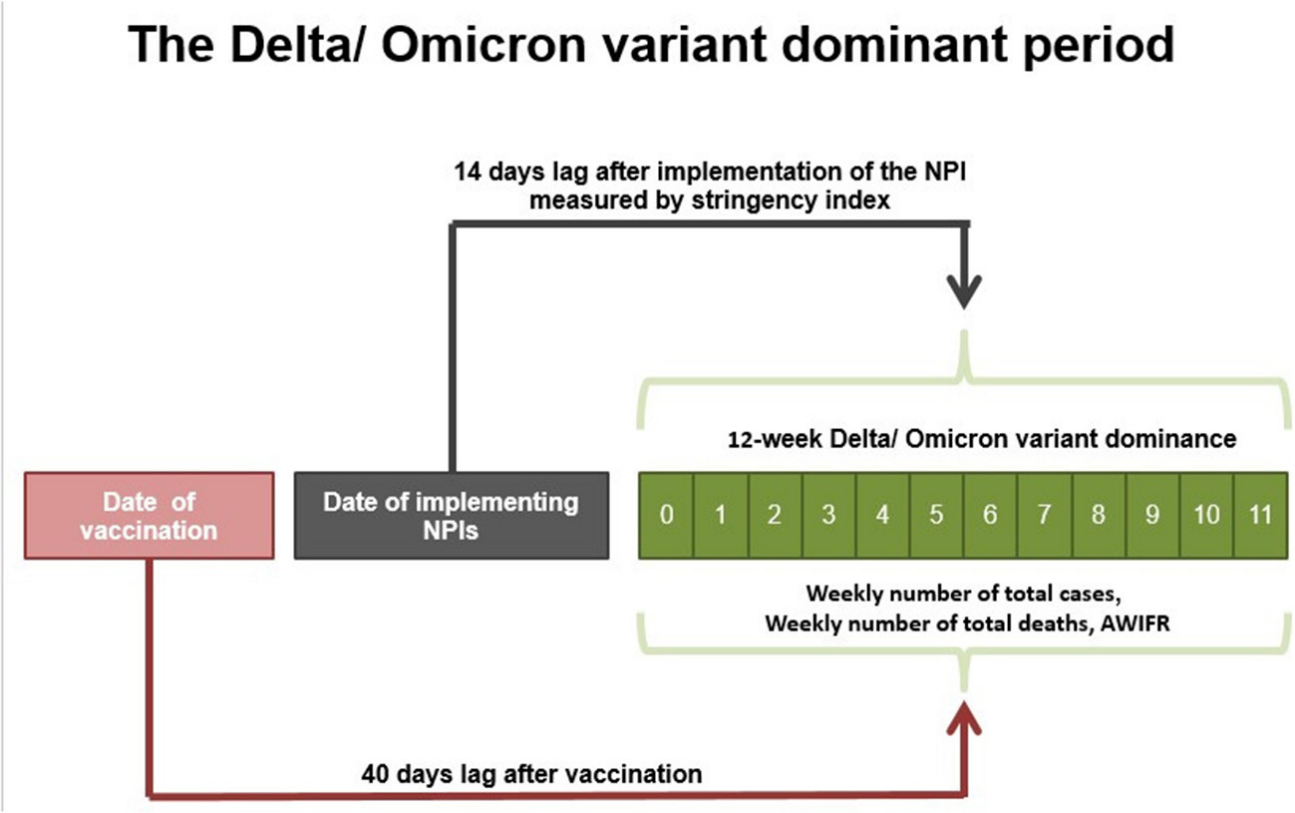
Time lag effects were identified throughout the period of variant dominance.

Previous research found that NPIs (defined by the stringency index) and vaccines influence the pandemic after at least 14 and 40 days, respectively. As a result, we constructed a 14-day lagged stringency index and a 40-day lagged vaccination in our research (19). Figure 1 presents the 12-week period of Delta and Omicron variant dominance.

### Outcomes

Our interest outcome is AWIFR of the specific country. COVID-19 AWIFR was calculated as the average weekly number of total COVID-19 deaths divided by the average weekly number of total COVID-19 infections multiplied by 100 (2, 13).


$$\begin{array}{l} AWIFR_{j}\;=\;\overset{n}{\underset{j=1}{\sum }}\frac{F_{j}}{I_{j}}x\;100\;with\;n\;countries;j=1,2,\ldots n \end{array}$$

where

*F*_*j*_ = The weekly number of total deaths due to COVID-19 per 100,000 inhabitants in a particular country over a given period.

*I*_*j*_ = The weekly number of total infections due to COVID-19 per 100,000 inhabitants in a particular country over a given period.

As for the countries presenting in both Delta and Omicron periods (99 countries), we calculated the increase in AWIFR over two periods.

### Data collection

Our analysis was aggregated and extracted from different open publicly available databases. Our World^®^ in Data was used to identify the total cases and total deaths (16). GISAID (Global Initiative on Sharing Avian Influenza Data) was used to identify variants of concerns (VOCs) and estimate the first date of Delta and Omicron appeared in a country (20). The global vaccine database was used to retrieve the proportion of the population vaccinated with at least one dose, the proportion of the population fully vaccinated, and the proportion of the population vaccinated with the booster dose (21). The Oxford COVID-19 Government Response Tracker was used to extract the stringency index (22). The World Bank Open Data were used to retrieve data on country-level indices, demographic and socioeconomic features (total population (N), population density (people per square kilometer), urban population (% population living in urban area), the proportion of the population older than 65 years of age, the GDP per capita, median age) (13). The Worldwide Governance Indicators website was used to retrieve the government effectiveness index, government rule index, and government quality index (23). The 2019 Global Health Security Index database was used to retrieve six categories as follows: prevention of pathogen release (GHSI1), detection and reporting for epidemics (GHSI2), rapid response to an epidemic (GHSI3), capability of the health system to treat patients and protect healthcare workers (GHSI4), compliance with international commitments (GHSI5), and nationwide environmental risk and public health vulnerability to biological threats (GHSI6) (24). The Global Burden of Disease database was used to retrieve the death rate of chronic diseases per 100,000 inhabitants (i.e., cancer, cardiovascular diseases, chronic kidney disease, and diabetes), the number of years lived with disability (YLDs) of nutrition deficiency (iron deficiency, vitamin A deficiency, etc.), health behaviors (i.e., metabolic risks, tobacco), and environment risks (e.g., PM2.5) (25). The World Bank Open Data and World Health Organization Data were used to retrieve healthcare capacity [universal health coverage (UHC) index, healthcare access, and quality (HAQ) index, health expenditure (% of GDP), the number of hospital beds, physicians, and nurses per 1,000 people] (23, 26).

### Independent variables

Sociodemographic variables included total population, population density (people per square kilometer), urban population (% population lived in urban area), the proportion of the population older than 65 years of age, the GDP per capita (current US$), and median age (13). Government performance indicators included the government effectiveness index, government rule index, and government quality index (23). COVID-19 prevention measures included two indicators, namely, stringency index and vaccine coverage. The stringency index measures the government's response to containment and closure policies (22). Vaccine data were extracted from the global vaccine database, comprising the proportion of the population vaccinated with at least one dose, the proportion of the population fully vaccinated, and the proportion of the population vaccinated with the booster dose (21). Global health security (GHS) index was a comprehensive assessment of the health security capability of a country to prevent and combat epidemic. The index had an overall score and comprised six categories as follows: prevention of pathogen release (GHSI1), detection and reporting for epidemics (GHSI2), rapid response to an epidemic (GHSI3), capability of the health system to treat patients and protect healthcare workers (GHSI4), compliance with international commitments (GHSI5), and nationwide environmental risk and public health vulnerability to biological threats (GHSI6) (24). Healthcare capacity included the UHC index, health expenditure (% of GDP), the number of hospital beds, physicians, and nurses per 1,000 people, and HAQ index. The HAQ index analyzed the 32 causes of death that are considered avoidable in the availability of quality medical services. Causes of death included various health service areas, such as vaccine-preventable diseases, epidemics and maternal and child health, non-infectious diseases, and gastrointestinal diseases in which death is preventable by surgery (23, 26). Health characteristics among the population included the death rate of chronic diseases per 100,000 inhabitants (i.e., cancer, cardiovascular diseases, chronic kidney disease, and diabetes), the number of YLDs of nutrition deficiency (iron deficiency, vitamin A deficiency, etc.), health behaviors (i.e., metabolic risks, tobacco), and environment risks (e.g., PM2.5) (25).

Supplementary Table 1 in the Multimedia Appendix 1 defines all variables and data sources.

### Analysis

- Summary data extracted from the 12-week Delta and Omicron periods.

We computed the outcome (AWIFR) and aggregated vaccination rates and average stringency index over 12 weeks in each period. The independent variables were categorized into three equal groups (Tertile 1—low level, Tertile 2—medium level, and Tertile 3—high level). Categorical variables were described using frequencies and percentages. The associations between categorical variables were measured using the chi-square test. Continuous variables were presented with mean and standard deviation or median and interquartile range. The Shapiro–Wilk test was used to test the normal distribution of variables, and the Mann–Whitney *U*-test or Kruskal–Wallis test was used for the continuous variable. Linear regression models were utilized to determine the associations of independent variables with the increase in AWIFR, average weekly infection rate, and average weekly fatality rate between two periods of the Delta and Omicron variants.

The independent variables (sociodemographic characteristics, government performance indicators, global health security index, healthcare capacity, HAQ index, death rate of chronic diseases per 100,000 inhabitants, YLDs per 100,000 inhabitants, health behaviors, and environment risks) that showed the significance in the univariate analysis were selected and sorted based on *R*^2^ in decreasing order. Then, we systematically entered these variables and eliminated those with insignificant statistics in the multivariate analysis. We utilized the forward selection method based on *R*^2^ to choose the best model. *P* < 0.05 indicate the significance. For longitudinal data of AWIFR during Delta and Omicron periods, linear mixed-effects models were employed to explore factors associated with the variation of AWIFR over Delta and Omicron periods. We performed the univariate analysis of linear mixed-effects models for each covariate including stringency index, percentage of the population fully vaccinated/received booster doses, and independent variables (sociodemographic characteristics, government performance indicators, global health security index, healthcare capacity, HAQ index, death rate of chronic diseases per 100,000 inhabitants, YLDs per 100,000 inhabitants, health behaviors, and environment risks). The covariates with a *p*-value of < 0.05 in the univariate analyses were selected and ranked in decreasing order according to the goodness of fit of the univariate model, as defined by the Bayesian Information Criterion (BIC). We fitted a series of multivariable linear mixed-effects models with time and imputed each covariate sequentially based on its ranks. If an independent variable was an insignificant predictor, it was dropped from the forward selection models. Since the time series data of AWIFR were highly skewed, we implemented a log-transformation of outcome. The random intercepts and slope models were used to analyze the time-varying characteristics of the selected countries. The detailed steps of model selection are described in the Multimedia Appendix 2.

- Multicollinearity: We checked the multi-collinearity between the independent variables using pairwise scatterplot and correlation coefficients. If two or more variables moderately or strongly correlated with each other, one representative variable was kept in the multivariate model.
- Normality and homogeneity of variance tests: As the increase in AWIFR had a skewed distribution, the log-transformation of the outcome variable was applied before carrying the linear regression model to determine the associations of independent variables with the increase in AWIFR between two periods of the Delta and Omicron variants. For longitudinal data, since the data of AWIFR were highly skewed, we implemented a log-transformation of outcome. We used Levene's test to test for homogeneity of variance.

All statistical analyses and visualization in the study were conducted using an R programming language (version 4.1.3). The LME4 package was adopted to build the linear mixed-effects model (27).

## Results

### General characteristics of selected countries

Table 1 presents the demographics of 110 countries. The median population was 11,167,545.5, and the median population density was 84.30. Regarding government performance, the median of the rule of law, regulatory quality, and government effectiveness indexes were −0.12, 0.06, and 0.04, respectively. Regarding healthcare capacity, the median of the UHC and overall GHS indices were 71.5 and 41.9, respectively. The HAQ score was 20.64. In terms of chronic diseases, cardiovascular diseases accounted for the highest death rate per 100,000 inhabitants with 235.59. For environmental and health behavior factors, metabolism disorders caused the highest YLDs per 100,000 inhabitants with 1,099.93. Zinc deficiency, in contrast, was observed to have the lowest death rate with 0.117.

**Table 1:** The demographics of selected countries (*N* = 110).

**Characteristic**	**Mean (SD)**	**Median (Q1, Q3)^a^**
**Socioeconomic characteristics**		
Population (N)	34,565,721.03 (58,019,686.72)	11,167,545.5 (5,018,753, 36,348,444)
Population density (P/km^2)^	278.09 (1,007.96)	84.30 (36.62, 205.50)
Life expectancy	74.02 (7.55)	75.45 (70.89, 79.98)
GDP per capita (USD per capita)	21,628.00 (21,152.44)	15,062.93 (6,184.55, 31,089.42)
% population aged over 65	9.95 (6.41)	8.08 (3.96, 16.10)
% Urban population	63.87 (21.42)	67.12 (50.81, 80.54)
Median age (years of age)	32.18 (9.23)	31.9 (25.05, 41.2)
Gender (% of total population)		
Male	49.88 (3.34)	49.60 (48.93, 50.14)
Female	50.12 (3.34)	50.40 (49.87, 51.07)
**Government performance**		
Rule of law	0.09 (0.99)	−0.12 (−0.59, 0.92)
Regulatory quality	0.18 (0.96)	0.06 (−0.53, 0.91)
Government effectiveness	0.14 (0.97)	0.04 (−0.56, 0.94)
**Health care capacity**		
UHC index	67.24 (14.49)	71.5 (61, 77)
Overall GHS index	43.63 (13.61)	41.9 (32.5, 54.63)
GHSI1: prevention	34.10 (17.70)	34.7 (19.28, 47.1)
GHSI2: early detection and reporting	39.62 (18.40)	37.85 (28.3, 52.58)
GHSI3: rapid response	40.82 (12.49)	40.7 (30.93, 49.65)
GHSI4: health system	37.62 (18.55)	39.7 (20.33, 52.85)
GHSI5: compliance	51.62 (13.06)	51.65 (42.33, 61.5)
GHSI6: risk environment	58.23 (15.22)	57.45 (47.58, 69.4)
HAQ index	26.68 (23.07)	20.64 (16.83, 28.94)
No. Physicians per 1,000	2.09 (1.61)	2.07 (0.71, 3.11)
No. Nurse and midwife per 1,000	5.07 (4.57)	3.57 (1.40, 7.36)
% GDP for health expenditure	6.62 (2.55)	6.71 (4.64, 7.84)
Hospital beds (per 1,000 people)	2.92 (2.09)	2.45 (1.4, 3.58)
**Heath burden of chronic diseases (death rate per 100,000)**		
Non-communicable diseases	565.28 (177.31)	543.53 (413.99, 657.48)
Diabetes	31.51 (28.06)	21.11 (11.46, 41.36)
Chronic respiratory disease	35.79 (31.81)	26.93 (20.74, 40.59)
Cancers	127.31 (28.27)	124.17 (111.82, 142.62)
Chronic kidney diseases	24.35 (14.84)	22.40 (11.13, 31.87)
Cardiovascular diseases	251.94 (122.28)	235.59 (157.08, 312.40)
Diabetes and kidney diseases	55.92 (39.08)	43.73 (21.90, 75.46)
**Heath burden of environmental and health behavior risk (years lived with disability (YLDs) per 100,000)**		
PM2.5	214.47 (139.07)	181.17 (97.43, 320.58)
Tobacco	428.48 (175.87)	454.29 (273.28, 538.85)
Zinc deficiency	0.16 (0.13)	0.12 (0.05, 0.19)
Vitamin A deficiency	11.57 (16.75)	4.87 (0.47, 11.95)
Low bone density	96.09 (41.77)	80.73 (65.91, 128.67)
Metabolism disorders	1,189.94 (278.95)	1,099.93 (983.29, 1,365.08)

a 25th percentile and 75th percentile.

### Stringency index and vaccine characteristics between two periods

Table 2 shows the stringency of NPIs and level of vaccine coverage among 102 countries across the Delta variant period and 107 countries across the Omicron variant period. The median stringency index in the Delta period was 52.52 points, higher than that of the Omicron period by 6.35 points (*P* < 0.001). Regarding vaccine coverage, the proportions of the population vaccinated at least one dose and fully vaccinated during the Omicron period were 65.47 and 61.03%, respectively, much higher than that of the Delta period, with 49.14 and 34.92% (*P* < 0.001, *P* < 0.001), respectively.

**Table 2:** Vaccine and stringency index characteristics between two periods.

**Characteristic**	**Delta (*N* = 102)**		**Omicron (*N* = 107)**		***P*** **-value** ^*^
	**Mean (SD)**	**Median (Q1, Q3)**	**Mean (SD)**	**Median (Q1, Q3)**	
Stringency index	51.91 (15.07)	52.52 (39.73, 63.89)	46.17 (14.48)	46.69 (35.16, 56.48)	0.006
% population vaccinated at least one dose	43.67 (27.05)	49.14 (18.63, 68.39)	56.72 (28.35)	65.47 (32.28, 79.95)	< 0.001
% population fully vaccinated	35.49 (5.53)	34.92 (12.46, 59.99)	53.12 (26.99)	61.03 (31.59, 75.79)	< 0.0001
% population vaccinated with a booster dose	1.49 (5.61)	0 (0, 0.13)	22.98 (22.59)	16.04 (0.35, 42.38)	N/A^a^

a N/A, not applicable.

* P-values based on the Mann–Whitney U-test for the differences of vaccine and stringency index between two periods.

**Figure 2:**
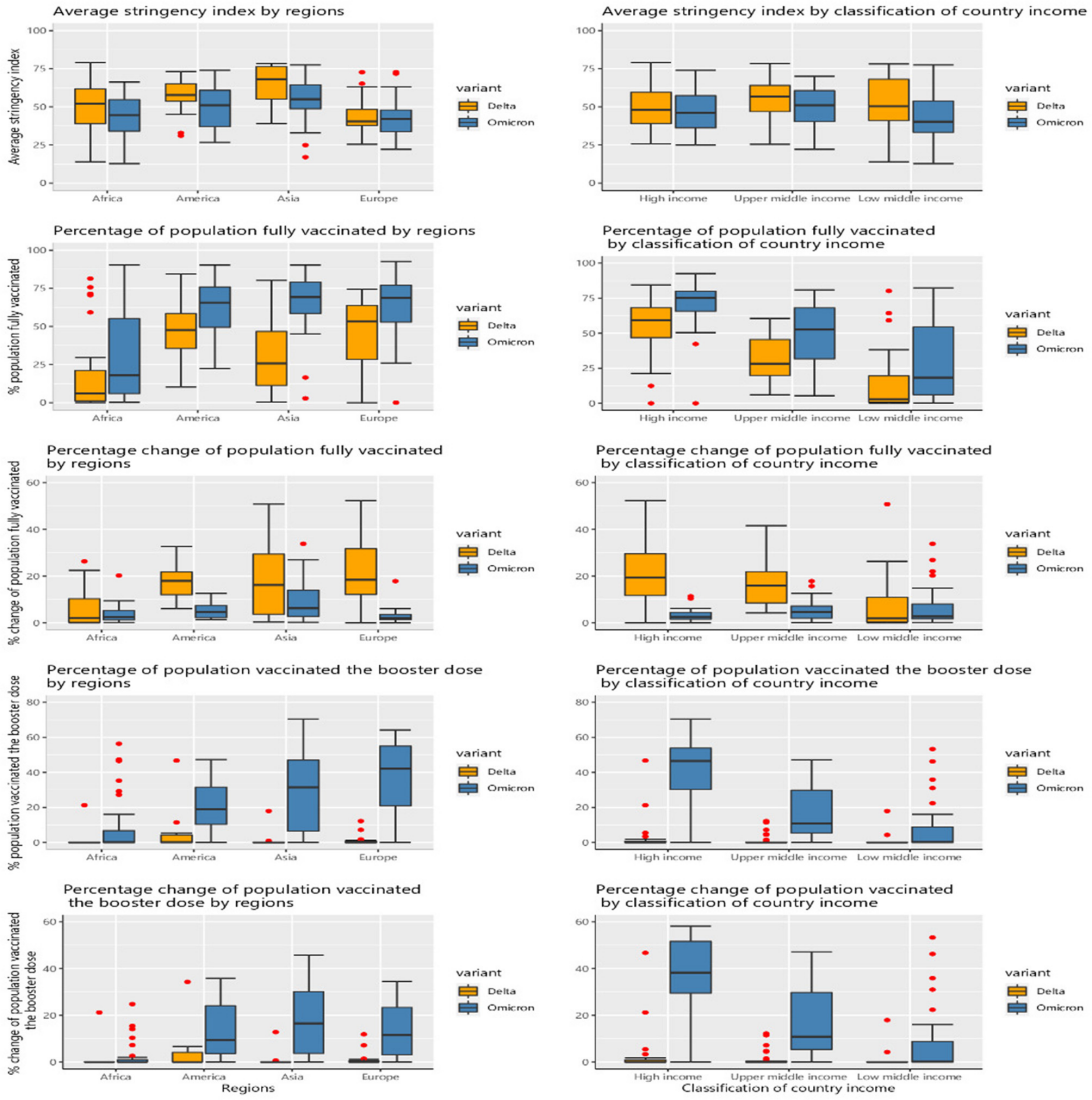
Vaccine and stringency index characteristics by regions and income groups between two periods in each period of the Delta and Omicron variants.

Figure 2 presents the stringency of NPIs and level of vaccine coverage by regions and country income among 102 countries across the Delta variant period and 107 countries across the Omicron variant period. South-East Asia had the highest stringency index in both of the two periods, with a median of 68.07 points and 55.03 points (*P* = 0.02), respectively. Regarding vaccine coverage, during the Delta period, the European region accounted for the highest percentages in terms of the people fully vaccinated (*P* < 0.001), people vaccinated with a booster dose, and change of population fully vaccinated (*P* < 0.001) with 53.2, 42.15, and 18.46%, respectively. The proportion of the population fully vaccinated in high-income countries was 59.29%, while the figures for the low-income countries were meager with 2.93% (*P* < 0.001). During the Omicron period, most regions having the proportion of the population fully vaccinated were over 65%, except for Africa (17.92%). Europe obtained the highest proportion of the population vaccinated with a booster dose with 42.15%. High-income countries reached the highest proportions regarding fully vaccinated (*P* < 0.001), vaccinated with a booster dose, and change of population vaccinated with the booster dose with 75.21, 46.93, and 17.57%, respectively.

### COVID-19 health-related outcomes of selected countries

Table 3 provides COVID-19 health-related outcomes. The median AWIFR during the Delta period was 0.09 (Q1–Q3, 0.05–0.19), higher than that of the Omicron period (*P* < 0.001) with 0.03 (Q1–Q3, 0.02–0.07). The median value of the average weekly infection rate during the Omicron period was 287.43 (Q1–Q3, 43.71–929), higher than 4.4 times that of the Delta period (*P* = 0.003).

**Table 3:** COVID-19 health-related outcomes of selected countries.

**Characteristics**	**Delta (*****N*** = **102)**		**Omicron (*****N*** = **107)**		***P*** **-value** ^*^
	**Mean (SD)**	**Median (Q1, Q3)**	**Mean (SD)**	**Median (Q1, Q3)**	
COVID-19 crude mortality rate	15.30 (20.09)	7.10 (2.41, 20.17)	17.28 (17.53)	14.11 (2.19, 27.30)	0.228
COVID-19 average weekly infection rate	105.17 (146.48)	65.40 (16.03, 141.41)	552.71 (665.19)	287.43 (43.71, 929)	0.003
COVID-19 average weekly fatality rate	1.28 (1.67)	0.59 (0.20, 1.68)	1.44 (1.46)	1.17 (0.18, 2.27)	0.794
COVID-19 AWIFR	0.14 (0.16)	0.09 (0.05, 0.19)	0.04 (0.04)	0.03 (0.02, 0.07)	< 0.001

* P-values based on the Mann–Whitney U-test for the differences of COVID-19 crude mortality rate, COVID-19 average weekly infection rate, COVID-19 average weekly fatality rate, and COVID-19 AWIFR.

**Figure 3:**
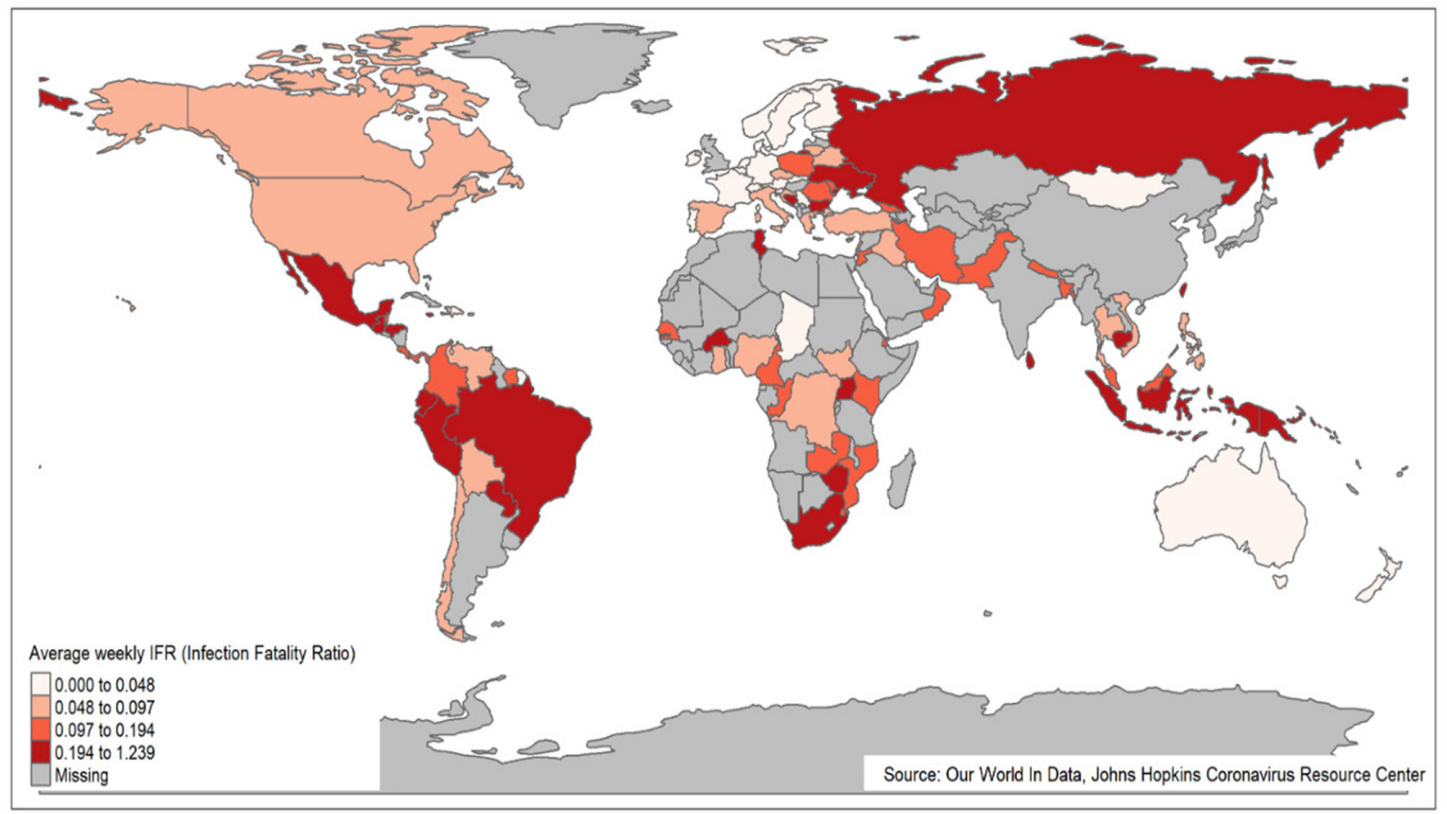
World map of AWIFR among countries during the Delta variant period.

Figure 3 presents a global picture of AWIFR among countries during the Delta period. The countries with high AWIFR were mainly located in South American countries (e.g., Paraguay, Peru, and Brazil) and East and Southeast Asia (e.g., Indonesia, Cambodia, and Taiwan). Meanwhile, countries witnessing low AWIFR were Chad, Qatar, New Zealand, Norway, and the Netherlands.

**Figure 4:**
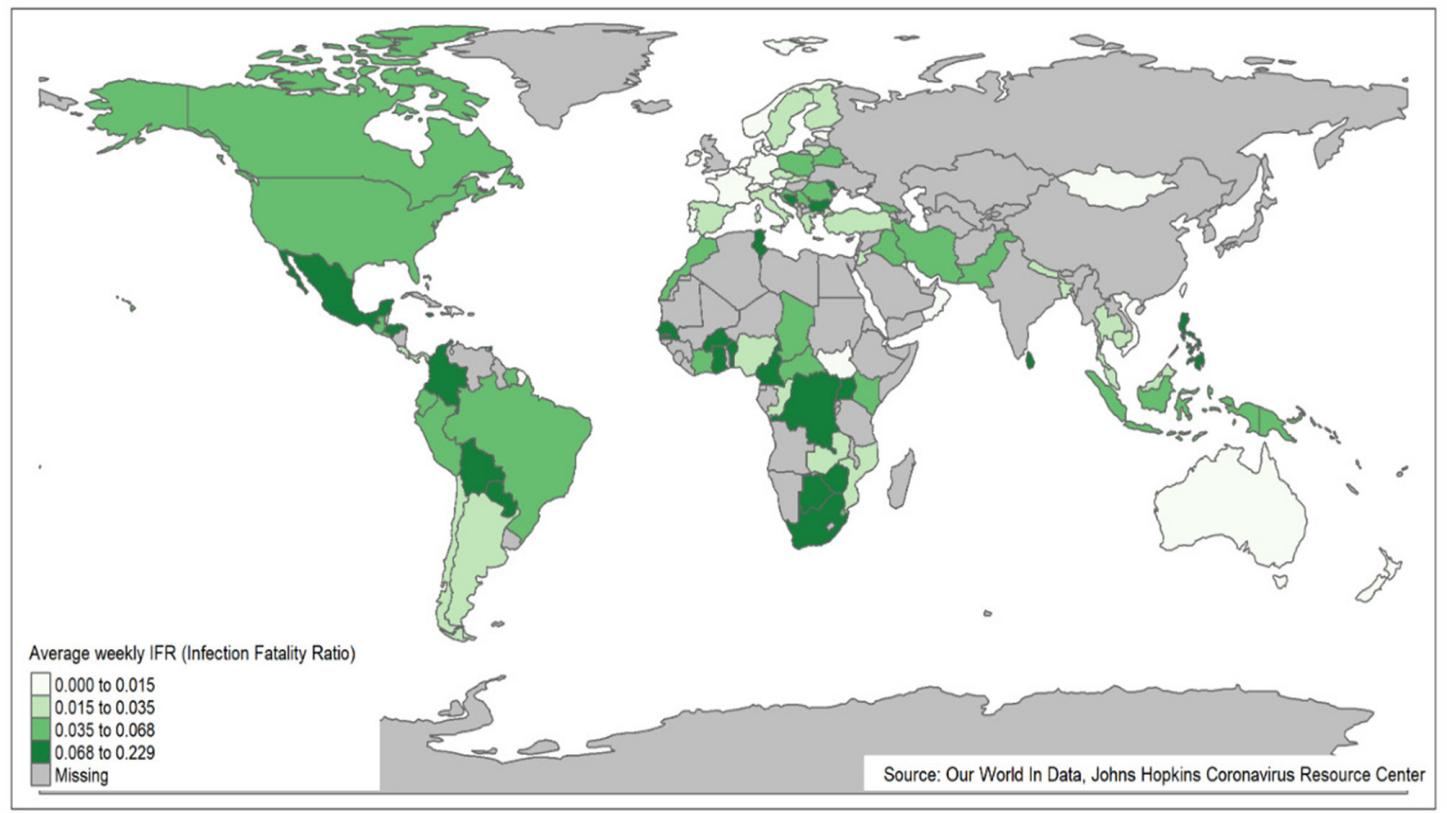
World map of AWIFR among countries during the Omicron variant period.

Figure 4 presents the world map of AWIFR among countries during the Omicron variant period. The countries, namely, Bosnia and Herzegovina, Sri Lanka, and Burkina Faso, observed high AWIFR. The Netherlands, New Zealand, Mongolia, and Kuwait countries, in contrast, witnessed low AWIFR.

**Figure 5:**
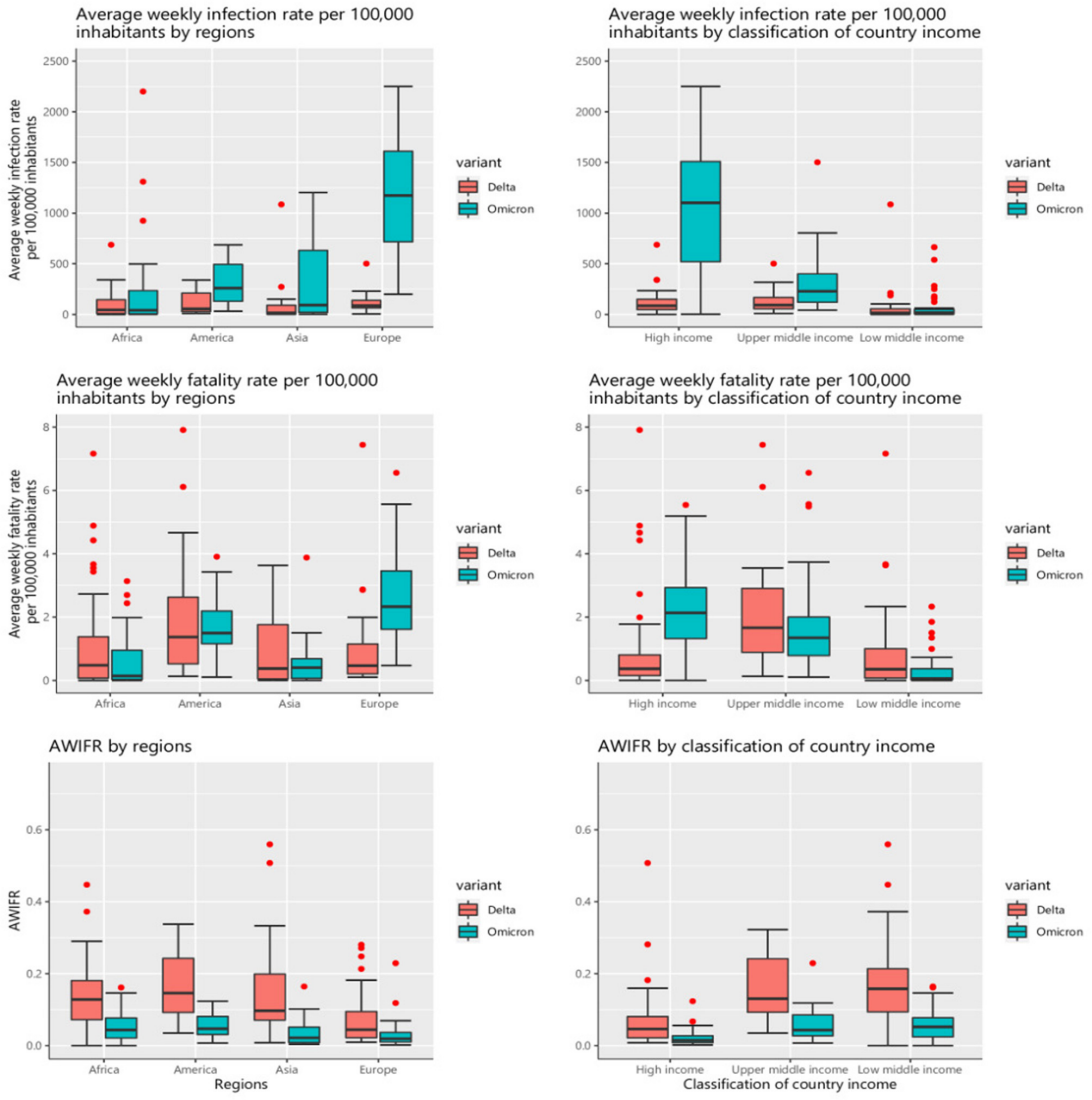
COVID-19 outcomes by regions and country income classification between two periods of the Delta and Omicron variants.

Figure 5 shows the COVID-19 outcomes by region and country income classification between two periods of the Delta and Omicron variants. The highest rates of average weekly infection (*P* < 0.001) and average weekly fatality (*P* = 0.01) were observed in Europe during the Omicron variant period, with 1,201.21 (Q1–Q3, 745.60–1,711.48) and 2.33 (Q1–Q3, 1.61–3.46), respectively. Similarly, high-income countries were found to have the highest rates of average weekly infection (*P* = 0.006) and average weekly fatality (*P* < 0.001) during the Omicron, with 1,101.99 (Q1–Q3, 561.01–1,525.25) and 2.13 (Q1–Q3, 1.32–2.93), respectively. During both two periods of the Delta and Omicron, the highest AWIFR was found in the Americas [*P* < 0.001; with a median of 0.16 (Q1–Q3, 0.09–0.27) and 0.05 (Q1–Q3, 0.03–0.08), respectively] and low-income countries [*P* < 0.001; with a median of 0.16 (Q1–Q3, 0.09–0.21) and 0.05 (Q1–Q3, 0.02–0.08), respectively].

### The factors associated with AWIFR during the Delta and Omicron periods

**Figure 6:**
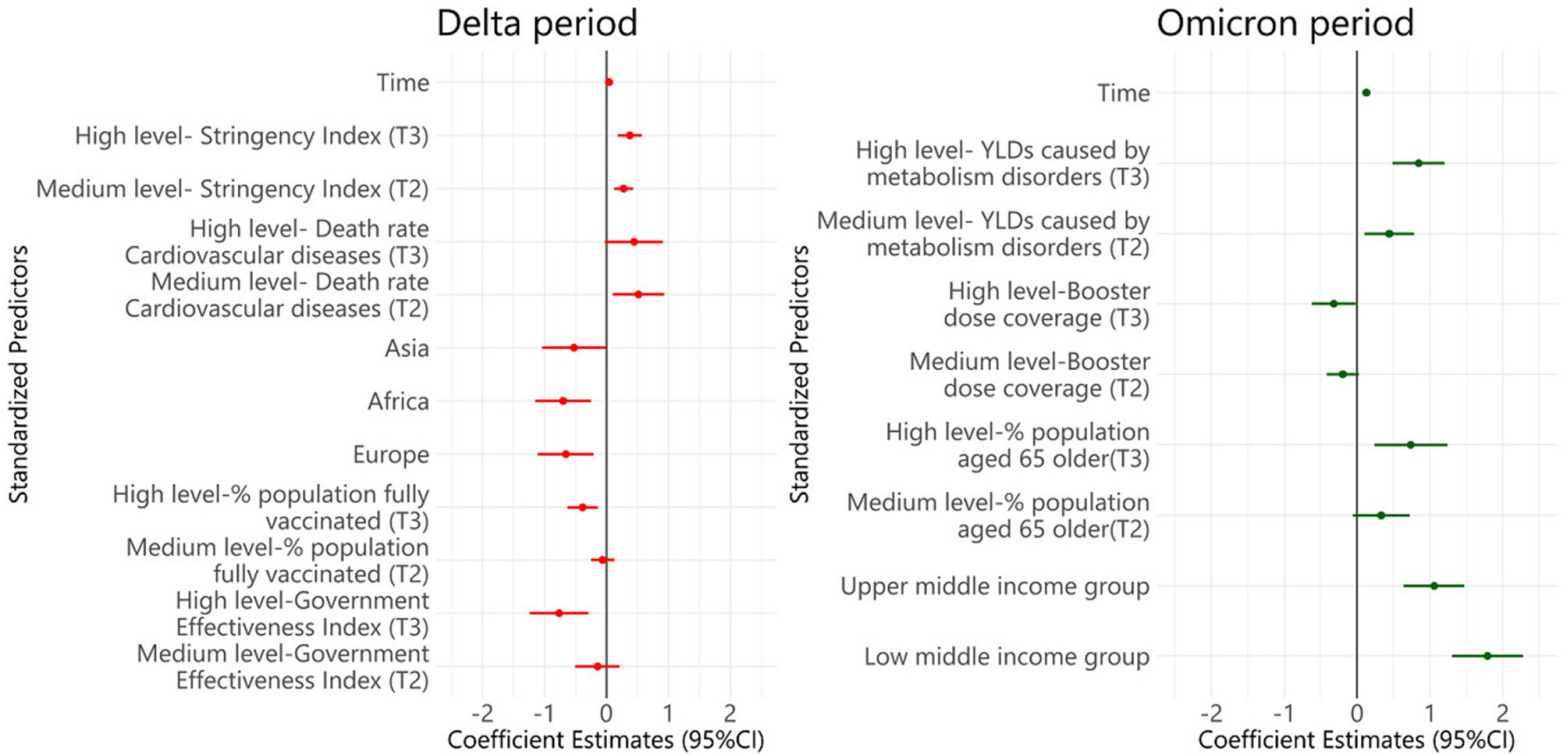
Multivariable linear mixed-effects models of factors associated with the AWIFR during the Delta and Omicron periods. Tertile 1, Low level; Tertile 2, Medium level; Tertile 3, High level; YLDs, Years lived with disability.

Figure 6 presents the findings of the multivariable linear mixed-effects models to investigate the factors associated with the AWIFR in each period. During the period of the Delta variant dominance, the death rate caused by cardiovascular diseases and intensity of the stringency index was positively associated with the log-transformation of AWIFR (β = 0.517, 95% CI 0.102–0.932). Meanwhile, the government effectiveness index [β = −0.762, 95% CI (−1.238)–(−0.287)], world regions [β = −0.655, 95% CI (−1.103)–(−0.208)], and the proportion of the people fully vaccinated [β = −0.385, 95% CI (−0.629)–(−0.141)] had negative associations with the outcome. Regarding the Omicron variant dominance period, YLDs caused by metabolism disorders, income level, and the proportion of the population aged older than 65 years were found to be positively associated with log-transformation of AWIFR (β = 0.843, 95% CI 0.486–1.2; β = 1.788, 95% CI 1.301–2.276; β = 0.737, 95% CI 0.237–1.238, respectively). A lower log-transformation of AWIFR was observed in countries with a high proportion of the population vaccinated with a booster dose [β = −0.321, 95% CI (−0.624)–(−0.018)].

Adjusted for life expectancy, the population aged older than 65 years, hospital beds per 1,000 inhabitants, number of nurses per 1,000 inhabitants, health expenditure, death rate of diabetes, death rate of chronic kidney diseases, death rate of cancer, death rate of chronic respiratory diseases, YLDs caused by low bone density, YLDs caused by iron deficiency.

### The increase of stringency index, vaccination, and AWIFR by regions and classification of country income among 99 countries between two periods of the Delta and Omicron variants

**Figure 7:**
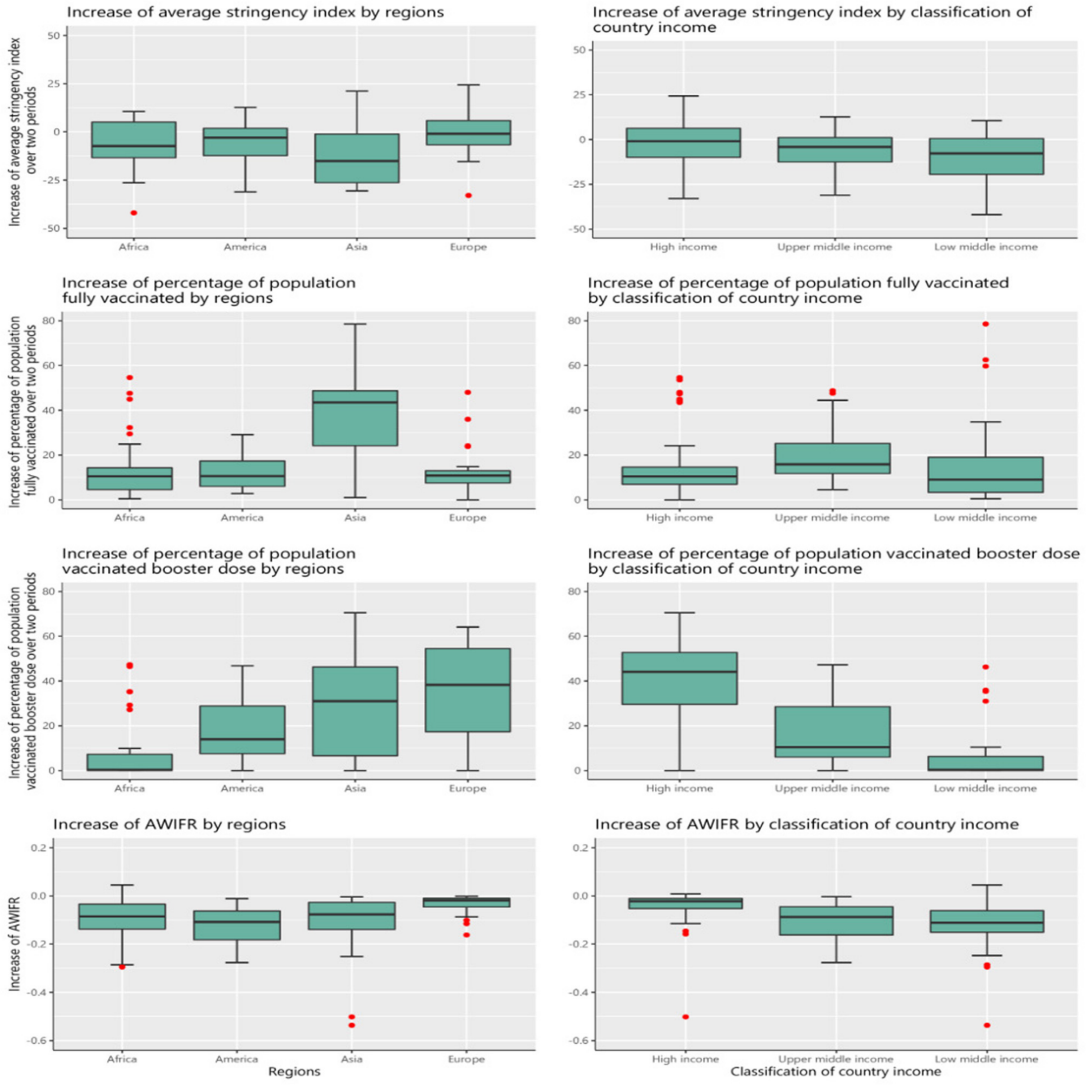
The increase in stringency index, vaccination, and AWIFR by regions and classification of country income between two periods of the Delta and Omicron.

Figure 7 presents the increase in stringency index, percentage of the population fully vaccinated, vaccinated booster dose, and AWIFR by region and country income classification. Over two periods, Asia saw the most significant decline in the average stringency index and the greatest gain in the proportion of full vaccination. Between the Delta and Omicron periods, Africa had the lowest increase (10%) in the proportion of the population that had received a booster dose and the proportion of the population that had been vaccinated. In contrast, European nations had the most significant population growth vaccinated with booster doses. We discovered that the AWIFR dropped in most regions, with the most significant decline reported in the Americas during the two periods (*P* < 0.05). Comparing the index among groups of nations by income during two periods, we observed that the proportion of the fully vaccinated population grew significantly in upper-middle-income and low-income countries (*P* < 0.05). However, the proportion of the population inoculated with the booster dose increased the most in the high-income nations (over 40%). In addition, the AWIFR was shown to fall the least in high-income countries (*P* < 0.05).

### The regression model of factors associated with the increase in AWIFR among 99 countries between two periods of the Delta and Omicron variants

**Figure 8:**
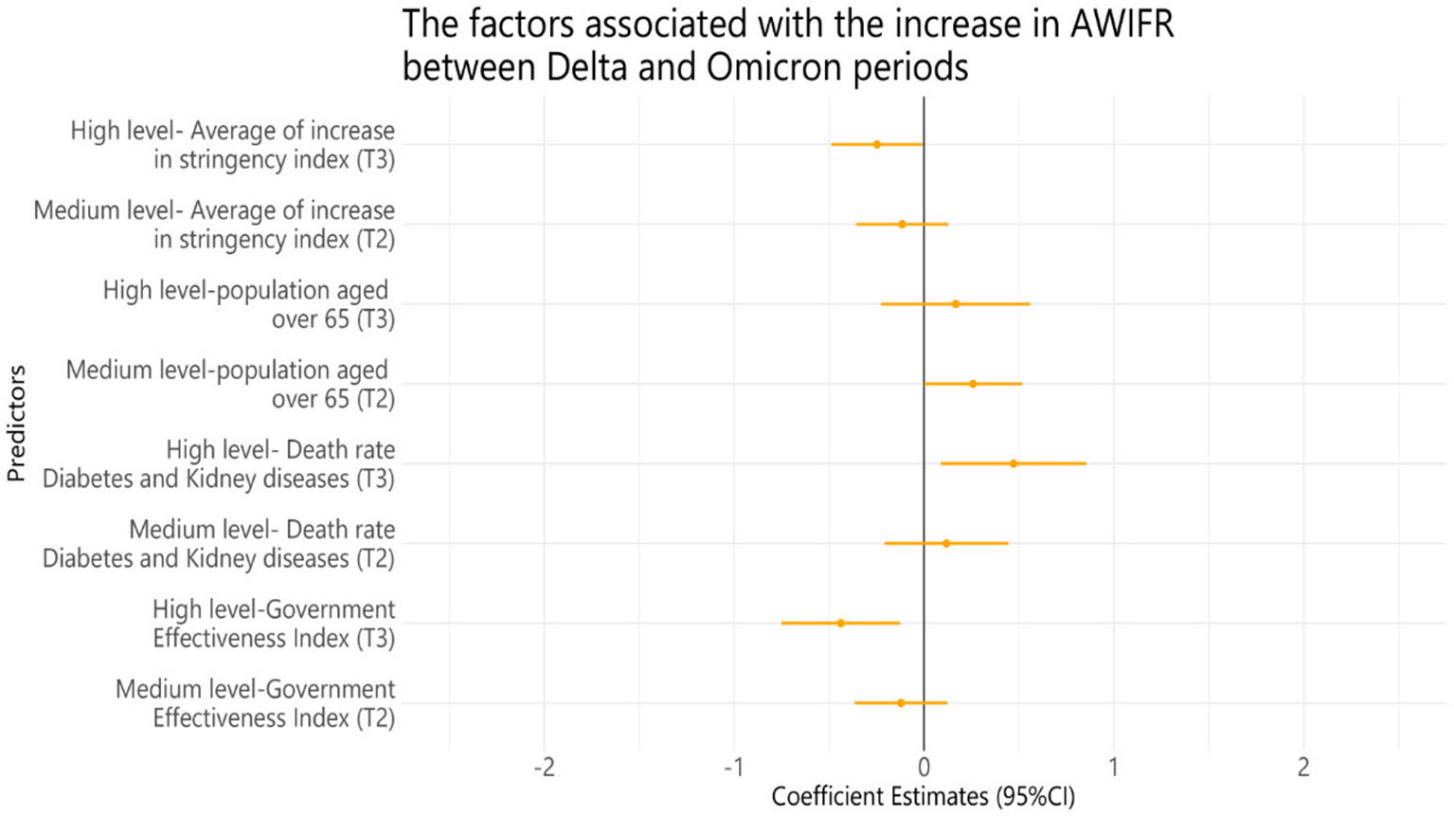
Multivariable linear regression model of factors associated with the increase in AWIFR between Delta and Omicron periods. Tertile 1, Low level; Tertile 2, Medium level; Tertile 3, High level. The average increase in stringency index = average stringency index in Omicron—average stringency index in Delta; a high level will increase the intensity.

Figure 8 presents the results of the multivariable linear regression model to identify the factors associated with the increase in AWIFR over two periods of Delta and Omicron. We observed that 1 unit increase in high government effectiveness and stringency indices was associated with a decrease of 0.438 [95% CI (−0.750)–(−0.126)] and 0.247 [95% CI (−0.487)–(−0.007)] unit AWIFR, respectively; 1 unit increase in death rates caused by diabetes and kidney was associated with an increase of 0.472 unit AWIFR (95% CI 0.089–0.855), and 1% increase in the population older than 65 years was associated with an increase of 0.407% AWIFR (95% CI 0.013–0.802).

Adjusted for GDP per capita, life expectancy, median age, rule of law index, GHS index, HAQ index, number of physicians per 1,000, number of nurses and midwives, hospital beds, country income, the increase in % population vaccinated with a booster dose, death rate caused by diabetes diseases, death rate caused by non-communicable diseases, death rate caused by cardiovascular diseases, death rate caused by iron deficiency, death rate caused by vitamin A deficiency, death rate caused by zinc deficiency, death rate caused by PM.25, and YLDs caused by low bone density.

## Discussion

Our analysis of the first 12 weeks during the Delta and Omicron variant periods showed that the Omicron period was found to have a lower AWIFR than the Delta period, although it exhibited greater rates of average weekly infection and average weekly fatality. The higher government effectiveness indicators and the proportion of the fully vaccinated population were significantly associated with a lower AWIFR during the Delta variant period. Meanwhile, higher death rates caused by cardiovascular diseases were linked to higher AWIFR during the Delta variant period. Over the Omicron period, a high proportion of the population vaccinated with a booster dose had a significantly lower AWIFR, whereas the proportion of the population older than 65 years, countries with low-income level, and YLDs caused by metabolic diseases were linked to greater AWIFR. Over two periods of Delta and Omicron, we found a lower increase in AWIFR in countries with high levels of government effectiveness index and stringency index. In contrast, the countries with high death rates caused by diabetes and kidney and the proportion of people older than 65 years were linked to a greater increase in AWIFR.

Government indicators were negatively associated with AWIFR in the Delta period and the increase in AWIFR between the two periods of the Delta and Omicron. The countries with a high government effectiveness index had lower AWIFR and a lower increase in AWIFR. Our results are consistent with those of previous studies (15, 28). Government effectiveness measures perceptions of the quality of public services, the quality of the civil service and the degree of its independence from political pressures, the quality of policy formulation and implementation, and the credibility of the government's commitment to such policies (29). A higher score on the government effectiveness index indicated greater effectiveness. As the COVID-19 pandemic has imposed a massive influence on society, enhancing government effectiveness plays a vital role in integrating different resources to control the COVID-19 pandemic successfully. Some previous studies indicated that a higher level of government trust had significant associations with adherence to public health remedies and vaccine coverage (15, 28). We found AWIFR was statistically significant with the population fully vaccinated during the Delta period and the population vaccinated with booster dose during the Omicron period, which is consistent with previous studies. The vaccine coverage was associated with a lower case fatality rate (28), and a booster dose effectively prevented symptomatic Omicron infection, COVID-19–related hospitalization, and death due to Omicron infection (18, 30). Therefore, maintaining vaccine supply, ensuring vaccine distribution equitably around the world, and mitigating vaccine hesitancy and barriers to accessing vaccines are of great importance in fostering the rates of vaccine coverage (1, 31). As a result, governments should enhance socioeconomic components and develop community credibility to enable effective and coordinated policy responses.

The region and country income were found to be influential factors on the AWIFR during the Delta and Omicron periods, respectively. The result showed that in comparison with the Americas, the largest decrease in AWIFR was witnessed in the African region. Our finding is supported by previous studies (32, 33). Strong sunlight exposure, which is a major source of vitamin D, may contribute to a reduction in the case of fatality in African countries compared with others (34–36). In addition, one of the explanations for the low case fatality rates and relatively moderate impacts of the COVID-19 pandemic in Africa has been hypothesized as the experience of public health response to past lethal infectious diseases, as well as cross-immunity from other coronaviruses (37). Countries with lower incomes were likely to witness higher AWIFR. This is in alignment with previous studies (7, 38).

Regarding global health burden, our results expressed that the burden of diseases (mortality rate, YLDs) caused by cardiovascular diseases, metabolism disorders, and diabetes and kidney diseases were the factors associated with the AWIFR during the Delta and Omicron periods. The countries with high death rates caused by diabetes and kidney diseases were linked to a higher AWIFR and greater increase in AWIFR than other counterparts. Some previous studies demonstrated comparable results that COVID-19 patients previously diagnosed with chronic comorbidities such as diabetes, acute kidney injury, cancer, cardiovascular disease, and chronic renal disease had a higher probability of mortality and morbidity (30, 39, 40). In addition, earlier studies demonstrated that metabolomics disorders provided new insight into the host response to COVID-19 infection and disease severity (41, 42). Therefore, people with chronic conditions need to be closely monitored by healthcare staff. Moreover, policymakers were suggested to develop appropriate policies and initiatives to safeguard COVID-19 patients and mitigate the severe COVID-19 outcomes (43).

Our finding indicated that older age was linked to a greater AWIFR during the Omicron period and a larger increase in AWIFR over two periods of the Delta and Omicron, which is consistent with previous studies (44–46). Older patients with COVID-19 are more likely to suffer from severe diseases (47), and an increase in the number of patients with severe diseases could strain the nation's economy and healthcare capacity. As a result, policymakers should consider the high level of interest in COVID-19 among older patients. Regarding NPIs, our study found a lower increase in AWIFR among countries with high levels of the average increase in the intensity of NPIs between two periods of the Delta and Omicron. This finding is supported by previous studies (48, 49). Zhou et al. (41) highlighted the significance of continued high-intensity NPIs in the low level of vaccine coverage. An increasing number of NPIs in vaccination coverage deficiency might contribute to the increased infections and mortality burden (50). With the current situation of the COVID-19 pandemic, governments are implementing opening border control to alleviate burdens on the countries' economy, so it is essential to raise people's awareness and achieve widespread vaccination to contain successfully the COVID-19 pandemic.

### Implication of this finding

Our findings highlighted that vaccination campaigns have had a major positive impact on health benefits in certain countries. It is imperative to increase vaccine coverage and eliminate disparities in vaccine coverage to lessen COVID-19 mortality and burden worldwide. Our analysis emphasized the importance of high level of government effectiveness because the association between vaccine coverage and AWIFR reductions was statistically significant only for countries with high level of government effectiveness. Among countries with equal vaccination rates, the governments with greater effectiveness might identify high-risk individuals and distribute vaccines more effectively so that the population-level advantages are maximized. In addition, the results indicated that YLDs and death rates of chronic diseases were associated with an increase in AWIFR; hence, governments should offer proper intervention treatments to patient groups at risk of severe chronic diseases. Furthermore, knowing the factors that drive the propagation of outbreaks would be a crucial component of the plan for preventing future coronavirus-related outbreaks. This study has several limitations. First, the study mainly gathered national data and reported at the regional level, the state level was not included in our analysis. Additional studies within the state level would be informative and comprehensive when these data become accessible. Second, age standardization, which is beneficial to make a comparison in terms of the disease outcomes among nations, was not possible in our analysis as each country reported the results using different age standards, and some nations where data on age groups are restricted and missing. However, we added age-representing variables in the analysis models to adjust the differences in age structure between countries. Further studies are necessary to take into account age standardization to provide evidence about disease outcomes in countries. Another limitation is that number of infections and fatalities was underreported in the dataset. In addition, our study might disregard some factors influencing COVID-19 cases and vaccination rates, such as vaccine hesitancy and public trust levels during the pandemic. Further research was vital for deeper insight when these data become approachable. Finally, since this is an ecological study that can be affected by sociodemographic factors, our results should be considered as the reference point for further studies.

## Conclusion

Although Omicron has been emphasized as a less harmful variant, it has caused unusual morbidity and mortality due to enhanced transmissibility and rapid spread. Our study indicated that the Omicron period was found to witness a lower AWIFR than the Delta period; however, it exhibited greater rates of average weekly infection and average weekly fatality. Thus, it is essential to raise the community's awareness of the severity of the Omicron, and governments are suggested to monitor COVID-19 indicators and new variants actively. Higher AWIFR mortality records were observed in countries with high death rates caused by chronic diseases and YLDs caused by metabolic disorders. Lower AWIFR and increase in AWIFR were seen among countries with a high proportion of the population vaccinated with two doses and booster dose, government effectiveness index, and stringency index. Therefore, the government should foster vaccine coverage rates, coordinate the intensity of NPIs, strengthen interventions for chronic diseases, and implement more assessments on metabolic disorders to alleviate COVID-19 outcomes.

This study encourages further studies focused on the impacts of COVID-19 variant periods, especially new subtypes of the Omicron variants and other novel variants, to provide new insights into COVID-19 infection and fatality on a global scale, and how to contain outbreaks substantially using measures with minimal socioeconomic impacts. In addition, we suggest that monitoring mortality and morbidity with real-time, reliable, and actionable data is necessary and should be maintained to timely respond to the new waves of the pandemic and the emergence of dangerous variants. Hence, there is a need to construct monitoring systems for the COVID-19 pandemic at the national, regional, and global levels; this can assist policymakers in assessing manifold factors to develop strategies and provide remedies to contain the negative impact of novel variants on the people's health, economy, and society.

## Data availability statement

The original contributions presented in the study are included in the article/Supplementary material, further inquiries can be directed to the corresponding author.

## Ethics statement

The study's ethical review and approval were exempted by the Taipei Medical University-Joint Institutional Review Board due to all data retrieved from publicly open data (TMU-JIRB No.: N202108037).

## Author contributions

NTHN, C-LS, Y-MC, T-YO, LDH, and C-CH: conceptualization and methodology. NTHN, LDH, and T-PP: formal analysis. NTHN and LDH: data curation and visualization. NTHN, C-LS, Y-MC, T-YO, LDH, T-PP, and C-CH: writing-original draft preparation and writing-reviewing and editing. All authors have read and agreed to the published version of the manuscript.

## Supplementary material

Multimedia Appendix 1 Supplementary tables.

Multimedia Appendix 2 Linear mixed-effects models of factors associated with the average weekly infection fatality rate; linear regression models of factors associated with the average weekly infection rate and average weekly fatality rate.

## References

[1] Patel MD Rosenstrom E Lvy JS Mayorga ME Keskinocak P Boyce RM. Association of simulated COVID-19 vaccination and nonpharmaceutical interventions with infections, hospitalizations, and mortality. J Am Med Assoc Netw Open. (2021) 4:e2110782. 10.1001/jamanetworkopen.2021.10782 34061203 PMC8170542

[2] Staerk C Wistuba T Mayr A. Estimating effective infection fatality rates during the course of the COVID-19 pandemic in Germany. BMC Public Health. (2021) 21:1073. 10.1186/s12889-021-11127-7 34090392 PMC8178670

[3] Johns Hopkins University. Johns Hopkins Coronavirus Resource Center Pandemic Data Initiative. (2019). Available online at: https://coronavirus.jhu.edu/pandemic-data-initiative (accessed January 12, 2023).

[4] Dyer O. COVID-19: Omicron is causing more infections but fewer hospital admissions than delta, South African data show. Br Med J. (2021) 375:3104. 10.1136/bmj.n3104 34916213

[5] Karim SSA Karim QA. Omicron SARS-CoV-2 variant: A new chapter in the COVID-19 pandemic. Lancet. (2021) 398:2126–8. 10.1016/S0140-6736(21)02758-6 34871545 PMC8640673

[6] Arceo-Gomez EO Campos-Vazquez RM Esquivel G Alcaraz E Martinez LA Lopez NG. The income gradient in COVID-19 mortality and hospitalisation: An observational study with social security administrative records in Mexico. Lancet Reg Health Am. (2022) 6:100115. 10.1016/j.lana.2021.100115 34778865 PMC8578731

[7] Willett BJ Grove J Maclean OA Wilkie C Lorenzo GD Furnon W. SARS-CoV-2 Omicron is an immune escape variant with an altered cell entry pathway. Nat Microbiol. (2022) 7:1161–79. 10.1038/s41564-022-01143-7 35798890 PMC9352574

[8] Hyams C Challen R Marlow R Nguyen J Begier E Southern J. Severity of Omicron (B11529) and Delta (B16172) SARS-CoV-2 infection among hospitalised adults: A prospective cohort study in Bristol, United Kingdom. Lancet Reg Health Eur. (2023) 25:100556. 10.1101/2022.06.29.22277044 36530491 PMC9742675

[9] Global Health Security. Global Health Security Index. (2020). Available online at: https://www.ghsindex.org (accessed April 14, 2022).

[10] GHD. Institute for Health Metrics and Evalution GBD Results Tool. University of Washington. (2017). Available online at: https://ghdx.healthdata.org/gbd-results-tool (accessed April 14, 2022).

[11] World Health Organization. World Health Organization Global Strategy on Human Resources for Health: Workforce 2030. Geneva: World Health Organization (2016).

[12] Bates D Mächler M Bolker B Walker S. Fitting linear mixed-effects models using lme4. J Stat Softw. (2015) 67:1–48. 10.18637/jss.v067.i01

[13] Hale T Angrist N Goldszmidt R Kira B Petherick A Phillips T. A global panel database of pandemic policies (Oxford COVID-19 Government Response Tracker). Nat Hum Behav. (2021) 5:529–38. 10.1038/s41562-021-01079-8 33686204

[14] GISAID. GISAID Initiative. (2020). Available online at: https://www.gisaid.org/ (accessed April 14, 2022).

[15] Mathieu E Ritchie H Ortiz-Ospina E Roser M Hasell J Appel C. A global database of COVID-19 vaccinations. Nat Hum Behav. (2021) 5:947–53. 10.1038/s41562-021-01122-8 33972767

[16] Liang LL Kao CT Ho HJ Wu CY. COVID-19 case doubling time associated with non-pharmaceutical interventions and vaccination: A global experience. J Glob Health. (2021) 11:05021. 10.7189/jogh.11.05021 34552726 PMC8442574

[17] World Bank. Worldwide Governance Indicators. (2022). Available online at: http://info.worldbank.org/governance/wgi/ (accessed April 14, 2022).

[18] Wang H Paulson KR Pease SA Watson S Comfort H Zheng P. Estimating excess mortality due to the COVID-19 pandemic: A systematic analysis of COVID-19-related mortality, 2020-21. Lancet. (2022) 399:1513–36. 10.1016/S0140-6736(21)02796-3 35279232 PMC8912932

[19] Lin TY Liao SH Lai CC Paci E Chuang SY. Effectiveness of non-pharmaceutical interventions and vaccine for containing the spread of COVID-19: Three illustrations before and after vaccination periods. J Formos Med Assoc. (2021) 16:S46–56. 10.1016/j.jfma.2021.05.015 34112587 PMC8148434

[20] Abu-Raddad LJ Chemaitelly H Ayoub HH AlMukdad S Yassine HM Al-Khatib HA. Effect of mRNA vaccine boosters against SARS-CoV-2 Omicron infection in Qatar. N Engl J Med. (2022) 386:1804–16. 10.1056/NEJMoa2200797 35263534 PMC8929389

[21] Burki TK. Challenges in the rollout of COVID-19 vaccines worldwide. Lancet Respir Med. (2021) 9:e42–3. 10.1016/S2213-2600(21)00129-6 33684355 PMC8009608

[22] Okonji EF Okonji OC Mukumbang FC Van Wyk B. Understanding varying COVID-19 mortality rates reported in Africa compared to Europe, Americas and Asia. Trop Med Int Health. (2021) 26:716–9. 10.1111/tmi.13575 33733568 PMC8251241

[23] Rice BL Annapragada L Baker RE Bruijning M Dotse-Gborgbortsi W Mensah K. Variation in SARS-CoV-2 outbreaks across sub-Saharan Africa. Nat Med. (2021) 27:447–53. 10.1038/s41591-021-01234-8 33531710 PMC8590469

[24] Asyary A Veruswati M. Sunlight exposure increased COVID-19 recovery rates: A study in the central pandemic area of Indonesia. Sci Total Environ. (2020) 729:139016. 10.1016/j.scitotenv.2020.139016 32361458 PMC7184988

[25] Ali N. Role of vitamin D in preventing of COVID-19 infection, progression and severity. J Infect Public Health. (2020) 13:1373–80. 10.1016/j.jiph.2020.06.021 32605780 PMC7305922

[26] Whittemore PB. COVID-19 fatalities, latitude, sunlight, and vitamin D. Am J Infect Control. (2020) 48:1042–4. 10.1016/j.ajic.2020.06.193 32599103 PMC7319635

[27] Mwai P. Coronavirus in Africa: Concern Grows Over Third Wave of Infections. (2021). Available online at: https://www.bbc.com/news/world-africa-53181555 (accessed April 14, 2022).

[28] Decoster A Minten T Spinnewijn J. The income gradient in mortality during the COVID-19 crisis: Evidence from Belgium. J Econ Inequal. (2021) 19:551–70. 10.1007/s10888-021-09505-7 34466135 PMC8390079

[29] Almeida-Pititto BD Dualib PM Zajdenverg A Dantas JR de Souza FD Rodacki M. Severity and mortality of COVID 19 in patients with diabetes, hypertension and cardiovascular disease: A meta-analysis. Diabetol Metabol Syndr. (2020) 12:75. 10.1186/s13098-020-00586-4 32874207 PMC7456786

[30] Dessie ZG Zewotir T A. Mortality-related risk factors of COVID-19: A systematic review and meta-analysis of 42 studies and 423,117 patients. BMC Infect Dis. (2021) 21:855. 10.1186/s12879-021-06536-3 34418980 PMC8380115

[31] Li H Wang L Zhang M Lu Y Wang W. Effects of vaccination and non-pharmaceutical interventions and their lag times on the COVID-19 pandemic: Comparison of eight countries. PLoS Negl Trop Dis. (2022) 16:e0010101. 10.1371/journal.pntd.0010101 35025865 PMC8757886

[32] Migaud M Gandotra S Chand HS Gillespie MN Thannickal VJ Langley RJ. Metabolomics to predict antiviral drug efficacy in COVID-19. Am J Respir Cell Mol Biol. (2020) 63:396–8. 10.1165/rcmb.2020-0206LE 32574504 PMC7462337

[33] Casari I Manfredi M Metharom P Falasca M. Dissecting lipid metabolism alterations in SARS-CoV-2. Prog Lipid Res. (2021) 82:101092. 10.1016/j.plipres.2021.101092 33571544 PMC7869689

[34] Ellen N McKee M. Caring-for-People-With-Chronic-Conditions. England: International Journal of Integrated Care. (2008).

[35] COVID-19 Forecasting Team. Variation in the COVID-19 infection fatality ratio by age, time, and geography during the pre-vaccine era: A systematic analysis. Lancet. (2022) 399:1469–88. 10.1016/S0140-6736(21)02867-1 35219376 PMC8871594

[36] Yanez ND Weiss NS Romand JA Treggiari MM. COVID-19 mortality risk for older men and women. BMC Public Health. (2020) 20:1742. 10.1186/s12889-020-09826-8 33213391 PMC7675386

[37] Mallapaty S. The coronavirus is most deadly if you are older and male—New data reveal the risks. Nature. (2020) 585:16–7. 10.1038/d41586-020-02483-2 32860026

[38] Liu K Chen Y Lin R Han K. Clinical features of COVID-19 in elderly patients: A comparison with young and middle-aged patients. J Infect. (2020) 80:e14–8. 10.1016/j.jinf.2020.03.005 32171866 PMC7102640

[39] Bo Y Guo C Lin C Zeng Y Li HB Zhang Y. Effectiveness of non-pharmaceutical interventions on COVID-19 transmission in 190 countries from 23 January to 13 April 2020. Int J Infect Dis. (2021) 102:247–53. 10.1016/j.ijid.2020.10.066 33129965 PMC7598763

[40] Liu Y Morgenstern C Kelly J Lowe R Jit M. The impact of non-pharmaceutical interventions on SARS-CoV-2 transmission across 130 countries and territories. BMC Med. (2021) 19:40. 10.1186/s12916-020-01872-8 33541353 PMC7861967

[41] Zhou F Hu TJ Zhang XY Lai K Chen JH Zhou XY. The association of intensity and duration of non-pharmacological interventions and implementation of vaccination with COVID-19 infection, death, and excess mortality: Natural experiment in 22 European countries. J Infect Public Health. (2022) 15:499–507. 10.1016/j.jiph.2022.03.011 35429788 PMC8944114

[42] Ioannidis JPA. Infection fatality rate of COVID-19 inferred from seroprevalence data. Bull World Health Organ. (2021) 99:19–33f. 10.2471/BLT.20.265892 33716331 PMC7947934

[43] Coccia M. Preparedness of countries to face COVID-19 pandemic crisis: Strategic positioning and factors supporting effective strategies of prevention of pandemic threats. Environ Res. (2022) 203:111678. 10.1016/j.envres.2021.111678 34280421 PMC8284056

[44] European Center for Disease Prevention Control. COVID-19-Assessment-Further-Emergence-Omicron-19th-Risk-Assessment-December-2021. (2021). Available online at: https://www.ecdc.europa.eu/en/publications-data/covid-19-omicron-risk-assessment-further-emergence-and-potential-impact (accessed May 25, 2022).

[45] Menni C Valdes PM Polidori L Antonelli M Penamakuri S Nogal A. *S*ymptom prevalence, duration, and risk of hospital admission in individuals infected with SARS-CoV-2 during periods of omicron and delta variant dominance: A prospective observational study from the ZOE COVID Study. Lancet. (2022) 399:1618–24. 10.1016/S0140-6736(22)00327-0 35397851 PMC8989396

[46] Kim J Hong K Yum S Gómez Gómez RE Jang J Park SH. Factors associated with the difference between the incidence and case-fatality ratio of coronavirus disease 2019 by country. Sci Rep. (2021) 11:18938. 10.1038/s41598-021-98378-x 34556739 PMC8460795

[47] Duong BV Larpruenrudee P Fang T Hossain SI Saha SC Gu Y. Is the SARS CoV-2 Omicron variant deadlier and more transmissible than delta variant. Int J Environ Res Public Health. (2022) 19:4586. 10.3390/ijerph19084586 35457468 PMC9032753

[48] World Bank. World Development Indicators, World Bank Collection. (2017). Available online at: https://databank.worldbank.org/source/world-development-indicators (accessed April 14, 2022).

[49] Mathieu E Ritchie H Rodes-Guirao L Appel C Gavrilov D Giattino C. Coronavirus Pandemic (COVID-19). (2020). Available online at: https://ourworldindata.org/coronavirus (Accessed April 14, 2022).

[50] Elbe S Buckland-Merrett G. Data, disease and diplomacy: GISAID's innovative contribution to global health. Glob Chall. (2017) 1:33–46. 10.1002/gch2.1018 31565258 PMC6607375

