# Supplementary material for: A global analysis of COVID-19 infection fatality rate and its associated factors during the Delta and Omicron variant periods: an ecological study
Source: Front Public Health. 2023 Jun 2;11:1145138. doi: 10.3389/fpubh.2023.1145138 (PMC10274323; doi:10.3389/fpubh.2023.1145138)
Supplement: Multimedia Appendix 1 — Supplementary tables. [file Data_Sheet_1.docx]

**Multimedia Appendix**

**Multimedia Appendix 1.** Supplementary tables.

**Table S1**. List of variables and their data sources.

|  | Variables | Range value | Values recorded | Data source |
| --- | --- | --- | --- | --- |
|  | Population | No applicable | No applicable | World Bank Open Data Link: https://data.worldbank.org/ |
|  | Population density | No applicable | No applicable |  |
|  | Life expectancy | No applicable | No applicable |  |
|  | GDP per capita  (USD per capita) | No applicable | No applicable |  |
|  | % population aged over 65 | No applicable | No applicable |  |
|  | % Urban population | No applicable | No applicable |  |
|  | Median age | No applicable | No applicable |  |
|  | Rule of Law | 1 - 5 | No applicable | Worldwide Governance Indicators  http://info.worldbank.org/govern-ance/wgi |
|  | Regulatory Quality | 1 -5 | No applicable |  |
|  | Government Effectiveness | -2.5 - 2.5 | No applicable |  |
|  | UHC index | 0-100 | No applicable | Global Health Observatory indicator views https://apps.who.int/gho/data/node.imr 2019 |
|  | GHS index | 0-100 | No applicable | World Health Organization <https://www.ghsindex.org> |
|  | HAQ index | 0-100 | No applicable | Healthcare Access and Quality Index Based on Amenable Mortality http://ghdx.healthdata.org |
|  | No. Physician per 1000 | No applicable | No applicable | World Bank Open Data  https://data.worldbank.org |
|  | No. Nurse and Midwife per 1000 | No applicable | No applicable |  |
|  | % GDP for health expenditure | No applicable | No applicable |  |
|  | PM2.5 | No applicable | No applicable |  |
|  | Tobacco | No applicable | No applicable |  |
|  | Non-communicable diseases | No applicable | No applicable | Global Burden of disease  <https://www.healthdata.org/gbd/2019>  (We retrieved the death rate per 100000 inhabitants and years live with disability (YLDs) per 100000 inhabitants ) |
|  | Diabetes | No applicable | No applicable |  |
|  | Chronic respiratory disease | No applicable | No applicable |  |
|  | Cancer | No applicable | No applicable |  |
|  | Chronic kidney diseases | No applicable | No applicable |  |
|  | Cardiovascular diseases | No applicable | No applicable |  |
|  | Diabetes and kidney diseases | No applicable | No applicable |  |
|  | Zinc deficiency | No applicable | No applicable |  |
|  | Vitamin A deficiency | No applicable | No applicable |  |
|  | Low bone density | No applicable | No applicable |  |
|  | Metabolism disorders | No applicable | No applicable |  |
|  | Stringency index | 0 - 100 | No applicable | Oxford database: The Oxford COVID-19 Government Response Tracker (OxCGRT) https://www.bsg.ox.ac.uk/research/research-projects/covid-19-government-response-tracker |
|  | % population vaccinated at least one dose | No applicable | No applicable | Oxford database: A global database of COVID-19 vaccinations  <https://ourworldindata.org/covid-vaccinations> |
|  | % population fully vaccinated | No applicable | No applicable |  |
|  | % population vaccinated the booster dose | No applicable | No applicable |  |

**Table S2.** Characteristics of 110 countries by region and income levels.

| **Characteristics** | **n** | **%** | ***P*-value** |
| --- | --- | --- | --- |
| **Region** |  |  | 0.05 |
| Americas | 23 | 20.9 |  |
| South-East Asia | 18 | 16.4 |  |
| Europe | 33 | 30.0 |  |
| Africa | 36 | 32.7 |  |
| **Income level** |  |  | 0.312 |
| High income | 42 | 38.2 |  |
| Upper middle income | 30 | 27.3 |  |
| Low income | 38 | 35.5 |  |

**Table S3.** The selected countries during the Delta variant period and Omicron variant period

| **ISO Code** | **Country name** | **Delta variant period** | | **Omicron variant period** | |
| --- | --- | --- | --- | --- | --- |
|  |  | **The first date** | **The last date** | **The first date** | **The last date** |
| ABW | Aruba | 11/07/2021 | 03/10/2021 | 09/01/2022 | 03/04/2022 |
| ARG | Argentina |  |  | 26/12/2021 | 20/03/2022 |
| AUS | Australia | 09/05/2021 | 01/08/2021 | 26/12/2021 | 20/03/2022 |
| AUT | Austria | 27/06/2021 | 19/09/2021 | 26/12/2021 | 20/03/2022 |
| BEL | Belgium | 27/06/2021 | 19/09/2021 | 02/01/2022 | 27/03/2022 |
| BEN | Benin |  |  | 05/12/2021 | 27/02/2022 |
| BFA | Burkina Faso | 29/08/2021 | 21/11/2021 | 19/12/2021 | 13/03/2022 |
| BGD | Bangladesh | 16/05/2021 | 08/08/2021 | 09/01/2022 | 03/04/2022 |
| BGR | Bulgaria | 04/07/2021 | 26/09/2021 | 16/01/2022 | 10/04/2022 |
| BIH | Bosnia and Herzegovina | 20/06/2021 | 12/09/2021 | 09/01/2022 | 03/04/2022 |
| BLR | Belarus | 08/08/2021 | 31/10/2021 | 09/01/2022 | 03/04/2022 |
| BLZ | Belize | 07/03/2021 | 30/05/2021 | 26/12/2021 | 20/03/2022 |
| BOL | Bolivia | 03/10/2021 | 26/12/2021 | 09/01/2022 | 03/04/2022 |
| BRA | Brazil | 15/08/2021 | 07/11/2021 | 26/12/2021 | 20/03/2022 |
| BWA | Botswana |  |  | 12/12/2021 | 06/03/2022 |
| CAF | Central African Republic | |  | 09/01/2022 | 03/04/2022 |
| CAN | Canada | 11/07/2021 | 03/10/2021 | 19/12/2021 | 13/03/2022 |
| CHE | Switzerland | 04/07/2021 | 26/09/2021 | 26/12/2021 | 20/03/2022 |
| CHL | Chile | 12/09/2021 | 05/12/2021 | 26/12/2021 | 20/03/2022 |
| CIV | Côte d'Ivoire |  |  | 26/12/2021 | 20/03/2022 |
| CMR | Cameroon | 06/06/2021 | 29/08/2021 | 12/12/2021 | 06/03/2022 |
| COD | Democratic Republic of Congo | 04/04/2021 | 27/06/2021 | 19/12/2021 | 13/03/2022 |
| COG | Congo | 14/03/2021 | 06/06/2021 | 19/12/2021 | 13/03/2022 |
| COL | Colombia | 12/09/2021 | 05/12/2021 | 26/12/2021 | 20/03/2022 |
| CPV | Cape Verde | 27/06/2021 | 19/09/2021 | 26/12/2021 | 20/03/2022 |
| CRI | Costa Rica | 25/07/2021 | 17/10/2021 | 26/12/2021 | 20/03/2022 |
| CZE | Czechia | 27/06/2021 | 19/09/2021 | 02/01/2022 | 27/03/2022 |
| DEU | Germany | 27/06/2021 | 19/09/2021 | 02/01/2022 | 27/03/2022 |
| DJI | Djibouti | 04/07/2021 | 26/09/2021 | 19/12/2021 | 13/03/2022 |
| DNK | Denmark | 04/07/2021 | 26/09/2021 | 26/12/2021 | 20/03/2022 |
| DOM | Dominican Republic | 14/02/2021 | 09/05/2021 | 26/12/2021 | 20/03/2022 |
| ECU | Ecuador | 29/08/2021 | 21/11/2021 | 26/12/2021 | 20/03/2022 |
| ESP | Spain | 04/07/2021 | 26/09/2021 | 26/12/2021 | 20/03/2022 |
| EST | Estonia | 04/07/2021 | 26/09/2021 | 09/01/2022 | 03/04/2022 |
| FIN | Finland | 20/06/2021 | 12/09/2021 | 02/01/2022 | 27/03/2022 |
| FRA | France | 04/07/2021 | 26/09/2021 | 02/01/2022 | 27/03/2022 |
| GEO | Georgia | 11/07/2021 | 03/10/2021 | 26/12/2021 | 20/03/2022 |
| GHA | Ghana | 20/06/2021 | 12/09/2021 | 28/11/2021 | 20/02/2022 |
| GMB | Gambia | 11/04/2021 | 04/07/2021 | 19/12/2021 | 13/03/2022 |
| GRC | Greece | 11/07/2021 | 03/10/2021 | 26/12/2021 | 20/03/2022 |
| GTM | Guatemala | 18/07/2021 | 10/10/2021 | 19/12/2021 | 13/03/2022 |
| HKG | Hong Kong | 02/05/2021 | 25/07/2021 | 19/12/2021 | 13/03/2022 |
| HND | Honduras | 01/08/2021 | 24/10/2021 | 19/12/2021 | 13/03/2022 |
| HRV | Croatia | 27/06/2021 | 19/09/2021 | 09/01/2022 | 03/04/2022 |
| IDN | Indonesia | 06/06/2021 | 29/08/2021 | 02/01/2022 | 27/03/2022 |
| IRL | Ireland | 27/06/2021 | 19/09/2021 | 19/12/2021 | 13/03/2022 |
| IRN | Iran | 11/07/2021 | 03/10/2021 | 28/11/2021 | 20/02/2022 |
| IRQ | Iraq | 04/07/2021 | 26/09/2021 | 26/12/2021 | 20/03/2022 |
| ISR | Israel | 06/06/2021 | 29/08/2021 | 26/12/2021 | 20/03/2022 |
| ITA | Italy | 04/07/2021 | 26/09/2021 | 02/01/2022 | 27/03/2022 |
| JAM | Jamaica | 16/05/2021 | 08/08/2021 | 26/12/2021 | 20/03/2022 |
| JOR | Jordan | 06/06/2021 | 29/08/2021 | 19/12/2021 | 13/03/2022 |
| KEN | Kenya | 06/06/2021 | 29/08/2021 | 28/11/2021 | 20/02/2022 |
| KHM | Cambodia | 01/08/2021 | 24/10/2021 | 02/01/2022 | 27/03/2022 |
| KWT | Kuwait | 06/06/2021 | 29/08/2021 | 16/01/2022 | 10/04/2022 |
| LBN | Lebanon | 18/07/2021 | 10/10/2021 | 05/12/2021 | 27/02/2022 |
| LKA | Sri Lanka | 25/07/2021 | 17/10/2021 | 02/01/2022 | 27/03/2022 |
| LTU | Lithuania | 11/07/2021 | 03/10/2021 | 09/01/2022 | 03/04/2022 |
| LUX | Luxembourg | 20/06/2021 | 12/09/2021 | 02/01/2022 | 27/03/2022 |
| MAR | Morocco |  |  | 26/12/2021 | 20/03/2022 |
| MDA | Moldova | 04/07/2021 | 26/09/2021 | 02/01/2022 | 27/03/2022 |
| MEX | Mexico | 04/07/2021 | 26/09/2021 | 26/12/2021 | 20/03/2022 |
| MLT | Malta | 04/07/2021 | 26/09/2021 | 02/01/2022 | 27/03/2022 |
| MNG | Mongolia | 02/05/2021 | 25/07/2021 | 02/01/2022 | 27/03/2022 |
| MOZ | Mozambique | 20/06/2021 | 12/09/2021 | 28/11/2021 | 20/02/2022 |
| MUS | Mauritius | 27/06/2021 | 19/09/2021 | 09/01/2022 | 03/04/2022 |
| MYS | Malaysia | 13/06/2021 | 05/09/2021 | 26/12/2021 | 20/03/2022 |
| NGA | Nigeria | 02/05/2021 | 25/07/2021 | 05/12/2021 | 27/02/2022 |
| NLD | Netherlands | 04/07/2021 | 26/09/2021 | 02/01/2022 | 27/03/2022 |
| NOR | Norway | 11/07/2021 | 03/10/2021 | 26/12/2021 | 20/03/2022 |
| NPL | Nepal | 18/04/2021 | 11/07/2021 | 26/12/2021 | 20/03/2022 |
| NZL | New Zealand | 30/05/2021 | 22/08/2021 | 09/01/2022 | 03/04/2022 |
| OMN | Oman | 10/01/2021 | 04/04/2021 | 05/12/2021 | 27/02/2022 |
| PAK | Pakistan | 23/05/2021 | 15/08/2021 | 26/12/2021 | 20/03/2022 |
| PAN | Panama | 05/09/2021 | 28/11/2021 | 26/12/2021 | 20/03/2022 |
| PER | Peru | 12/09/2021 | 05/12/2021 | 02/01/2022 | 27/03/2022 |
| PHL | Philippines | 25/07/2021 | 17/10/2021 | 05/12/2021 | 27/02/2022 |
| PNG | Papua New Guinea | 04/07/2021 | 26/09/2021 | 09/01/2022 | 03/04/2022 |
| POL | Poland | 04/07/2021 | 26/09/2021 | 16/01/2022 | 10/04/2022 |
| PRT | Portugal | 23/05/2021 | 15/08/2021 | 26/12/2021 | 20/03/2022 |
| PRY | Paraguay | 01/08/2021 | 24/10/2021 | 26/12/2021 | 20/03/2022 |
| QAT | Qatar | 30/05/2021 | 22/08/2021 | 26/12/2021 | 20/03/2022 |
| ROU | Romania | 27/06/2021 | 19/09/2021 | 02/01/2022 | 27/03/2022 |
| RUS | Russia | 23/05/2021 | 15/08/2021 |  |  |
| SEN | Senegal | 04/07/2021 | 26/09/2021 | 05/12/2021 | 27/02/2022 |
| SGP | Singapore | 25/04/2021 | 18/07/2021 | 19/12/2021 | 13/03/2022 |
| SLB | Solomon Islands | |  | 02/01/2022 | 27/03/2022 |
| SLV | El Salvador |  |  | 26/12/2021 | 20/03/2022 |
| SRB | Serbia | 06/09/2020 | 29/11/2020 | 26/12/2021 | 20/03/2022 |
| SSD | South Sudan | 23/05/2021 | 15/08/2021 | 19/12/2021 | 13/03/2022 |
| SUR | Suriname | 22/08/2021 | 14/11/2021 | 02/01/2022 | 27/03/2022 |
| SVK | Slovakia | 14/02/2021 | 09/05/2021 | 16/01/2022 | 10/04/2022 |
| SVN | Slovenia | 11/07/2021 | 03/10/2021 | 02/01/2022 | 27/03/2022 |
| SWE | Sweden | 04/07/2021 | 26/09/2021 | 26/12/2021 | 20/03/2022 |
| SWZ | Eswatini | 27/06/2021 | 19/09/2021 | 28/11/2021 | 20/02/2022 |
| SYC | Seychelles | 30/05/2021 | 22/08/2021 | 26/12/2021 | 20/03/2022 |
| TCD | Chad | 11/07/2021 | 03/10/2021 | 02/01/2022 | 27/03/2022 |
| THA | Thailand | 04/07/2021 | 26/09/2021 | 26/12/2021 | 20/03/2022 |
| TTO | Trinidad and Tobago | 10/10/2021 | 02/01/2022 | 16/01/2022 | 10/04/2022 |
| TUN | Tunisia | 13/06/2021 | 05/09/2021 | 16/01/2022 | 10/04/2022 |
| TUR | Turkey | 27/06/2021 | 19/09/2021 | 09/01/2022 | 03/04/2022 |
| TWN | Taiwan | 04/07/2021 | 26/09/2021 | 19/12/2021 | 13/03/2022 |
| UGA | Uganda | 13/06/2021 | 05/09/2021 | 05/12/2021 | 27/02/2022 |
| UKR | Ukraine | 27/06/2021 | 19/09/2021 |  |  |
| USA | United States | 27/06/2021 | 19/09/2021 | 26/12/2021 | 20/03/2022 |
| VEN | Venezuela | 15/08/2021 | 07/11/2021 |  |  |
| VNM | Vietnam | 25/04/2021 | 18/07/2021 | 09/01/2022 | 03/04/2022 |
| ZAF | South Africa | 13/06/2021 | 05/09/2021 | 21/11/2021 | 13/02/2022 |
| ZMB | Zambia | 23/05/2021 | 15/08/2021 | 05/12/2021 | 27/02/2022 |
| ZWE | Zimbabwe | 27/06/2021 | 19/09/2021 | 28/11/2021 | 20/02/2022 |

**Table S4.** The stringency index during the first twelve weeks of the Delta and Omicron variant periods.

| **Iso code** | **Country** | **Delta period** | | | **Omicron period** | | |
| --- | --- | --- | --- | --- | --- | --- | --- |
|  |  | **The first date of stringency index** | **The last date of stringency index** | **Average Stringency index over period** | **The first date of stringency index** | **The last date of stringency index** | **Average Stringency index over period** |
| ABW | Aruba | 27/06/2021 | 19/09/2021 | 28.76 | 09/01/2022 | 03/04/2022 | 31.85 |
| ARG | Argentina |  |  |  | 26/12/2021 | 20/03/2022 | 44.94 |
| AUS | Australia | 25/04/2021 | 18/07/2021 | 68.43 | 26/12/2021 | 20/03/2022 | 51.19 |
| AUT | Austria | 13/06/2021 | 05/09/2021 | 55.90 | 26/12/2021 | 20/03/2022 | 57.59 |
| BEL | Belgium | 13/06/2021 | 05/09/2021 | 48.25 | 02/01/2022 | 27/03/2022 | 49.74 |
| BEN | Benin |  |  |  | 05/12/2021 | 27/02/2022 | 33.33 |
| BFA | Burkina Faso | 15/08/2021 | 07/11/2021 | 13.89 | 19/12/2021 | 13/03/2022 | 12.25 |
| BGD | Bangladesh | 02/05/2021 | 25/07/2021 | 63.39 | 09/01/2022 | 03/04/2022 | 42.21 |
| BGR | Bulgaria | 20/06/2021 | 12/09/2021 | 40.77 | 16/01/2022 | 10/04/2022 | 40.41 |
| BIH | Bosnia and Herzegovina | 06/06/2021 | 29/08/2021 | 36.39 | 09/01/2022 | 03/04/2022 | 38.29 |
| BLR | Belarus | 25/07/2021 | 17/10/2021 | 28.52 | 09/01/2022 | 03/04/2022 | 25.48 |
| BLZ | Belize | 21/02/2021 | 16/05/2021 | 56.28 | 26/12/2021 | 20/03/2022 | 55.97 |
| BOL | Bolivia | 19/09/2021 | 12/12/2021 | 38.93 | 09/01/2022 | 03/04/2022 | 39.13 |
| BRA | Brazil | 01/08/2021 | 24/10/2021 | 51.84 | 26/12/2021 | 20/03/2022 | 60.01 |
| BWA | Botswana |  |  |  | 12/12/2021 | 06/03/2022 | 61.53 |
| CAF | Central African Republic |  |  |  | 09/01/2022 | 03/04/2022 | 28.96 |
| CAN | Canada | 27/06/2021 | 19/09/2021 | 64.06 | 19/12/2021 | 13/03/2022 | 64.10 |
| CHE | Switzerland | 20/06/2021 | 12/09/2021 | 48.45 | 26/12/2021 | 20/03/2022 | 46.74 |
| CHL | Chile | 29/08/2021 | 21/11/2021 | 52.44 | 26/12/2021 | 20/03/2022 | 38.81 |
| CIV | Côte d'Ivoire |  |  |  | 26/12/2021 | 20/03/2022 | 20.67 |
| CMR | Cameroon | 23/05/2021 | 15/08/2021 | 33.48 | 12/12/2021 | 06/03/2022 | 26.53 |
| COD | Democratic Republic of Congo | 21/03/2021 | 13/06/2021 | 39.48 | 19/12/2021 | 13/03/2022 | 36.39 |
| COG | Congo | 28/02/2021 | 23/05/2021 | 39.34 | 19/12/2021 | 13/03/2022 | 45.12 |
| COL | Colombia | 29/08/2021 | 21/11/2021 | 44.76 | 26/12/2021 | 20/03/2022 | 56.62 |
| CPV | Cape Verde | 13/06/2021 | 05/09/2021 | 52.24 | 26/12/2021 | 20/03/2022 | 58.49 |
| CRI | Costa Rica | 11/07/2021 | 03/10/2021 | 52.84 | 26/12/2021 | 20/03/2022 | 50.44 |
| CZE | Czechia | 13/06/2021 | 05/09/2021 | 37.52 | 02/01/2022 | 27/03/2022 | 45.00 |
| DEU | Germany | 13/06/2021 | 05/09/2021 | 58.44 | 02/01/2022 | 27/03/2022 | 50.49 |
| DJI | Djibouti | 20/06/2021 | 12/09/2021 | 47.44 | 19/12/2021 | 13/03/2022 | 49.98 |
| DNK | Denmark | 20/06/2021 | 12/09/2021 | 41.90 | 26/12/2021 | 20/03/2022 | 32.10 |
| DOM | Dominican Republic | 31/01/2021 | 25/04/2021 | 52.26 | 26/12/2021 | 20/03/2022 | 32.12 |
| ECU | Ecuador | 15/08/2021 | 07/11/2021 | 57.85 | 26/12/2021 | 20/03/2022 | 52.48 |
| ESP | Spain | 20/06/2021 | 12/09/2021 | 63.73 | 26/12/2021 | 20/03/2022 | 50.87 |
| EST | Estonia | 20/06/2021 | 12/09/2021 | 31.72 | 09/01/2022 | 03/04/2022 | 48.95 |
| FIN | Finland | 06/06/2021 | 29/08/2021 | 37.12 | 02/01/2022 | 27/03/2022 | 35.49 |
| FRA | France | 20/06/2021 | 12/09/2021 | 58.55 | 02/01/2022 | 27/03/2022 | 59.27 |
| GEO | Georgia | 27/06/2021 | 19/09/2021 | 42.75 | 26/12/2021 | 20/03/2022 | 49.14 |
| GHA | Ghana | 06/06/2021 | 29/08/2021 | 40.11 | 28/11/2021 | 20/02/2022 | 43.27 |
| GMB | Gambia | 28/03/2021 | 20/06/2021 | 35.33 | 19/12/2021 | 13/03/2022 | 18.83 |
| GRC | Greece | 27/06/2021 | 19/09/2021 | 67.56 | 26/12/2021 | 20/03/2022 | 59.05 |
| GTM | Guatemala | 04/07/2021 | 26/09/2021 | 57.51 | 19/12/2021 | 13/03/2022 | 57.57 |
| HKG | Hong Kong | 18/04/2021 | 11/07/2021 | 65.53 | 19/12/2021 | 13/03/2022 | 61.54 |
| HND | Honduras | 18/07/2021 | 10/10/2021 | 65.03 | 19/12/2021 | 13/03/2022 | 45.05 |
| HRV | Croatia | 13/06/2021 | 05/09/2021 | 41.40 | 09/01/2022 | 03/04/2022 | 38.85 |
| IDN | Indonesia | 23/05/2021 | 15/08/2021 | 64.16 | 02/01/2022 | 27/03/2022 | 61.74 |
| IRL | Ireland | 13/06/2021 | 05/09/2021 | 50.43 | 19/12/2021 | 13/03/2022 | 37.17 |
| IRN | Iran | 27/06/2021 | 19/09/2021 | 64.47 | 28/11/2021 | 20/02/2022 | 56.17 |
| IRQ | Iraq | 20/06/2021 | 12/09/2021 | 75.32 | 26/12/2021 | 20/03/2022 | 54.94 |
| ISR | Israel | 23/05/2021 | 15/08/2021 | 45.26 | 26/12/2021 | 20/03/2022 | 42.95 |
| ITA | Italy | 20/06/2021 | 12/09/2021 | 57.81 | 02/01/2022 | 27/03/2022 | 67.83 |
| JAM | Jamaica | 02/05/2021 | 25/07/2021 | 73.49 | 26/12/2021 | 20/03/2022 | 71.97 |
| JOR | Jordan | 23/05/2021 | 15/08/2021 | 56.84 | 19/12/2021 | 13/03/2022 | 36.80 |
| KEN | Kenya | 23/05/2021 | 15/08/2021 | 63.93 | 28/11/2021 | 20/02/2022 | 48.10 |
| KHM | Cambodia | 18/07/2021 | 10/10/2021 | 57.96 | 02/01/2022 | 27/03/2022 | 36.52 |
| KWT | Kuwait | 23/05/2021 | 15/08/2021 | 57.67 | 16/01/2022 | 10/04/2022 | 39.30 |
| LBN | Lebanon | 04/07/2021 | 26/09/2021 | 56.52 | 05/12/2021 | 27/02/2022 | 47.49 |
| LKA | Sri Lanka | 11/07/2021 | 03/10/2021 | 76.30 | 02/01/2022 | 27/03/2022 | 50.12 |
| LTU | Lithuania | 27/06/2021 | 19/09/2021 | 39.91 | 09/01/2022 | 03/04/2022 | 26.35 |
| LUX | Luxembourg | 06/06/2021 | 29/08/2021 | 35.69 | 02/01/2022 | 27/03/2022 | 45.41 |
| MAR | Morocco |  |  |  | 26/12/2021 | 20/03/2022 | 62.97 |
| MDA | Moldova | 20/06/2021 | 12/09/2021 | 41.71 | 02/01/2022 | 27/03/2022 | 41.14 |
| MEX | Mexico | 20/06/2021 | 12/09/2021 | 54.19 | 26/12/2021 | 20/03/2022 | 42.35 |
| MLT | Malta | 20/06/2021 | 12/09/2021 | 45.67 | 02/01/2022 | 27/03/2022 | 48.41 |
| MNG | Mongolia | 18/04/2021 | 11/07/2021 | 45.22 | 02/01/2022 | 27/03/2022 | 13.65 |
| MOZ | Mozambique | 06/06/2021 | 29/08/2021 | 53.34 | 28/11/2021 | 20/02/2022 | 37.13 |
| MUS | Mauritius | 13/06/2021 | 05/09/2021 | 43.31 | 09/01/2022 | 03/04/2022 | 46.02 |
| MYS | Malaysia | 30/05/2021 | 22/08/2021 | 69.11 | 26/12/2021 | 20/03/2022 | 54.46 |
| NGA | Nigeria | 18/04/2021 | 11/07/2021 | 55.95 | 05/12/2021 | 27/02/2022 | 41.74 |
| NLD | Netherlands | 20/06/2021 | 12/09/2021 | 42.39 | 02/01/2022 | 27/03/2022 | 45.96 |
| NOR | Norway | 27/06/2021 | 19/09/2021 | 39.67 | 26/12/2021 | 20/03/2022 | 35.76 |
| NPL | Nepal | 04/04/2021 | 27/06/2021 | 71.44 | 26/12/2021 | 20/03/2022 | 48.18 |
| NZL | New Zealand | 16/05/2021 | 08/08/2021 | 39.94 | 09/01/2022 | 03/04/2022 | 58.01 |
| OMN | Oman | 27/12/2020 | 21/03/2021 | 62.62 | 05/12/2021 | 27/02/2022 | 64.94 |
| PAK | Pakistan | 09/05/2021 | 01/08/2021 | 66.13 | 26/12/2021 | 20/03/2022 | 61.01 |
| PAN | Panama | 22/08/2021 | 14/11/2021 | 67.87 | 26/12/2021 | 20/03/2022 | 50.54 |
| PER | Peru | 29/08/2021 | 21/11/2021 | 61.56 | 02/01/2022 | 27/03/2022 | 55.29 |
| PHL | Philippines | 11/07/2021 | 03/10/2021 | 71.29 | 05/12/2021 | 27/02/2022 | 72.45 |
| PNG | Papua New Guinea | 20/06/2021 | 12/09/2021 | 54.23 | 09/01/2022 | 03/04/2022 | 57.19 |
| POL | Poland | 20/06/2021 | 12/09/2021 | 41.27 | 16/01/2022 | 10/04/2022 | 36.41 |
| PRT | Portugal | 09/05/2021 | 01/08/2021 | 56.40 | 26/12/2021 | 20/03/2022 | 32.35 |
| PRY | Paraguay | 18/07/2021 | 10/10/2021 | 48.02 | 26/12/2021 | 20/03/2022 | 35.33 |
| QAT | Qatar | 16/05/2021 | 08/08/2021 | 52.08 | 26/12/2021 | 20/03/2022 | 55.00 |
| ROU | Romania | 13/06/2021 | 05/09/2021 | 48.85 | 02/01/2022 | 27/03/2022 | 42.55 |
| RUS | Russia | 09/05/2021 | 01/08/2021 | 41.02 |  |  |  |
| SEN | Senegal | 20/06/2021 | 12/09/2021 | 30.82 | 05/12/2021 | 27/02/2022 | 27.08 |
| SGP | Singapore | 11/04/2021 | 04/07/2021 | 51.34 | 19/12/2021 | 13/03/2022 | 51.23 |
| SLB | Solomon Islands |  |  |  | 02/01/2022 | 27/03/2022 | 71.08 |
| SLV | El Salvador |  |  |  | 26/12/2021 | 20/03/2022 | 41.05 |
| SRB | Serbia | 23/08/2020 | 15/11/2020 | 39.68 | 26/12/2021 | 20/03/2022 | 32.73 |
| SSD | South Sudan | 09/05/2021 | 01/08/2021 | 31.72 | 19/12/2021 | 13/03/2022 | 37.30 |
| SUR | Suriname | 08/08/2021 | 31/10/2021 | 58.53 | 02/01/2022 | 27/03/2022 | 55.97 |
| SVK | Slovakia | 31/01/2021 | 25/04/2021 | 45.92 | 16/01/2022 | 10/04/2022 | 43.47 |
| SVN | Slovenia | 27/06/2021 | 19/09/2021 | 33.16 | 02/01/2022 | 27/03/2022 | 42.03 |
| SWE | Sweden | 20/06/2021 | 12/09/2021 | 38.20 | 26/12/2021 | 20/03/2022 | 36.53 |
| SWZ | Eswatini | 13/06/2021 | 05/09/2021 | 58.89 | 28/11/2021 | 20/02/2022 | 56.63 |
| SYC | Seychelles | 16/05/2021 | 08/08/2021 | 80.57 | 26/12/2021 | 20/03/2022 | 68.22 |
| TCD | Chad | 27/06/2021 | 19/09/2021 | 34.70 | 02/01/2022 | 27/03/2022 | 38.46 |
| THA | Thailand | 20/06/2021 | 12/09/2021 | 53.79 | 26/12/2021 | 20/03/2022 | 45.20 |
| TTO | Trinidad and Tobago | 26/09/2021 | 19/12/2021 | 62.66 | 16/01/2022 | 10/04/2022 | 51.88 |
| TUN | Tunisia | 30/05/2021 | 22/08/2021 | 71.89 | 16/01/2022 | 10/04/2022 | 37.20 |
| TUR | Turkey | 13/06/2021 | 05/09/2021 | 54.42 | 09/01/2022 | 03/04/2022 | 44.49 |
| TWN | Taiwan | 20/06/2021 | 12/09/2021 | 50.15 | 19/12/2021 | 13/03/2022 | 31.38 |
| UGA | Uganda | 30/05/2021 | 22/08/2021 | 66.43 | 05/12/2021 | 27/02/2022 | 48.84 |
| UKR | Ukraine | 13/06/2021 | 05/09/2021 | 57.80 |  |  |  |
| USA | United States | 13/06/2021 | 05/09/2021 | 53.59 | 26/12/2021 | 20/03/2022 | 57.50 |
| VEN | Venezuela | 01/08/2021 | 24/10/2021 | 62.47 |  |  |  |
| VNM | Vietnam | 11/04/2021 | 04/07/2021 | 72.19 | 09/01/2022 | 03/04/2022 | 61.26 |
| ZAF | South Africa | 30/05/2021 | 22/08/2021 | 64.86 | 21/11/2021 | 13/02/2022 | 50.90 |
| ZMB | Zambia | 09/05/2021 | 01/08/2021 | 47.13 | 05/12/2021 | 27/02/2022 | 37.08 |
| ZWE | Zimbabwe | 13/06/2021 | 05/09/2021 | 65.06 | 28/11/2021 | 20/02/2022 | 45.19 |

**Table S5.** The vaccination period during the first twelve weeks of the Delta and Omicron variant periods.

| **Iso code** | **Country name** | **Delta period** | | **Omicron period** | |
| --- | --- | --- | --- | --- | --- |
|  |  | **The first date of vaccination** | **The last date of vaccination** | **The first date of vaccination** | **The last date of vaccination** |
| ABW | Aruba | 01/06/2021 | 24/08/2021 | 30/11/2021 | 22/02/2022 |
| ARG | Argentina |  |  | 16/11/2021 | 08/02/2022 |
| AUS | Australia | 30/03/2021 | 22/06/2021 | 16/11/2021 | 08/02/2022 |
| AUT | Austria | 18/05/2021 | 10/08/2021 | 16/11/2021 | 08/02/2022 |
| BEL | Belgium | 18/05/2021 | 10/08/2021 | 23/11/2021 | 15/02/2022 |
| BEN | Benin |  |  | 26/10/2021 | 18/01/2022 |
| BFA | Burkina Faso | 20/07/2021 | 12/10/2021 | 09/11/2021 | 01/02/2022 |
| BGD | Bangladesh | 06/04/2021 | 29/06/2021 | 30/11/2021 | 22/02/2022 |
| BGR | Bulgaria | 25/05/2021 | 17/08/2021 | 07/12/2021 | 01/03/2022 |
| BIH | Bosnia and Herzegovina | 11/05/2021 | 03/08/2021 | 30/11/2021 | 22/02/2022 |
| BLR | Belarus | 29/06/2021 | 21/09/2021 | 30/11/2021 | 22/02/2022 |
| BLZ | Belize | 26/01/2021 | 20/04/2021 | 16/11/2021 | 08/02/2022 |
| BOL | Bolivia | 24/08/2021 | 16/11/2021 | 30/11/2021 | 22/02/2022 |
| BRA | Brazil | 06/07/2021 | 28/09/2021 | 16/11/2021 | 08/02/2022 |
| BWA | Botswana |  |  | 02/11/2021 | 25/01/2022 |
| CAF | Central African Republic |  |  | 30/11/2021 | 22/02/2022 |
| CAN | Canada | 01/06/2021 | 24/08/2021 | 09/11/2021 | 01/02/2022 |
| CHE | Switzerland | 25/05/2021 | 17/08/2021 | 16/11/2021 | 08/02/2022 |
| CHL | Chile | 03/08/2021 | 26/10/2021 | 16/11/2021 | 08/02/2022 |
| CIV | Côte d'Ivoire |  |  | 16/11/2021 | 08/02/2022 |
| CMR | Cameroon | 27/04/2021 | 20/07/2021 | 02/11/2021 | 25/01/2022 |
| COD | Democratic Republic of Congo | 23/02/2021 | 18/05/2021 | 09/11/2021 | 01/02/2022 |
| COG | Congo | 02/02/2021 | 27/04/2021 | 09/11/2021 | 01/02/2022 |
| COL | Colombia | 03/08/2021 | 26/10/2021 | 16/11/2021 | 08/02/2022 |
| CPV | Cape Verde | 18/05/2021 | 10/08/2021 | 16/11/2021 | 08/02/2022 |
| CRI | Costa Rica | 15/06/2021 | 07/09/2021 | 16/11/2021 | 08/02/2022 |
| CZE | Czechia | 18/05/2021 | 10/08/2021 | 23/11/2021 | 15/02/2022 |
| DEU | Germany | 18/05/2021 | 10/08/2021 | 23/11/2021 | 15/02/2022 |
| DJI | Djibouti | 25/05/2021 | 17/08/2021 | 09/11/2021 | 01/02/2022 |
| DNK | Denmark | 25/05/2021 | 17/08/2021 | 16/11/2021 | 08/02/2022 |
| DOM | Dominican Republic | 05/01/2021 | 30/03/2021 | 16/11/2021 | 08/02/2022 |
| ECU | Ecuador | 20/07/2021 | 12/10/2021 | 16/11/2021 | 08/02/2022 |
| ESP | Spain | 25/05/2021 | 17/08/2021 | 16/11/2021 | 08/02/2022 |
| EST | Estonia | 25/05/2021 | 17/08/2021 | 30/11/2021 | 22/02/2022 |
| FIN | Finland | 11/05/2021 | 03/08/2021 | 23/11/2021 | 15/02/2022 |
| FRA | France | 25/05/2021 | 17/08/2021 | 23/11/2021 | 15/02/2022 |
| GEO | Georgia | 01/06/2021 | 24/08/2021 | 16/11/2021 | 08/02/2022 |
| GHA | Ghana | 11/05/2021 | 03/08/2021 | 19/10/2021 | 11/01/2022 |
| GMB | Gambia | 02/03/2021 | 25/05/2021 | 09/11/2021 | 01/02/2022 |
| GRC | Greece | 01/06/2021 | 24/08/2021 | 16/11/2021 | 08/02/2022 |
| GTM | Guatemala | 08/06/2021 | 31/08/2021 | 09/11/2021 | 01/02/2022 |
| HKG | Hong Kong | 23/03/2021 | 15/06/2021 | 09/11/2021 | 01/02/2022 |
| HND | Honduras | 22/06/2021 | 14/09/2021 | 09/11/2021 | 01/02/2022 |
| HRV | Croatia | 18/05/2021 | 10/08/2021 | 30/11/2021 | 22/02/2022 |
| IDN | Indonesia | 27/04/2021 | 20/07/2021 | 23/11/2021 | 15/02/2022 |
| IRL | Ireland | 18/05/2021 | 10/08/2021 | 09/11/2021 | 01/02/2022 |
| IRN | Iran | 01/06/2021 | 24/08/2021 | 19/10/2021 | 11/01/2022 |
| IRQ | Iraq | 25/05/2021 | 17/08/2021 | 16/11/2021 | 08/02/2022 |
| ISR | Israel | 27/04/2021 | 20/07/2021 | 16/11/2021 | 08/02/2022 |
| ITA | Italy | 25/05/2021 | 17/08/2021 | 23/11/2021 | 15/02/2022 |
| JAM | Jamaica | 06/04/2021 | 29/06/2021 | 16/11/2021 | 08/02/2022 |
| JOR | Jordan | 27/04/2021 | 20/07/2021 | 09/11/2021 | 01/02/2022 |
| KEN | Kenya | 27/04/2021 | 20/07/2021 | 19/10/2021 | 11/01/2022 |
| KHM | Cambodia | 22/06/2021 | 14/09/2021 | 23/11/2021 | 15/02/2022 |
| KWT | Kuwait | 27/04/2021 | 20/07/2021 | 07/12/2021 | 01/03/2022 |
| LBN | Lebanon | 08/06/2021 | 31/08/2021 | 26/10/2021 | 18/01/2022 |
| LKA | Sri Lanka | 15/06/2021 | 07/09/2021 | 23/11/2021 | 15/02/2022 |
| LTU | Lithuania | 01/06/2021 | 24/08/2021 | 30/11/2021 | 22/02/2022 |
| LUX | Luxembourg | 11/05/2021 | 03/08/2021 | 23/11/2021 | 15/02/2022 |
| MAR | Morocco |  |  | 16/11/2021 | 08/02/2022 |
| MDA | Moldova | 25/05/2021 | 17/08/2021 | 23/11/2021 | 15/02/2022 |
| MEX | Mexico | 25/05/2021 | 17/08/2021 | 16/11/2021 | 08/02/2022 |
| MLT | Malta | 25/05/2021 | 17/08/2021 | 23/11/2021 | 15/02/2022 |
| MNG | Mongolia | 23/03/2021 | 15/06/2021 | 23/11/2021 | 15/02/2022 |
| MOZ | Mozambique | 11/05/2021 | 03/08/2021 | 19/10/2021 | 11/01/2022 |
| MUS | Mauritius | 18/05/2021 | 10/08/2021 | 30/11/2021 | 22/02/2022 |
| MYS | Malaysia | 04/05/2021 | 27/07/2021 | 16/11/2021 | 08/02/2022 |
| NGA | Nigeria | 23/03/2021 | 15/06/2021 | 26/10/2021 | 18/01/2022 |
| NLD | Netherlands | 25/05/2021 | 17/08/2021 | 23/11/2021 | 15/02/2022 |
| NOR | Norway | 01/06/2021 | 24/08/2021 | 16/11/2021 | 08/02/2022 |
| NPL | Nepal | 09/03/2021 | 01/06/2021 | 16/11/2021 | 08/02/2022 |
| NZL | New Zealand | 20/04/2021 | 13/07/2021 | 30/11/2021 | 22/02/2022 |
| OMN | Oman | 01/12/2020 | 23/02/2021 | 26/10/2021 | 18/01/2022 |
| PAK | Pakistan | 13/04/2021 | 06/07/2021 | 16/11/2021 | 08/02/2022 |
| PAN | Panama | 27/07/2021 | 19/10/2021 | 16/11/2021 | 08/02/2022 |
| PER | Peru | 03/08/2021 | 26/10/2021 | 23/11/2021 | 15/02/2022 |
| PHL | Philippines | 15/06/2021 | 07/09/2021 | 26/10/2021 | 18/01/2022 |
| PNG | Papua New Guinea | 25/05/2021 | 17/08/2021 | 30/11/2021 | 22/02/2022 |
| POL | Poland | 25/05/2021 | 17/08/2021 | 07/12/2021 | 01/03/2022 |
| PRT | Portugal | 13/04/2021 | 06/07/2021 | 16/11/2021 | 08/02/2022 |
| PRY | Paraguay | 22/06/2021 | 14/09/2021 | 16/11/2021 | 08/02/2022 |
| QAT | Qatar | 20/04/2021 | 13/07/2021 | 16/11/2021 | 08/02/2022 |
| ROU | Romania | 18/05/2021 | 10/08/2021 | 23/11/2021 | 15/02/2022 |
| RUS | Russia | 13/04/2021 | 06/07/2021 |  |  |
| SEN | Senegal | 25/05/2021 | 17/08/2021 | 26/10/2021 | 18/01/2022 |
| SGP | Singapore | 16/03/2021 | 08/06/2021 | 09/11/2021 | 01/02/2022 |
| SLB | Solomon Islands |  |  | 23/11/2021 | 15/02/2022 |
| SLV | El Salvador |  |  | 16/11/2021 | 08/02/2022 |
| SRB | Serbia | 28/07/2020 | 20/10/2020 | 16/11/2021 | 08/02/2022 |
| SSD | South Sudan | 13/04/2021 | 06/07/2021 | 09/11/2021 | 01/02/2022 |
| SUR | Suriname | 13/07/2021 | 05/10/2021 | 23/11/2021 | 15/02/2022 |
| SVK | Slovakia | 05/01/2021 | 30/03/2021 | 07/12/2021 | 01/03/2022 |
| SVN | Slovenia | 01/06/2021 | 24/08/2021 | 23/11/2021 | 15/02/2022 |
| SWE | Sweden | 25/05/2021 | 17/08/2021 | 16/11/2021 | 08/02/2022 |
| SWZ | Eswatini | 18/05/2021 | 10/08/2021 | 19/10/2021 | 11/01/2022 |
| SYC | Seychelles | 20/04/2021 | 13/07/2021 | 16/11/2021 | 08/02/2022 |
| TCD | Chad | 01/06/2021 | 24/08/2021 | 23/11/2021 | 15/02/2022 |
| THA | Thailand | 25/05/2021 | 17/08/2021 | 16/11/2021 | 08/02/2022 |
| TTO | Trinidad and Tobago | 31/08/2021 | 23/11/2021 | 07/12/2021 | 01/03/2022 |
| TUN | Tunisia | 04/05/2021 | 27/07/2021 | 07/12/2021 | 01/03/2022 |
| TUR | Turkey | 18/05/2021 | 10/08/2021 | 30/11/2021 | 22/02/2022 |
| TWN | Taiwan | 25/05/2021 | 17/08/2021 | 09/11/2021 | 01/02/2022 |
| UGA | Uganda | 04/05/2021 | 27/07/2021 | 26/10/2021 | 18/01/2022 |
| UKR | Ukraine | 18/05/2021 | 10/08/2021 |  |  |
| USA | United States | 18/05/2021 | 10/08/2021 | 16/11/2021 | 08/02/2022 |
| VEN | Venezuela | 06/07/2021 | 28/09/2021 |  |  |
| VNM | Vietnam | 16/03/2021 | 08/06/2021 | 30/11/2021 | 22/02/2022 |
| ZAF | South Africa | 04/05/2021 | 27/07/2021 | 12/10/2021 | 04/01/2022 |
| ZMB | Zambia | 13/04/2021 | 06/07/2021 | 26/10/2021 | 18/01/2022 |
| ZWE | Zimbabwe | 18/05/2021 | 10/08/2021 | 19/10/2021 | 11/01/2022 |

**Table S6.** The vaccination value during the first twelve weeks of the Delta and Omicron variant periods.

| **ISO Code** | **Country name** | **Delta period** | | | **Omicron period** | | |
| --- | --- | --- | --- | --- | --- | --- | --- |
|  |  | At least one dose in the twelfth week | Fully vaccinated in the twelfth  week | Booster dose in the twelfth week | At least one dose in the twelfth week | Fully vaccinated in the twelfth week | Booster dose in the  twelfth week |
| ABW | Aruba | 75.99 | 69.87 | 0 | 82.02 | 75.96 | 0 |
| AUS | Australia | 53.93 | 33.82 | 0 | 85.94 | 81.17 | 48.41 |
| AUT | Austria | 63.37 | 60.01 | 0.25 | 75.37 | 72.89 | 56.3 |
| BEL | Belgium | 73.83 | 72.07 | 0.14 | 79.37 | 78.48 | 62.87 |
| BFA | Burkina Faso | 1.7 | 1.38 | 0 | 9.85 | 5.36 | 0 |
| BGD | Bangladesh | 10.61 | 4.29 | 0 | 76.9 | 66.85 | 5.69 |
| BGR | Bulgaria | 15.79 | 18.97 | 0 | 30.21 | 29.76 | 10.6 |
| BIH | Bosnia and Herzegovina | 19.43 | 13.06 | 0 | 28.91 | 25.93 | 3.69 |
| BLR | Belarus | 29.23 | 21.25 | 0 | 62.23 | 57.25 | 5.96 |
| BLZ | Belize | 55.27 | 35.69 | 0 | 58.19 | 51.51 | 10.3 |
| BOL | Bolivia | 46.18 | 38.18 | 4.28 | 59.92 | 49.1 | 10.49 |
| BRA | Brazil | 74.97 | 55.92 | 4.74 | 84.27 | 74.22 | 34.21 |
| CAN | Canada | 76.58 | 71.22 | 0.53 | 85.62 | 81.48 | 47.35 |
| CHE | Switzerland | 63.13 | 57.54 | 0.01 | 69.73 | 68.72 | 42.15 |
| CHL | Chile | 88.01 | 84.48 | 46.75 | 92.95 | 90.25 | 80.93 |
| CMR | Cameroon | 1.28 | 0.29 | 0 | 3.94 | 2.99 | 0 |
| COD | Democratic Republic of the Congo | 0.09 | 0.02 | 0 | 0.82 | 0.48 | 0 |
| COG | Republic of the Congo | 4.93 | 2.11 | 0 | 12.03 | 11.31 | 0 |
| COL | Colombia | 58.85 | 49.1 | 0 | 81.32 | 66.96 | 18.94 |
| CPV | Cabo Verde | 50.56 | 29.57 | 0 | 63.02 | 54.44 | 0 |
| CRI | Costa Rica | 68.6 | 46.62 | 0 | 83.16 | 75.75 | 31.6 |
| CZE | Czech Republic | 56.52 | 55.18 | 0 | 64.84 | 63.98 | 38.29 |
| DEU | Germany | 67.68 | 63.74 | 0.5 | 76.76 | 76.54 | 60.4 |
| DJI | Djibouti | 3.85 | 2.6 | 0 | 14 | 10.01 | 0 |
| DNK | Denmark | 76.04 | 74.44 | 0.95 | 83.16 | 82.46 | 61.97 |
| DOM | Dominican Republic | 61.88 | 49.16 | 11.45 | 65.47 | 53.84 | 20.76 |
| ECU | Ecuador | 74.7 | 60.58 | 1.51 | 82.88 | 76.49 | 27.86 |
| ESP | Spain | 54.17 | 37.48 | 0 | 87.95 | 85.52 | 52.01 |
| EST | Estonia | 56.89 | 53.2 | 1.75 | 64.85 | 63.64 | 34.62 |
| FIN | Finland | 73.15 | 53.71 | 0 | 81.18 | 77.55 | 51.39 |
| FRA | France | 75.26 | 65.54 | 1.56 | 80.53 | 78.08 | 53.86 |
| GEO | Georgia | 24.83 | 20.74 | 0 | 39.92 | 31.5 | 2.03 |
| GHA | Ghana | 2.73 | 1.28 | 0 | 23.55 | 14.4 | 0.32 |
| GMB | Gambia | 1.26 | 0.49 | 0 | 13.27 | 12.74 | 0 |
| GRC | Greece | 62.27 | 59.39 | 0.43 | 76.06 | 73.09 | 53.15 |
| GTM | Guatemala | 32.4 | 21.9 | 0 | 40.18 | 31.69 | 11.1 |
| HKG | Hong Kong | 55.98 | 47.28 | 0 | 84.76 | 71.48 | 29.55 |
| HND | Honduras | 39.63 | 36 | 0 | 51.58 | 45.35 | 10.47 |
| HRV | Croatia | 43.67 | 40.02 | 0 | 56.62 | 54.83 | 14.57 |
| IDN | Indonesia | 22.15 | 12.56 | 0 | 70.82 | 57.02 | 6.61 |
| IRL | Ireland | 76.18 | 73.39 | 0 | 81.64 | 80.26 | 57.42 |
| IRN | Iran | 42.7 | 18.4 | 0 | 74.72 | 66.02 | 29.26 |
| IRQ | Iraq | 11.13 | 6.04 | 0 | 25.21 | 17.59 | 0.38 |
| ISR | Israel | 64.4 | 59.2 | 21.24 | 72.16 | 65.98 | 56.38 |
| ITA | Italy | 74.39 | 67.19 | 0.09 | 84.02 | 79.19 | 64.21 |
| JAM | Jamaica | 18.37 | 10.18 | 0 | 26.12 | 22.43 | 1.06 |
| JOR | Jordan | 33.33 | 27.93 | 0 | 46.18 | 43.4 | 6.21 |
| KEN | Kenya | 3.5 | 1.46 | 0 | 14.65 | 13.39 | 0.44 |
| KHM | Cambodia | 83.94 | 80.2 | 17.95 | 87.39 | 82.4 | 53.33 |
| KWT | Kuwait | 33.55 | 21.33 | 0 | 78.87 | 75.94 | 27.22 |
| LBN | Lebanon | 24.54 | 20.08 | 0 | 37.23 | 32.64 | 8.33 |
| LKA | Sri Lanka | 68.7 | 59.17 | 0 | 79.06 | 66.93 | 35.91 |
| LTU | Lithuania | 65.17 | 61.44 | 1.13 | 72.45 | 69.63 | 34.25 |
| LUX | Luxembourg | 66.36 | 0 | 0.95 | 75.66 | 0 | 58.2 |
| MDA | Moldova | 13.94 | 19.11 | 0 | 26.92 | 26.18 | 2.06 |
| MEX | Mexico | 48.61 | 33.99 | 0 | 65.64 | 61.03 | 0 |
| MLT | Malta | 81.42 | 81.4 | 0 | 91.75 | 90.31 | 0 |
| MNG | Mongolia | 67.76 | 64.32 | 0 | 68.26 | 65.32 | 31.02 |
| MOZ | Mozambique | 5.11 | 2.37 | 0 | 36.87 | 31.85 | 0.32 |
| MUS | Mauritius | 74.41 | 71.45 | 0 | 78.57 | 75.66 | 46.53 |
| MYS | Malaysia | 60.26 | 45.14 | 0 | 83.67 | 78.68 | 47.25 |
| NGA | Nigeria | 1.3 | 0.67 | 0 | 8.1 | 3.59 | 0 |
| NLD | Netherlands | 74.61 | 67.16 | 0 | 78.37 | 72.11 | 53.05 |
| NOR | Norway | 76.59 | 67.34 | 0.39 | 79.26 | 73.67 | 53.17 |
| NPL | Nepal | 8.8 | 3.25 | 0 | 73.77 | 63.03 | 6.38 |
| NZL | New Zealand | 49.67 | 25.72 | 0 | 83.58 | 79.28 | 51.17 |
| OMN | Oman | 38.45 | 12.42 | 0 | 61.5 | 57.39 | 0.62 |
| PAK | Pakistan | 24.97 | 11.32 | 0 | 56.87 | 45.24 | 2.16 |
| PAN | Panama | 70.03 | 62.28 | 3.36 | 77.64 | 68.11 | 32.02 |
| PER | Peru | 69.55 | 58.48 | 4.44 | 86.07 | 78 | 37.42 |
| PHL | Philippines | 25.07 | 21.6 | 0 | 61.83 | 56.42 | 8.81 |
| PNG | Papua New Guinea | 1.25 | 0.44 | 0 | 3.48 | 2.82 | 0 |
| POL | Poland | 52.27 | 51.26 | 0 | 59.83 | 59.22 | 31.15 |
| PRT | Portugal | 75.48 | 68.49 | 0 | 95.04 | 92.6 | 61.11 |
| PRY | Paraguay | 39.73 | 28.51 | 0.09 | 53.45 | 45.66 | 16.89 |
| QAT | Qatar | 80.54 | 75.7 | 0 | 88.4 | 88.4 | 47.32 |
| ROU | Romania | 28.62 | 28.3 | 0 | 0 | 42.26 | 0 |
| SEN | Senegal | 7.09 | 3.29 | 0 | 8.47 | 6.04 | 0.02 |
| SGP | Singapore | 75.81 | 46.7 | 0 | 91.73 | 90.2 | 70.51 |
| SRB | Serbia | 43.93 | 41.93 | 7.18 | 48.69 | 47.54 | 27.25 |
| SSD | South Sudan | 0.46 | 0.04 | 0 | 4.18 | 3.8 | 0 |
| SUR | Suriname | 42.87 | 35.5 | 0 | 45.13 | 40.02 | 7.99 |
| SVK | Slovakia | 45.17 | 43.7 | 0 | 51.76 | 50.75 | 30.15 |
| SVN | Slovenia | 56.18 | 49.97 | 1.13 | 60.84 | 58.69 | 30.74 |
| SWE | Sweden | 69.22 | 63.03 | 0 | 76.76 | 74.75 | 0 |
| SWZ | Eswatini | 15.41 | 14.76 | 0 | 32.32 | 26.93 | 0 |
| SYC | Seychelles | 74.59 | 70.48 | 0 | 85.26 | 80.97 | 35.3 |
| TCD | Chad | 0.73 | 0.18 | 0 | 1.67 | 0.91 | 0 |
| THA | Thailand | 42.17 | 22.73 | 0.89 | 78.44 | 71.49 | 31.89 |
| TTO | Trinidad and Tobago | 50.79 | 47.67 | 5.45 | 53.13 | 50.46 | 10.26 |
| TUN | Tunisia | 36.34 | 20.94 | 0 | 60.13 | 53.22 | 9.87 |
| TUR | Turkey | 61.85 | 49.27 | 12.22 | 67.94 | 62.3 | 42.61 |
| TWN | Taiwan | 71.06 | 31.57 | 0 | 81.3 | 75.82 | 46.06 |
| UGA | Uganda | 2.19 | 0.78 | 0 | 28.72 | 5.74 | 0 |
| USA | USA | 63.91 | 56.19 | 0.89 | 77 | 65.64 | 29.42 |
| VNM | Vietnam | 5.69 | 0.63 | 0 | 81.44 | 79.2 | 46.29 |
| ZAF | South Africa | 16.22 | 10.5 | 0 | 33.22 | 28.16 | 1.27 |
| ZMB | Zambia | 1.63 | 1.12 | 0 | 0 | 9.83 | 0.15 |
| ZWE | Zimbabwe | 19.42 | 13.59 | 0 | 29 | 22.6 | 0.98 |
| RUS | Russia | 27.86 | 22.1 | 0 |  |  |  |
| UKR | Ukraine | 14.45 | 11.87 | 0 |  |  |  |
| VEN | Venezuela | 57.19 | 34.34 | 0 |  |  |  |
| ARG | Argentina |  |  |  | 89.7 | 80.88 | 40.63 |
| BEN | Benin |  |  |  | 0.39 | 0.17 | 0 |
| BWA | Botswana |  |  |  | 10.09 | 5.37 | 0 |
| CAF | Central African Republic |  |  |  | 18.77 | 18.25 | 0 |
| CIV | Côte d'Ivoire |  |  |  | 25.84 | 15.6 | 0 |
| MAR | Morocco |  |  |  | 66.6 | 62.48 | 16.04 |
| SLB | Solomon Islands |  |  |  | 32.23 | 16.46 | 0 |
| SLV | El Salvador |  |  |  | 70.23 | 65.6 | 22.4 |

**Table S7.** The AWIFR among countries during the first twelve weeks of the Delta and Omicron variant periods.

| **ISO Code** | **Country name** | **AWIFR in the Delta period** | **AWIFR in the Omicron period** |
| --- | --- | --- | --- |
| ABW | Aruba | 0.11 | 0.04 |
| AUS | Australia | 0.03 | 0.01 |
| AUT | Austria | 0.02 | 0.01 |
| BEL | Belgium | 0.02 | 0.01 |
| BFA | Burkina Faso | 0.45 | 0.16 |
| BGD | Bangladesh | 0.16 | 0.02 |
| BGR | Bulgaria | 0.28 | 0.12 |
| BIH | Bosnia and Herzegovina | 0.25 | 0.23 |
| BLR | Belarus | 0.06 | 0.04 |
| BLZ | Belize | 0.10 | 0.02 |
| BOL | Bolivia | 0.09 | 0.07 |
| BRA | Brazil | 0.22 | 0.04 |
| CAN | Canada | 0.05 | 0.04 |
| CHE | Switzerland | 0.02 | 0.01 |
| CHL | Chile | 0.08 | 0.03 |
| CMR | Cameroon | 0.14 | 0.07 |
| COD | Democratic Republic of the Congo | 0.09 | 0.08 |
| COG | Republic of the Congo | 0.16 | 0.02 |
| COL | Colombia | 0.17 | 0.08 |
| CPV | Cabo Verde | 0.09 | 0.02 |
| CRI | Costa Rica | 0.10 | 0.03 |
| CZE | Czech Republic | 0.06 | 0.02 |
| DEU | Germany | 0.04 | 0.01 |
| DJI | Djibouti | 0.14 | 0.00 |
| DNK | Denmark | 0.01 | 0.01 |
| DOM | Dominican Republic | 0.03 | 0.01 |
| ECU | Ecuador | 0.32 | 0.05 |
| ESP | Spain | 0.08 | 0.02 |
| EST | Estonia | 0.03 | 0.01 |
| FIN | Finland | 0.02 | 0.02 |
| FRA | France | 0.04 | 0.01 |
| GEO | Georgia | 0.12 | 0.04 |
| GHA | Ghana | 0.09 | 0.07 |
| GMB | Gambia | 0.21 | 0.10 |
| GRC | Greece | 0.08 | 0.03 |
| GTM | Guatemala | 0.23 | 0.05 |
| HKG | Hong Kong | 0.07 | 0.03 |
| HND | Honduras | 0.34 | 0.09 |
| HRV | Croatia | 0.08 | 0.07 |
| IDN | Indonesia | 0.30 | 0.05 |
| IRL | Ireland | 0.02 | 0.01 |
| IRN | Iran | 0.13 | 0.07 |
| IRQ | Iraq | 0.06 | 0.04 |
| ISR | Israel | 0.02 | 0.01 |
| ITA | Italy | 0.06 | 0.02 |
| JAM | Jamaica | 0.20 | 0.09 |
| JOR | Jordan | 0.13 | 0.02 |
| KEN | Kenya | 0.19 | 0.04 |
| KHM | Cambodia | 0.56 | 0.02 |
| KWT | Kuwait | 0.06 | 0.00 |
| LBN | Lebanon | 0.05 | 0.03 |
| LKA | Sri Lanka | 0.33 | 0.16 |
| LTU | Lithuania | 0.09 | 0.02 |
| LUX | Luxembourg | 0.02 | 0.01 |
| MDA | Moldova | 0.13 | 0.07 |
| MEX | Mexico | 0.32 | 0.11 |
| MLT | Malta | 0.05 | 0.06 |
| MNG | Mongolia | 0.02 | 0.00 |
| MOZ | Mozambique | 0.11 | 0.03 |
| MUS | Mauritius | 0.11 | 0.01 |
| MYS | Malaysia | 0.10 | 0.02 |
| NGA | Nigeria | 0.08 | 0.03 |
| NLD | Netherlands | 0.01 | 0.00 |
| NOR | Norway | 0.01 | 0.01 |
| NPL | Nepal | 0.14 | 0.02 |
| NZL | New Zealand | 0.01 | 0.00 |
| OMN | Oman | 0.16 | 0.01 |
| PAK | Pakistan | 0.16 | 0.05 |
| PAN | Panama | 0.13 | 0.02 |
| PER | Peru | 0.27 | 0.06 |
| PHL | Philippines | 0.10 | 0.07 |
| PNG | Papua New Guinea | 0.20 | 0.06 |
| POL | Poland | 0.18 | 0.07 |
| PRT | Portugal | 0.03 | 0.01 |
| PRY | Paraguay | 1.24 | 0.09 |
| QAT | Qatar | 0.01 | 0.00 |
| ROU | Romania | 0.15 | 0.05 |
| SEN | Senegal | 0.19 | 0.08 |
| SGP | Singapore | 0.03 | 0.00 |
| SRB | Serbia | 0.04 | 0.04 |
| SSD | South Sudan | 0.08 | 0.01 |
| SUR | Suriname | 0.16 | 0.04 |
| SVK | Slovakia | 0.03 | 0.02 |
| SVN | Slovenia | 0.03 | 0.01 |
| SWE | Sweden | 0.03 | 0.02 |
| SWZ | Eswatini | 0.16 | 0.05 |
| SYC | Seychelles | 0.06 | 0.02 |
| TCD | Chad | 0.00 | 0.05 |
| THA | Thailand | 0.09 | 0.02 |
| TTO | Trinidad and Tobago | 0.28 | 0.12 |
| TUN | Tunisia | 0.28 | 0.09 |
| TUR | Turkey | 0.07 | 0.02 |
| TWN | Taiwan | 0.51 | 0.01 |
| UGA | Uganda | 0.37 | 0.08 |
| USA | USA | 0.07 | 0.05 |
| VNM | Vietnam | 0.07 | 0.01 |
| ZAF | South Africa | 0.20 | 0.09 |
| ZMB | Zambia | 0.17 | 0.02 |
| ZWE | Zimbabwe | 0.29 | 0.08 |
| RUS | Russia | 0.27 |  |
| UKR | Ukraine | 0.21 |  |
| VEN | Venezuela | 0.09 |  |
| ARG | Argentina |  | 0.02 |
| BEN | Benin |  | 0.15 |
| BWA | Botswana |  | 0.11 |
| CAF | Central African Republic |  | 0.05 |
| CIV | Côte d'Ivoire |  | 0.04 |
| MAR | Morocco |  | 0.05 |
| SLB | Solomon Islands |  | 0.10 |
| SLV | El Salvador |  | 0.06 |

**Table S8.** The infection rate over 100,000 inhabitants and mortality rate over 100,000 inhabitants of each country during the Delta variant period.

| ISO Code | Country name | Infection rate over 100,000 inhabitants | Mortality rate over  100,000 inhabitants |
| --- | --- | --- | --- |
| ABW | Aruba | 0.0436 | 0.0006 |
| AUS | Australia | 0.4325 | 0.0018 |
| AUT | Austria | 0.721 | 0.0019 |
| BEL | Belgium | 1.3734 | 0.0034 |
| BFA | Burkina Faso | 0.0175 | 0.0009 |
| BGD | Bangladesh | 6.786 | 0.1309 |
| BGR | Bulgaria | 0.7033 | 0.0237 |
| BIH | Bosnia and Herzegovina | 0.2575 | 0.0077 |
| BLR | Belarus | 1.4425 | 0.0108 |
| BLZ | Belize | 0.1236 | 0.0015 |
| BOL | Bolivia | 0.7407 | 0.0077 |
| BRA | Brazil | 15.1054 | 0.4020 |
| CAN | Canada | 2.1153 | 0.0130 |
| CHE | Switzerland | 1.3005 | 0.0027 |
| CHL | Chile | 1.2655 | 0.0123 |
| CMR | Cameroon | 0.045 | 0.0008 |
| COD | Democratic Republic of the Congo | 0.2008 | 0.0023 |
| COG | Republic of the Congo | 0.024 | 0.0005 |
| COL | Colombia | 1.4874 | 0.0309 |
| CPV | Cabo Verde | 0.0446 | 0.0005 |
| CRI | Costa Rica | 1.5254 | 0.0186 |
| CZE | Czech Republic | 0.1899 | 0.0013 |
| DEU | Germany | 4.0807 | 0.0217 |
| DJI | Djibouti | 0.0176 | 0.0003 |
| DNK | Denmark | 0.6137 | 0.0010 |
| DOM | Dominican Republic | 0.4109 | 0.0017 |
| ECU | Ecuador | 0.2307 | 0.0089 |
| ESP | Spain | 4.73 | 0.0479 |
| EST | Estonia | 0.2188 | 0.0007 |
| FIN | Finland | 0.392 | 0.0009 |
| FRA | France | 12.0531 | 0.0528 |
| GEO | Georgia | 2.3944 | 0.0355 |
| GHA | Ghana | 0.2787 | 0.0029 |
| GMB | Gambia | 0.0295 | 0.0008 |
| GRC | Greece | 2.2136 | 0.0210 |
| GTM | Guatemala | 1.296 | 0.0365 |
| HKG | Hong Kong | 0.0024 | 0.0000 |
| HND | Honduras | 0.3405 | 0.0138 |
| HRV | Croatia | 0.3146 | 0.0029 |
| IDN | Indonesia | 22.1037 | 0.7976 |
| IRL | Ireland | 1.0288 | 0.0019 |
| IRN | Iran | 22.3825 | 0.3502 |
| IRQ | Iraq | 6.2015 | 0.0476 |
| ISR | Israel | 2.0623 | 0.0053 |
| ITA | Italy | 3.939 | 0.0300 |
| JAM | Jamaica | 0.3477 | 0.0084 |
| JOR | Jordan | 0.545 | 0.0084 |
| KEN | Kenya | 0.621 | 0.0141 |
| KHM | Cambodia | 0.1132 | 0.0076 |
| KWT | Kuwait | 0.9217 | 0.0062 |
| LBN | Lebanon | 0.7862 | 0.0050 |
| LKA | Sri Lanka | 2.3455 | 0.0937 |
| LTU | Lithuania | 0.5661 | 0.0064 |
| LUX | Luxembourg | 0.0626 | 0.0002 |
| MDA | Moldova | 0.3141 | 0.0048 |
| MEX | Mexico | 10.7905 | 0.4108 |
| MLT | Malta | 0.0643 | 0.0004 |
| MNG | Mongolia | 4.3368 | 0.0093 |
| MOZ | Mozambique | 0.7658 | 0.0104 |
| MUS | Mauritius | 0.5226 | 0.0068 |
| MYS | Malaysia | 10.687 | 0.1242 |
| NGA | Nigeria | 0.2422 | 0.0024 |
| NLD | Netherlands | 3.0747 | 0.0042 |
| NOR | Norway | 0.5703 | 0.0007 |
| NPL | Nepal | 3.6954 | 0.0628 |
| NZL | New Zealand | 0.0106 | 0.0000 |
| OMN | Oman | 0.8928 | 0.0171 |
| PAK | Pakistan | 2.7501 | 0.0514 |
| PAN | Panama | 0.1746 | 0.0028 |
| PER | Peru | 0.8156 | 0.0260 |
| PHL | Philippines | 11.6475 | 0.1336 |
| PNG | Papua New Guinea | 0.0218 | 0.0005 |
| POL | Poland | 0.2232 | 0.0049 |
| PRT | Portugal | 1.5589 | 0.0053 |
| PRY | Paraguay | 0.0798 | 0.0119 |
| QAT | Qatar | 0.1083 | 0.0001 |
| ROU | Romania | 1.7591 | 0.0318 |
| RUS | Russia | 15.4629 | 0.5030 |
| SEN | Senegal | 0.2996 | 0.0068 |
| SGP | Singapore | 0.0198 | 0.0001 |
| SRB | Serbia | 1.888 | 0.0095 |
| SSD | South Sudan | 0.0052 | 0.0001 |
| SUR | Suriname | 0.2251 | 0.0043 |
| SVK | Slovakia | 0.3227 | 0.0013 |
| SVN | Slovenia | 0.3747 | 0.0015 |
| SWE | Sweden | 0.5853 | 0.0019 |
| SWZ | Eswatini | 0.2632 | 0.0052 |
| SYC | Seychelles | 0.0816 | 0.0006 |
| TCD | Chad | 0.0009 | 0.0000 |
| THA | Thailand | 12.6622 | 0.1392 |
| TTO | Trinidad and Tobago | 0.3947 | 0.0133 |
| TUN | Tunisia | 3.0333 | 0.1026 |
| TUR | Turkey | 14.1183 | 0.1179 |
| TWN | Taiwan | 0.0062 | 0.0004 |
| UGA | Uganda | 0.5849 | 0.0261 |
| UKR | Ukraine | 1.5111 | 0.0387 |
| USA | USA | 83.9762 | 0.7103 |
| VEN | VEN | 0.8365 | 0.0094 |
| VNM | Vietnam | 1.4665 | 0.0127 |
| ZAF | South Africa | 10.6693 | 0.2558 |
| ZMB | Zambia | 1.0888 | 0.0225 |
| ZWE | Zimbabwe | 0.813 | 0.0283 |

**Table S9.** The infection rate over 100,000 inhabitants and mortality rate over 100,000 inhabitants of each country during the Omicron variant period.

| ISO Code | Country name | Infection rate over 100,000 inhabitants | Mortality rate over 100,000 inhabitants |
| --- | --- | --- | --- |
| ABW | Aruba | 0.0666 | 0.0003 |
| ARG | Argentina | 35.4648 | 0.1045 |
| AUS | Australia | 35.8966 | 0.0353 |
| AUT | Austria | 21.6446 | 0.0175 |
| BEL | Belgium | 16.7674 | 0.0236 |
| BEN | Benin | 0.004 | 0.0001 |
| BFA | Burkina Faso | 0.0408 | 0.0008 |
| BGD | Bangladesh | 3.5801 | 0.0102 |
| BGR | Bulgaria | 3.2454 | 0.0461 |
| BIH | Bosnia and Herzegovina | 0.7822 | 0.0215 |
| BLR | Belarus | 2.5639 | 0.0112 |
| BLZ | Belize | 0.2564 | 0.0006 |
| BOL | Bolivia | 2.2528 | 0.0191 |
| BRA | Brazil | 73.8117 | 0.3866 |
| BWA | Botswana | 0.6618 | 0.0090 |
| CAF | Central African Republic | 0.022 | 0.0001 |
| CAN | Canada | 14.7646 | 0.0710 |
| CHE | Switzerland | 20.4156 | 0.0126 |
| CHL | Chile | 15.814 | 0.0541 |
| CIV | Côte d'Ivoire | 0.1781 | 0.0009 |
| CMR | Cameroon | 0.1177 | 0.0010 |
| COD | Democratic Republic of the Congo | 0.2187 | 0.0021 |
| COG | Republic of the Congo | 0.0487 | 0.0001 |
| COL | Colombia | 9.559 | 0.0967 |
| CPV | Cabo Verde | 0.1685 | 0.0005 |
| CRI | Costa Rica | 2.6054 | 0.0088 |
| CZE | Czech Republic | 13.0248 | 0.0313 |
| DEU | Germany | 129.5573 | 0.1623 |
| DJI | Djibouti | 0.0203 | 0.0000 |
| DNK | Denmark | 23.1489 | 0.0219 |
| DOM | Dominican Republic | 1.6309 | 0.0014 |
| ECU | Ecuador | 3.0736 | 0.0170 |
| ESP | Spain | 56.0663 | 0.1268 |
| EST | Estonia | 3.0574 | 0.0051 |
| FIN | Finland | 5.6301 | 0.0125 |
| FRA | France | 146.7664 | 0.1769 |
| GEO | Georgia | 7.1703 | 0.0313 |
| GHA | Ghana | 0.273 | 0.0022 |
| GMB | Gambia | 0.0191 | 0.0002 |
| GRC | Greece | 17.0132 | 0.0646 |
| GTM | Guatemala | 1.8098 | 0.0107 |
| HKG | Hong Kong | 8.4631 | 0.0352 |
| HND | Honduras | 0.3804 | 0.0041 |
| HRV | Croatia | 3.3632 | 0.0271 |
| IDN | Indonesia | 17.2366 | 0.1025 |
| IRL | Ireland | 6.8523 | 0.0078 |
| IRN | Iran | 9.4878 | 0.0775 |
| IRQ | Iraq | 2.0747 | 0.0094 |
| ISR | Israel | 24.529 | 0.0222 |
| ITA | Italy | 79.7604 | 0.2105 |
| JAM | Jamaica | 0.358 | 0.0040 |
| JOR | Jordan | 6.127 | 0.0116 |
| KEN | Kenya | 0.6772 | 0.0030 |
| KHM | Cambodia | 0.1489 | 0.0004 |
| KWT | Kuwait | 1.6492 | 0.0008 |
| LBN | Lebanon | 2.7433 | 0.0096 |
| LKA | Sri Lanka | 0.7286 | 0.0144 |
| LTU | Lithuania | 4.8712 | 0.0136 |
| LUX | Luxembourg | 1.0446 | 0.0011 |
| MAR | Morocco | 2.0633 | 0.0122 |
| MDA | Moldova | 1.3578 | 0.0113 |
| MEX | Mexico | 16.7961 | 0.2317 |
| MLT | Malta | 0.225 | 0.0015 |
| MNG | Mongolia | 2.1528 | 0.0010 |
| MOZ | Mozambique | 0.7336 | 0.0025 |
| MUS | Mauritius | 1.4123 | 0.0018 |
| MYS | Malaysia | 12.3284 | 0.0300 |
| NGA | Nigeria | 0.3988 | 0.0016 |
| NLD | Netherlands | 46.3703 | 0.0097 |
| NOR | Norway | 10.039 | 0.0091 |
| NPL | Nepal | 1.5086 | 0.0037 |
| NZL | New Zealand | 6.7849 | 0.0030 |
| OMN | Oman | 0.7502 | 0.0013 |
| PAK | Pakistan | 2.2817 | 0.0142 |
| PAN | Panama | 2.7324 | 0.0074 |
| PER | Peru | 12.4218 | 0.0932 |
| PHL | Philippines | 8.2525 | 0.0697 |
| PNG | Papua New Guinea | 0.0685 | 0.0005 |
| POL | Poland | 16.6474 | 0.1333 |
| PRT | Portugal | 21.7894 | 0.0253 |
| PRY | Paraguay | 1.8194 | 0.0198 |
| QAT | Qatar | 1.1171 | 0.0006 |
| ROU | Romania | 10.2755 | 0.0610 |
| SEN | Senegal | 0.1019 | 0.0010 |
| SGP | Singapore | 7.8687 | 0.0042 |
| SLB | Solomon Islands | 0.104 | 0.0013 |
| SLV | El Salvador | 0.3931 | 0.0029 |
| SRB | Serbia | 6.6343 | 0.0308 |
| SSD | South Sudan | 0.0374 | 0.0001 |
| SUR | Suriname | 0.2626 | 0.0013 |
| SVK | Slovakia | 10.7056 | 0.0225 |
| SVN | Slovenia | 4.8892 | 0.0087 |
| SWE | Sweden | 12.0237 | 0.0279 |
| SWZ | Eswatini | 0.2243 | 0.0014 |
| SYC | Seychelles | 0.1555 | 0.0003 |
| TCD | Chad | 0.0111 | 0.0001 |
| THA | Thailand | 11.44 | 0.0267 |
| TTO | Trinidad and Tobago | 0.3898 | 0.0058 |
| TUN | Tunisia | 2.5661 | 0.0265 |
| TUR | Turkey | 49.0726 | 0.1442 |
| TWN | Taiwan | 0.0442 | 0.0000 |
| UGA | Uganda | 0.3558 | 0.0033 |
| USA | USA | 273.6609 | 1.5609 |
| VNM | Vietnam | 78.1671 | 0.0824 |
| ZAF | South Africa | 7.103 | 0.0741 |
| ZMB | Zambia | 1.023 | 0.0028 |
| ZWE | Zimbabwe | 0.7188 | 0.0066 |
