# Supplementary material for: A global analysis of COVID-19 infection fatality rate and its associated factors during the Delta and Omicron variant periods: an ecological study
Source: Front Public Health. 2023 Jun 2;11:1145138. doi: 10.3389/fpubh.2023.1145138 (PMC10274323; doi:10.3389/fpubh.2023.1145138)
Supplement: Multimedia Appendix 2 — Linear mixed-effects models of factors associated with the average weekly infection fatality rate; linear regression models of factors associated with the average weekly infection rate and average weekly fatality rate. [file Data_Sheet_2.docx]

**LONGITUDINAL ANALYSIS**

**Linear mixed-effects model of factors associated with the average weekly infection fatality rate**

**1. Model formulas for longitudinal analysis**

$$\boldsymbol{Y}_{\boldsymbol{i,t}}\boldsymbol{=}\boldsymbol{\beta}_{\boldsymbol{0}}\boldsymbol{+(\alpha+}\boldsymbol{\alpha}_{\boldsymbol{i}}\boldsymbol{)T}_{\boldsymbol{i,t}}\boldsymbol{+}\sum_{\boldsymbol{j=1}}^{\boldsymbol{n}} \boldsymbol{\beta}_{\boldsymbol{j}}\boldsymbol{X}_{\boldsymbol{ij,t}}\boldsymbol{+}\left( \boldsymbol{\theta}_{\boldsymbol{0}}\boldsymbol{P}_{\boldsymbol{i,t}} \right)\boldsymbol{+ (}\boldsymbol{\delta}_{\boldsymbol{0}}\boldsymbol{V}_{\boldsymbol{i,t}}\boldsymbol{)+}\boldsymbol{b}_{\boldsymbol{i}}\boldsymbol{+}\boldsymbol{\varepsilon}_{\boldsymbol{i,t}}$$

Our interest outcome $Y_{i,t}$ is the log transformation of average weekly infection fatality rate (AWIFR index) in the country i on the week t.

The corresponding independent variables that were classified into three equal group and significantly associated with outcome variable in univariate analysis are denoted by $X_{ij,t}$(j=1,…,n with n – number of selected independent variables by the forward selection approach) their fixed effects are captured by $\beta_{j}$.

$P_{i,t}$ is the categorical variable (tertiles) for 14-day lag stringency index of country i on week t. Their fixed effect is captured by $\theta_{0}$.

$V_{i,t}$ is the categorical variable (tertiles) for 40-day lag of percentage of population fully vaccinated or percentage of population received the booster doses in country i on week t. The effects of vaccination coverage is captured via the fixed effect $\delta_{0}$.

The time of NPI implementation (measured by stringency index) or vaccination is denoted by $T_{i,t}$ and its effect is captured via fixed effect $\alpha$.

The random intercept model was used because it enabled each country to have its own linear regression function with different intercept (corresponding to fixed intercept $\beta_{0}$). Country-specific random slopes for week $T_{i,t}$ might be important if the AWIFR has varied by different weeks. Therefore, we extended the mixed models that allows the country-specific random slopes for week effect $T_{i,t}$ with a random effect $\alpha_{i}$. Our model parameters are estimated via maximum likelihood (ML) approach.

The country-specific impact was captured via the random effect $b_{i}$~ N (0,D). $\varepsilon_{i,t}$~ N (0,$\sigma_{e}^{2}$) was the country and week specific residual error.

D is the variance-covariance matrix for the intercept and slope pertaining to the outcome Y.

**2. Model selection procedure and testing assumptions of model**

According to forward selection approach, the steps to select the final model include:

Step 1: The univariate analysis linear mixed-effects models were conducted with time and each covariate (stringency index, percentage of population fully vaccinated/ received booster doses, independent variables) as predictors

Step 2: We selected all the covariates which had significant value (p value <0.05) in the univariate analyses.

Step 3: We ranked the selected covariate in the decreasing order according to the goodness of fit of the univariate models as defined by Bayesian Information Criterion (BIC).

Step 4: We fitted a series of multivariable forward selection linear mixed-effects models with time and inputted each covariate sequentially based on its rank. If an individual variable was an insignificant predictor, it was dropped from the forward selection models.

2.1. Delta variant dominant period

*2.1.1. Model selection of the multivariable linear mixed effect model (mLME) during Delta variant dominant period*

We explored the significant covariates using the univariate analysis. Table S1 shows the selected covariates were ranked in decreasing order of good of fitness based on their BIC values.

Table S1. Univariate model results and sequence in which covariates were added in the forward selection mLME during the Delta variant dominant period

| **Rank** | **Covariates** | **Coefficient** | ***P* values** | **BIC** |
| --- | --- | --- | --- | --- |
| 1 | Intercept | -0.905 | <0.001 | 2197.139 |
|  | Medium level - Death rate of Zinc deficiency | 1.242 | <0.001 |  |
|  | High level - Death rate of Zinc deficiency | 1.409 | <0.001 |  |
|  | Time | 0.03 | 0.021 |  |
| 2 | Intercept | -0.894 | <0.001 | 2198.01 |
|  | Medium level - YLDs caused by iron deficiency | 1.198 | <0.001 |  |
|  | High level risk - YLDs caused by iron deficiency | 1.421 | <0.001 |  |
|  | Time | 0.03 | 0.021 |  |
| 3 | Intercept | 0.53 | 0.001 | 2201.375 |
|  | Medium level - Government effectiveness index | -0.248 | 0.186 |  |
|  | High level - Government effectiveness index | -1.424 | <0.001 |  |
|  | Time | 0.03 | 0.02 |  |
| 4 | Intercept | -0.829 | <0.001 | 2203.632 |
|  | Medium level - Death rate of Vitamin A deficiency | 0.996 | <0.001 |  |
|  | High level - Death rate of Vitamin A deficiency | 1.431 | <0.001 |  |
|  | Time | 0.03 | 0.021 |  |
| 5 | Intercept | 0.449 | 0.01 | 2209.221 |
|  | Medium level - life expectancy | -0.15 | 0.443 |  |
|  | High level - life expectancy | -1.269 | <0.001 |  |
|  | Time | 0.03 | 0.021 |  |
| 6 | Intercept | 0.535 | 0.003 | 2210.642 |
|  | Medium level - Number of nurses per 1000 inhabitants | -0.348 | 0.08 |  |
|  | High level - Number of nurses per 1000 inhabitants | -1.315 | <0.001 |  |
|  | Time | 0.029 | 0.022 |  |
| 7 | Intercept | -0.668 | <0.001 | 2213.373 |
|  | Low middle income countries | 1.106 | <0.001 |  |
|  | Upper middle income countries | 1.089 | <0.001 |  |
|  | Time | 0.03 | 0.021 |  |
| 8 | Intercept | 0.107 | 0.472 | 2218.58 |
|  | Medium level - % population fully vaccinated | -0.157 | 0.101 |  |
|  | High level - % population fully vaccinated | -0.604 | <0.001 |  |
|  | Time | 0.048 | <0.001 |  |
| 9 | Intercept | -0.344 | 0.028 | 2222.302 |
|  | Medium level – Average weekly stringency index | 0.395 | <0.001 |  |
|  | High level – Average weekly stringency index | 0.547 | <0.001 |  |
|  | Time | 0.033 | 0.01 |  |
| 10 | Intercept | -0.688 | <0.001 | 2221.361 |
|  | Medium level - Death rate of diabetes | 0.852 | <0.001 |  |
|  | High level - Death rate of diabetes | 1.141 | <0.001 |  |
|  | Time | 0.03 | 0.02 |  |
| 11 | Intercept | 0.534 | 0.004 | 2222.302 |
|  | Medium level - GHS score | -0.504 | 0.018 |  |
|  | High level - GHS score | -1.152 | <0.001 |  |
|  | Time | 0.03 | 0.021 |  |
| 12 | Intercept | -0.658 | <0.001 | 2225.385 |
|  | Medium level - Death rate of cardiovascular diseases | 0.95 | <0.001 |  |
|  | High level - Death rate of cardiovascular diseases | 0.96 | <0.001 |  |
|  | Time | 0.03 | 0.02 |  |
| 13 | Intercept | 0.348 | 0.063 | 2227.431 |
|  | Medium level - YLDs caused by low bone density | -0.134 | 0.535 |  |
|  | High level - YLDs caused by low bone density | -0.971 | <0.001 |  |
|  | Time | 0.03 | 0.02 |  |
| 14 | Intercept | -0.594 | 0.002 | 2229.122 |
|  | Medium level - Death rate of chronic kidney diseases | 0.751 | 0.001 |  |
|  | High level - Death rate of chronic kidney diseases | 0.971 | <0.001 |  |
|  | Time | 0.03 | 0.02 |  |
| 15 | Intercept | 0.366 | 0.053 | 2229.43 |
|  | Medium level - population aged 65 older | -0.204 | 0.348 |  |
|  | High level - population aged 65 older | -0.957 | <0.001 |  |
|  | Time | 0.03 | 0.02 |  |
| 16 | Intercept | 0.488 | 0.009 | 2229.67 |
|  | Medium level - hospital beds per 1000 inhabitants | -0.536 | 0.016 |  |
|  | High level - hospital beds per 1000 inhabitants | -0.988 | <0.001 |  |
|  | Time | 0.03 | 0.02 |  |
| 17 | Intercept | 0.14 | 0.459 | 2232.396 |
|  | Medium level - Health expenditure | 0.201 | 0.364 |  |
|  | High level - Health expenditure | -0.684 | 0.003 |  |
|  | Time | 0.03 | 0.02 |  |
| 18 | Intercept | 0.153 | 0.422 | 2233.754 |
|  | Americas | 0.307 | 0.218 |  |
|  | Asia | 0.149 | 0.584 |  |
|  | Europe | -0.776 | 0.001 |  |
|  | Time | 0.03 | 0.021 |  |
| 19 | Intercept | 0.346 | 0.084 | 2241.269 |
|  | Medium level - Death rate of cancers | -0.484 | 0.039 |  |
|  | High level - Death rate of cancers | -0.599 | 0.011 |  |
|  | Time | 0.03 | 0.021 |  |
| 20 | Intercept | -0.202 | 0.307 | 2243.018 |
|  | Medium level - Death rate of chronic respiratory diseases | 0.057 | 0.805 |  |
|  | High level - Death rate of chronic respiratory diseases | 0.505 | 0.032 |  |
|  | Time | 0.03 | 0.022 |  |

Table S2 presents the mLME which includes the significant covariates selected from the forward selection during Delta variant dominant period.

Table S2. The final model of mLME in the Delta variant dominant period

| **Covariates** | **Coefficient** | **Standard error** | ***P* values** | **t value** | **Lower bound** | **Upper bound** |
| --- | --- | --- | --- | --- | --- | --- |
| Intercept | 0.298 | 0.253 | 0.241 | 1.177 | -0.203 | 0.799 |
| Low level-Government Effectiveness Index (T1) | - | - | - | - | - | - |
| Medium level-Government Effectiveness Index (T2) | -0.143 | 0.181 | .433 | -0.788 | -0.502 | 0.217 |
| High level-Government Effectiveness Index (T3) | -0.762 | 0.240 | 0.002 | -3.181 | -1.238 | -0.287 |
| Low level-% population fully vaccinated (T1) | - | - | - | - | - | - |
| Medium level-% population fully vaccinated (T2) | -0.059 | 0.096 | 0.538 | -0.617 | -0.248 | 0.129 |
| High level-% population fully vaccinated (T3) | -0.385 | 0.125 | 0.002 | -3.092 | -0.629 | -0.141 |
| Americas | - | - | - | - | - | - |
| Europe | -0.655 | 0.226 | 0.005 | -2.905 | -1.103 | -0.208 |
| Africa | -0.697 | 0.229 | 0.003 | -3.049 | -1.151 | -0.244 |
| Asia | -0.522 | 0.259 | 0.047 | -2.010 | -1.038 | -0.007 |
| Low level- Death rate Cardiovascular diseases (T1) | - | - | - | - | - | - |
| Medium level- Death rate Cardiovascular diseases (T2) | 0.517 | 0.21 | 0.015 | 2.472 | 0.102 | 0.932 |
| High level- Death rate Cardiovascular diseases (T3) | 0.445 | 0.24 | 0.064 | 1.875 | -0.026 | 0.915 |
| Low level - Stringency Index (T1) | - | - | - | - | - | - |
| Medium level- Stringency Index (T2) | 0.278 | 0.08 | 0.001 | 3.446 | 0.12 | 0.436 |
| High level- Stringency Index (T3) | 0.379 | 0.10 | 0 | 3.762 | 0.181 | 0.576 |
| Time | 0.044 | 0.01 | 0.001 | 3.444 | 0.019 | 0.07 |
| BIC | 2233.232 | | | | | |
| R2 Conditional | 0.823 | | | | | |
| R2 Marginal | 0.370 | | | | | |

*2.1.2. Testing the assumption of the mLME during Delta variant dominant period*

- Normality in the distribution of the residuals

To examine the normality of residual distribution, we plotted the residuals across all countries and over time in figure S1; and the residuals in the intercepts and slopes across countries and the between-country intercept and slope residuals during the Delta variant dominant period in figure S2.

Figure S1. The residuals across all countries and over the time for the mLME during Delta variant dominant period


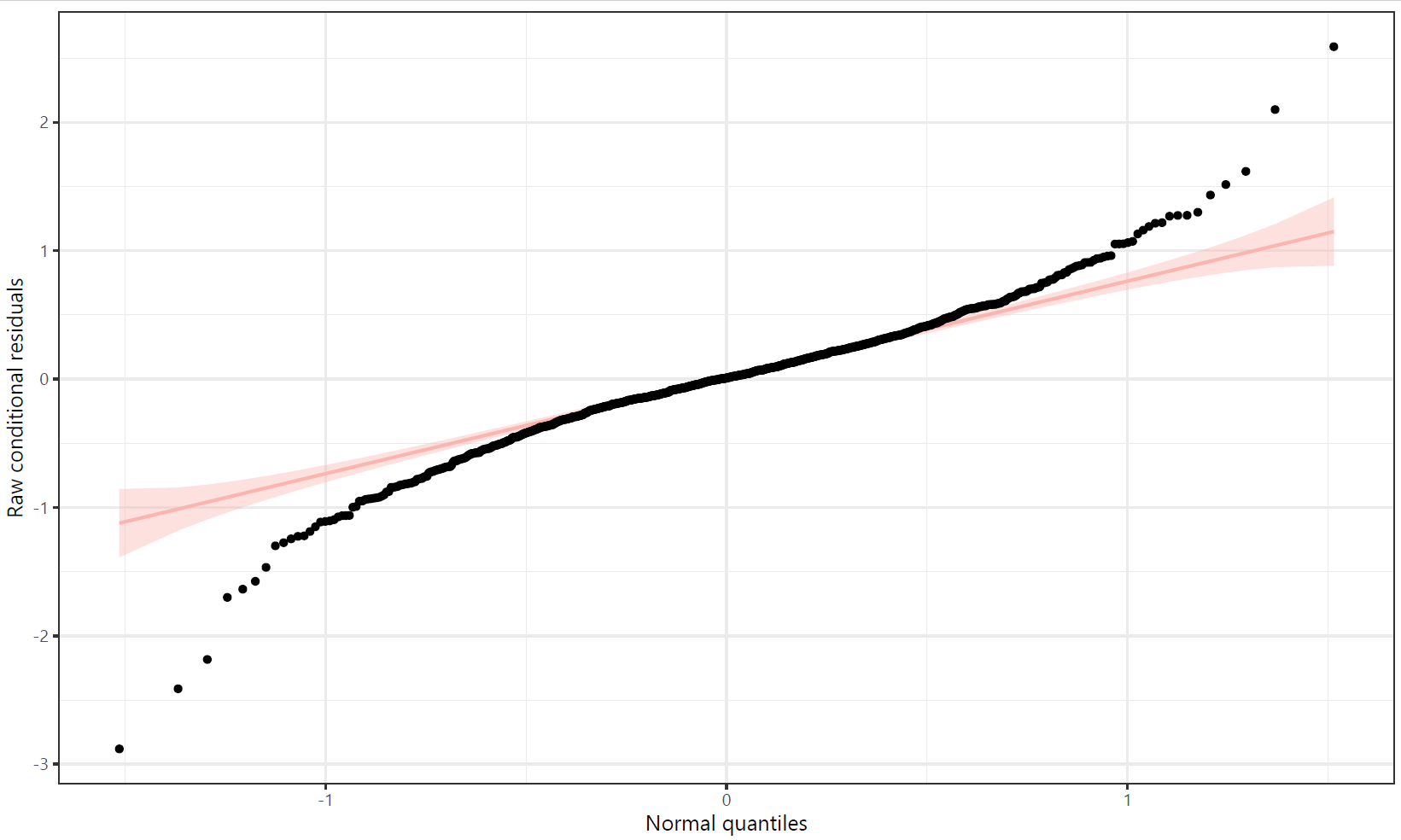


Figure S2. The residuals in the intercepts and slopes across countries and in the between-country intercept and slope residuals for mLME during Delta variant dominant period.


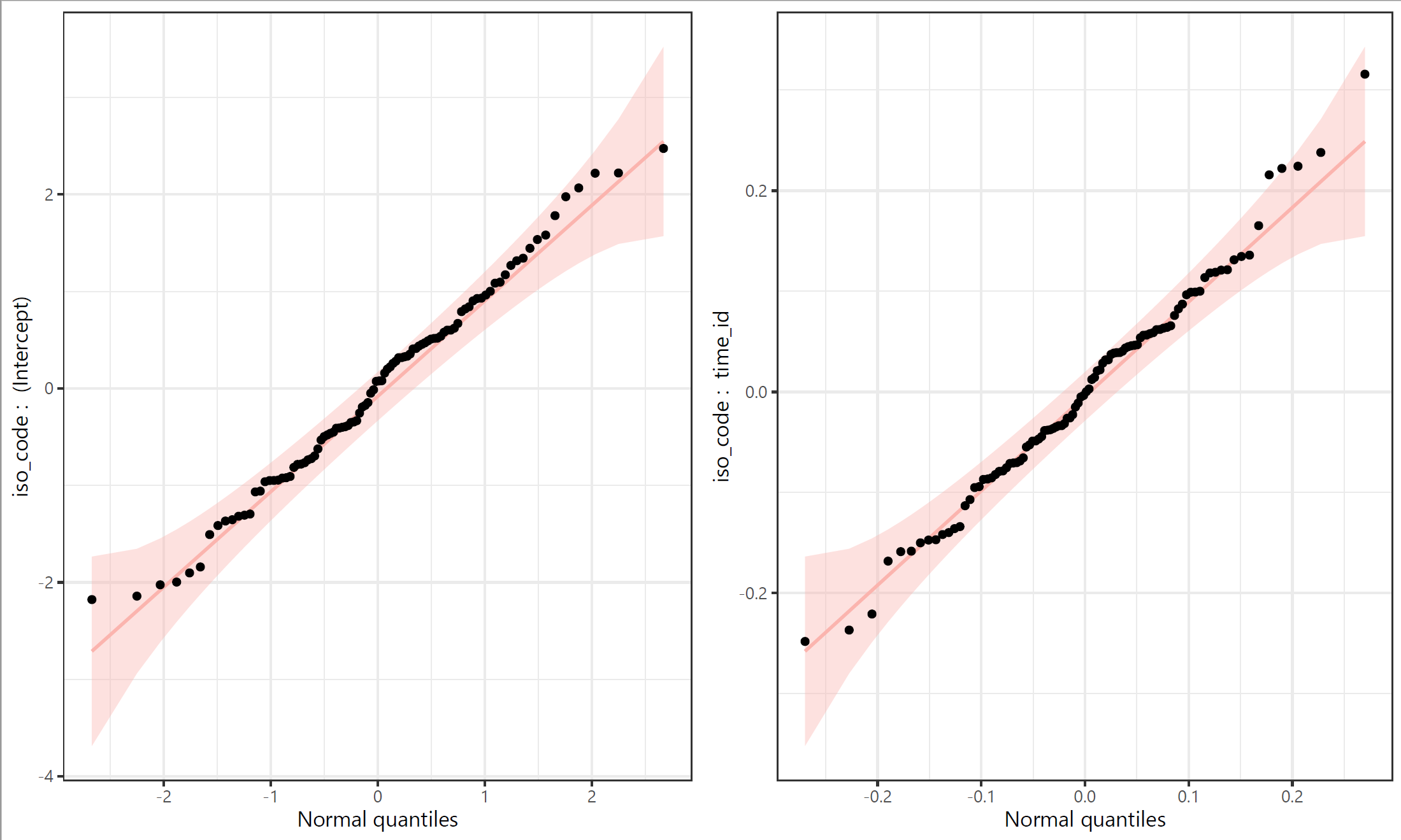


Based on results in the figure S1 and figure S2, we can conclude that the distribution of these residuals can be considered approximately normal.

2.2. Omicron variant dominant period

*2.2.1. Model selection of the multivariable linear mixed-effects model (mLME) during Omicron variant dominant period*

Table S3 shows the significant covariates (p<0.05) was identified using the univariate analysis of mLME. The variables were ranked in decreasing order of good of fitness based on their BIC values.

Table S3. Univariate model results and sequence in which covariates were added in the forward selection mLME during Omicron variant dominant period

| **Rank** | **Covariates** | **Coefficient** | ***P* value** | **BIC** |
| --- | --- | --- | --- | --- |
| 1 | Intercept | -2.28 | <0.001 | 2928.055 |
|  | Low middle income countries | 1.298 | <0.001 |  |
|  | Upper middle income countries | 1.081 | <0.001 |  |
|  | Time | 0.118 | <0.001 |  |
| 2 | Intercept | -0.961 | <0.001 | 2928.773 |
|  | Medium level - Government effectiveness index | -0.362 | 0.065 |  |
|  | High level - Government effectiveness index | -1.386 | <0.001 |  |
|  | Time | 0.118 | <0.001 |  |
| 3 | Intercept | -1.077 | <.001 | 2930.129 |
|  | Medium level - life expectancy | -0.119 | 0.545 |  |
|  | High level - life expectancy | -1.291 | <0.001 |  |
|  | Time | 0.118 | <0.001 |  |
| 4 | Intercept | -2.226 | <0.001 | 2944.038 |
|  | Medium level - YLDs caused by iron deficiency | 1.01 | <0.001 |  |
|  | High level risk - YLDs caused by iron deficiency | 1.067 | <0.001 |  |
|  | Time | 0.117 | <0.001 |  |
| 5 | Intercept | -0.974 | <0.001 | 2944.064 |
|  | Medium level - Number of nurses per 1000 inhabitants | -0.489 | 0.021 |  |
|  | High level - Number of nurses per 1000 inhabitants | -1.198 | <0.001 |  |
|  | Time | 0.117 | <0.001 |  |
| 6 | Intercept | -0.979 | <0.001 | 2944.417 |
|  | Medium level - Number of physicians per 1000 inhabitants | -0.475 | 0.025 |  |
|  | High level - Number of physicians per 1000 inhabitants | -1.191 | <0.001 |  |
|  | Time | 0.117 | <0.001 |  |
| 7 | Intercept | -1.125 | <0.001 | 2945.213 |
|  | Medium level - % population fully vaccinated | -0.503 | 0.001 |  |
|  | High level - % population fully vaccinated | -1.031 | <0.001 |  |
|  | Time | 0.128 | <0.001 |  |
| 8 | Intercept | -2.142 | <0.001 | 2945.976 |
|  | Medium level - Death rate of diabetes | 0.661 | 0.002 |  |
|  | High level - Death rate of diabetes | 1.155 | <.001 |  |
|  | Time | 0.117 | <0.001 |  |
| 9 | Intercept | -0.989 | <0.001 | 2949.329 |
|  | Medium level - GHS score | -0.542 | 0.013 |  |
|  | High level - GHS score | -1.101 | <0.001 |  |
|  | Time | 0.117 | <0.001 |  |
| 10 | Intercept | -2.128 | <0.001 | 2952.245 |
|  | Medium level - Death rate of cardiovascular diseases | 0.898 | <0.001 |  |
|  | High level - Death rate of cardiovascular diseases | 0.88 | <0.001 |  |
|  | Time | 0.117 | <.001 |  |
| 11 | Intercept | -2.109 | <.001 | 2952.55 |
|  | Medium level - Death rate of chronic kidney diseases | 0.709 | 0.001 |  |
|  | High level - Death rate of chronic kidney diseases | 1.004 | <0.001 |  |
|  | Time | 0.117 | <0.001 |  |
| 12 | Intercept | -1.309 | <0.001 | 2959.569 |
|  | Medium level - YLDs caused by low bone density | 0.023 | 0.918 |  |
|  | High level - YLDs caused by low bone density | -0.711 | 0.002 |  |
|  | Time | 0.117 | <0.001 |  |
| 13 | Intercept | -1.127 | <0.001 | 2959.855 |
|  | Medium level - hospital beds per 1000 inhabitants | -0.406 | 0.077 |  |
|  | High level - hospital beds per 1000 inhabitants | -0.824 | <0.001 |  |
|  | Time | 0.117 | <0.001 |  |
| 14 | Intercept | -1.247 | <0.001 | 2960.199 |
|  | Medium level - population aged 65 older | -0.125 | 0.581 |  |
|  | High level - population aged 65 older | -0.761 | 0.001 |  |
|  | Time | 0.118 | <0.001 |  |
| 15 | Intercept | -1.481 | <0.001 | 2960.321 |
|  | Medium level - % population received booster doses | -0.369 | 0.001 |  |
|  | High level - % population received booster doses | -0.633 | <0.001 |  |
|  | Time | 0.137 | <0.001 |  |
| 16 | Intercept | -1.952 | <0.001 | 2962.646 |
|  | Medium level - YLDs caused by metabolism disorders | 0.445 | 0.055 |  |
|  | High level - YLDs caused by metabolism disorders | 0.749 | 0.001 |  |
|  | Time | 0.118 | <0.001 |  |
| 17 | Intercept | -1.74 | <0.001 | 2964.25 |
|  | Medium level - Death rate of chronic respiratory diseases | -0.002 | 0.992 |  |
|  | High level - Death rate of chronic respiratory diseases | 0.577 | 0.012 |  |
|  | Time | 0.118 | <0.001 |  |
| 18 | Intercept | -1.365 | <0.001 | 2964.669 |
|  | Medium level - Health expenditure | 0.025 | 0.915 |  |
|  | High level - Health expenditure | -0.551 | 0.018 |  |
|  | Time | 0.117 | <0.001 |  |
| 19 | Intercept | -1.267 | <0.001 | 2966.24 |
|  | Americas | 0.112 | 0.659 |  |
|  | Asia | -0.451 | 0.109 |  |
|  | Europe | -0.771 | 0.001 |  |
|  | Time | 0.117 | <0.001 |  |
| 20 | Intercept | -1.713 | <0.001 | 2969.044 |
|  | Medium level – Average weekly stringency index | 0.105 | 0.256 |  |
|  | High level – Average weekly stringency index | 0.286 | 0.012 |  |
|  | Time | 0.124 | <0.001 |  |

Table S4 presents the results of the multivariable linear mixed-effects model (mLME) which includes the significant covariates selected from the forward selection during Omicron variant dominant period.

Table S4. The final model of multivariable linear mixed-effects model in Omicron variant dominant period

| **Covariates** | **Coefficient** | **Standard error** | ***P* value** | ***t* value** | **Lower bound** | **Upper bound** |
| --- | --- | --- | --- | --- | --- | --- |
| Intercept | -3.198 | 0.272 | <0.001 | -11.751 | -3.737 | -2.659 |
| High income group | - | - | - | - | - | - |
| Low middle income group | 1.788 | 0.246 | <0.001 | 7.267 | 1.301 | 2.276 |
| Upper middle income group | 1.055 | 0.209 | <0.001 | 5.040 | 0.64 | 1.47 |
| Low level-% population aged 65 older(T1) | - | - | - | - | - | - |
| Medium level-% population aged 65 older(T2) | 0.331 | 0.195 | 0.093 | 1.693 | -0.057 | 0.719 |
| High level-% population aged 65 older(T3) | 0.737 | 0.252 | 0.004 | 2.921 | 0.237 | 1.238 |
| Low level-Booster dose coverage (T1) | - | - | - | - | - | - |
| Medium level-Booster dose coverage (T2) | -0.197 | 0.112 | 0.078 | -1.763 | -0.416 | 0.022 |
| High level-Booster dose coverage (T3) | -0.321 | 0.223 | 0.038 | -2.083 | -0.624 | -0.018 |
| Low level- YLDs caused by metabolism disorders (T1) | - | - | - | - | - | - |
| Medium level- YLDs caused by metabolism disorders (T2) | 0.441 | 0.206 | 0.012 | 2.543 | 0.097 | 0.785 |
| High level- YLDs caused by metabolism disorders (T3) | 0.843 | 0.265 | <0.001 | 4.677 | 0.486 | 1.2 |
| Time | 0.127 | 0.06 | <0.001 | 7.795 | 0.095 | 0.16 |
| BIC | 2952.792 | | | | | |
| R2 Conditional | 0.744 | | | | | |
| R2 Marginal | 0.346 | | | | | |

*2.2.2. Testing the assumption of the mLME during Omicron variant dominant period*

- Normality in the distribution of the residuals

The figure S3 presents the residuals across all countries and over the time.

The residuals in the intercepts and slopes across countries and in the between-country intercept and slope residuals were indicated in the Figure S4.

According to results of Figure S3 and Figure S4, the distribution of these residuals can be considered approximately normal.

Figure S3. The residuals across all countries and over the time for the mLME during Omicron variant dominant period.


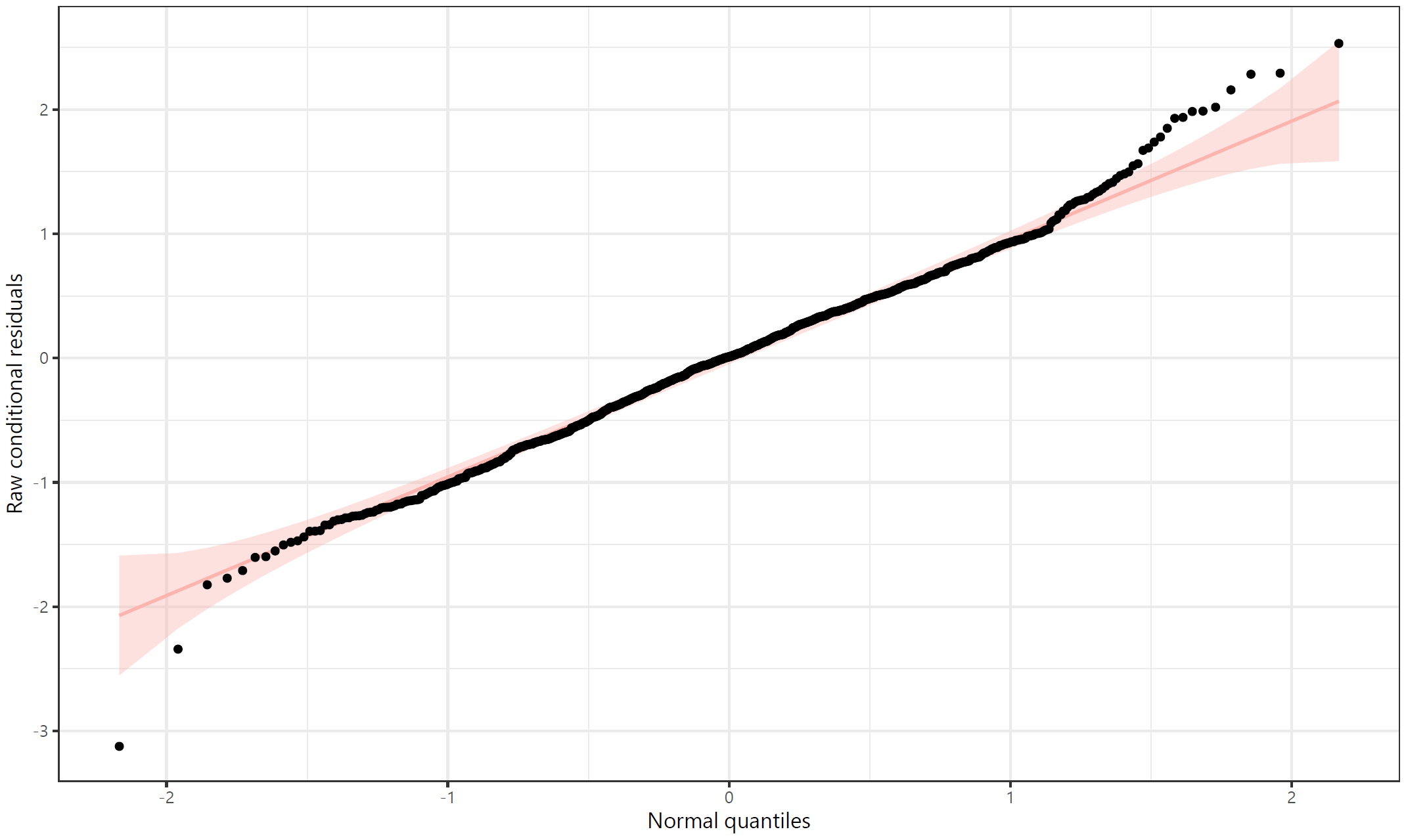


Figure S4. The residuals in the intercepts and slopes across countries and in the between-country intercept and slope residuals for mLME during Omicron variant dominant period.


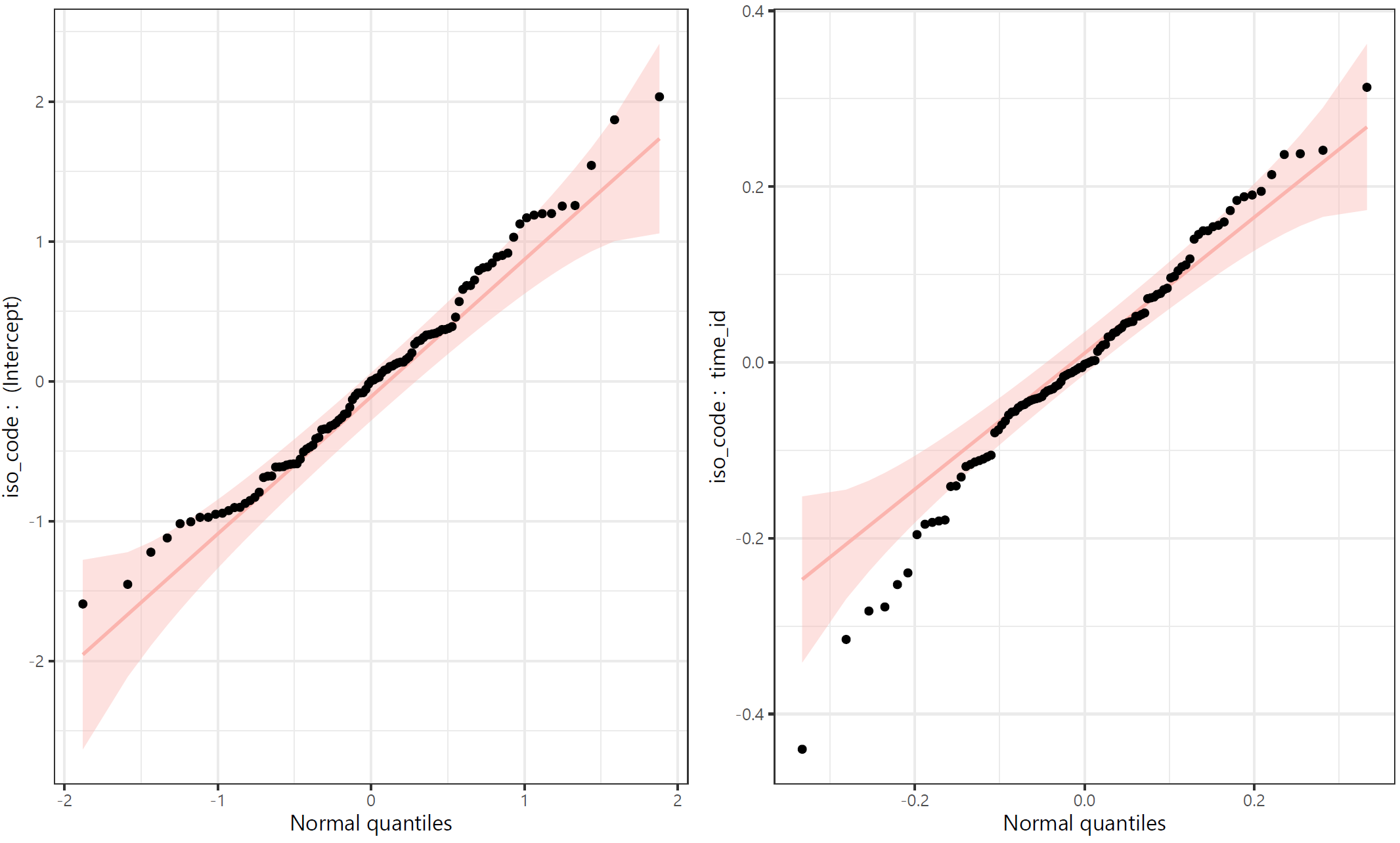


Table S5. Univariate linear regression model of factors associated with the increase in AWIFR between Delta and Omicron periods (N=99)

| **Covariates** | **β** | **SE** | **95% CI** | ***P* value** |
| --- | --- | --- | --- | --- |
| GDP per capita | -0.632 | 0.122 | -0.874 - (-0.391) | <0.001 |
| Life expectancy | -0.512 | 0.124 | -0.758 - (-0.266) | <0.001 |
| Median age | -0.509 | 0.131 | -0.768 - (-0.250) | <0.001 |
| % population aged over 65 | -0.533 | 0.125 | -0.780 - (-0.285) | <0.001 |
| Government effectiveness index | -0.666 | 0.120 | -0.904 - (-0.427) | <0.001 |
| Rule of law index | -0.621 | 0.122 | -0.863 - (-0.379) | <0.001 |
| GHS index | -0.385 | 0.133 | -0.649 –(-0.120) | 0.005 |
| GHSI1 | -0.488 | 0.129 | -0.745 - (-0.231) | <0.001 |
| GHSI3 | -0.338 | 0.135 | -0.606 - (-0.069) | 0.014 |
| GHSI4 | -0.377 | 0.133 | -0.641 - (-0.113) | 0.006 |
| GHSI5 |  |  |  |  |
| Low level | ref. |  |  |  |
| Medium level | -0.267 | 0.134 | -0.533 - (-0.002) | 0.048 |
| High level | -0.287 | 0.137 | -0.561 - (-0.013) | 0.039 |
| GHSI6 | -0.575 | 0.122 | -0.816 - (-0.333) | <0.001 |
| HAQ index | -0.618 | 0.117 | -0.851 - (0.384) | <0.001 |
| No. Physicians per 1000 | -0.555 | 0.126 | -0.806 - (-0.306) | <0.001 |
| No. Nurses and midwives (per 1,000 people) | -0.658 | 0.120 | -0.897 - (-0.419) | <0.001 |
| % GDP for health expenditure | -0.291 | 0.132 | -0.553 - (-0.029) | 0.030 |
| Hospital beds (per 1000 people) |  |  |  |  |
| Low level | ref. |  |  |  |
| Medium level | -0.427 | 0.124 | -0.674 - (-0.180) | 0.001 |
| High level | -0.606 | 0.124 | -0.852 - (-0.359) | <0.001 |
| Country income |  |  |  |  |
| Low income | ref. |  |  |  |
| Upper middle income | 0.483 | 0.129 | 0.228 - 0.739 | <0.001 |
| High income | 0.451 | 0.122 | 0.208 - 0.694 | <0.001 |
| % population vaccinated with a booster dose | -0.273 | 0.137 | -0.544 - (-0.001) | 0.049 |
| The increase in % population vaccinated with a booster dose | -0.297 | 0.135 | -0.565 - (-0.029) | 0.029 |
| The increase in average stringency index | -0.424 | 0.133 | -0.687 - (-0.160) | 0.002 |
| Death rate caused by diabetes diseases |  |  |  |  |
| Low level | ref. |  |  |  |
| Medium level | 0.484 | 0.121 | 0.245 - 0.723 | <0.001 |
| High level | 0.665 | 0.121 | 0.426 - 0.904 | <0.001 |
| Death rate caused by chronic kidney disease |  |  |  |  |
| Low level | ref. |  |  |  |
| Medium level | 0.474 | 0.126 | 0.224 - 0.724 | <0.001 |
| High level | 0.537 | 0.126 | 0.287 - 0.787 | <0.001 |
| Death rate caused by non-communicable diseases |  |  |  |  |
| Low level | ref. |  |  |  |
| Medium level | 0.521 | 0.128 | 0.2686 - 0.775 | <0.001 |
| High level | 0.411 | 0.128 | 0.158 - 0.665 | 0.002 |
| Death rate caused by cardiovascular disease |  |  |  |  |
| Low level | ref. |  |  |  |
| Medium level | 0.376 | 0.133 | 0.111 - 0.641 | 0.006 |
| High level | 0.308 | 0.133 | 0.043 - 0.573 | 0.023 |
| Death rate caused by diabetes and kidney diseases |  |  |  |  |
| Low level | ref. |  |  |  |
| Medium level | 0.386 | 0.119 | 0.149 - 0.622 | 0.002 |
| High level | 0.707 | 0.119 | 0.469 - 0.944 | <0.001 |
| Death rate caused by cancer disease | -0.375 | 0.134 | -0.641 - (-0.108) | 0.006 |
| Death rate caused by iron deficiency |  |  |  |  |
| Low level | ref. |  |  |  |
| Medium level | 0.553 | 0.116 | 0.322 - 0.783 | <0.001 |
| High level | 0.726 | 0.116 | 0.496 - 0.957 | <0.001 |
| HAQ index | -0.618 | 0.117 | -0.851 - (0.384) | <0.001 |
| Death rate caused by vitamin A deficiency |  |  |  |  |
| Low level | ref. |  |  |  |
| Medium level | 0.388 | 0.119 | 0.152 - 0.625 | 0.002 |
| High level | 0.709 | 0.119 | 0.473 - 0.946 | <0.001 |
| Death rate caused by zinc deficiency |  |  |  |  |
| Low level | ref. |  |  |  |
| Medium level | 0.518 | 0.121 | 0.278 - 0.757 | <0.001 |
| High level | 0.649 | 0.121 | 0.409 - 0.888 | <0.001 |
| YLDs caused by low bone density | -0.538 | 0.125 | -0.786 - (-0.290) | <0.001 |
| Death rate caused by PM.25 |  |  |  |  |
| Low level | ref. |  |  |  |
| Medium level | 0.530 | 0.126 | 0.280 - 0.780 | <0.001 |
| High level | 0.486 | 0.126 | 0.236 - 0.736 | <0.001 |

Table S6. Multivariable linear regression model of factors associated with

the increase in AWIFR between Delta and Omicron periods (N=99).

| **Covariates** | **β** | **SE** | **95% CI** | ***P* value** |
| --- | --- | --- | --- | --- |
| Government effectiveness index |  |  |  |  |
| Low level | ref. |  |  |  |
| Medium level | -0.121 | 0.123 | -0.366 -0.123 | 0.326 |
| High level | -0.438 | 0.157 | -0.750 – (-0.126) | 0.006 |
| Death rates caused by diabetes and kidneys |  |  |  |  |
| Low level | ref. |  |  |  |
| Medium level | 0.118 | 0.164 | -0.207 – 0.443 | 0.472 |
| High level | 0.472 | 0.193 | 0.089 – 0.855 | 0.016 |
| % population aged over 65 |  |  |  |  |
| Low level | ref. |  |  |  |
| Medium level | 0.259 | 0.129 | 0.001 – 0.516 | 0.049 |
| High level | 0.167 | 0.198 | -0.227 – 0.559 | 0.402 |
| Average stringency index |  |  |  |  |
| Low level | ref. |  |  |  |
| Medium level | -0.115 | 0.122 | -0.357 – 0.127 | 0.347 |
| High level | -0.247 | 0.121 | -0.487 – (-0.007) | 0.044 |
| Adjusted $R^{2}$ | 38.56 |  |  |  |

Table S7. Univariate linear regression model of factors associated with

the average weekly infection rate during the Delta period (N = 102).

| **Covariates** | **β** | **SE** | **95% CI** | | ***P* value** |
| --- | --- | --- | --- | --- | --- |
|  |  |  | **Lower bound** | **Upper bound** |  |
| Life expectancy | 0.06 | 0.026 | 0.010 | 0.111 | 0.020 |
| % population aged over 65 | -0.059 | 0.012 | -0.084 | -0.035 | <0.001 |
| Government Effectiveness | -0.499 | 0.076 | -0.650 | -0.347 | <0.001 |
| GHS index | 0.050 | 0.013 | 0.024 | 0.076 | <0.001 |
| HAQ index | 0.021 | 0.009 | 0.004 | 0.038 | 0.015 |
| UHC index | 0.042 | 0.013 | 0.016 | 0.067 | 0.002 |
| HAQ index | 0.021 | 0.009 | 0.004 | 0.038 | 0.015 |
| No. Physicians per 1000 | 0.249 | 0.116 | 0.018 | 0.479 | 0.035 |
| % GDP for health expenditure | 0.176 | 0.072 | 0.033 | 0.319 | 0.016 |
| % population fully vaccinated | -0.011 | 0.004 | -0.019 | -0.004 | 0.003 |
| Average stringency index | 0.058 | 0.014 | 0.031 | 0.085 | <0.001 |
| Low middle income | 1.932 | 0.750 | 0.443 | 3.420 | 0.012 |
| Upper middle income | 3.150 | 0.741 | 1.679 | 4.621 | <0.001 |
| High income | 1.902 | 0.716 | 0.481 | 3.323 | 0.009 |
| Africa | 3.358 | 1.339 | 0.699 | 6.016 | 0.014 |
| American | 2.040 | 1.008 | 0.038 | 4.041 | 0.046 |
| Asia | 2.503 | 1.209 | 0.665 | 3.168 | 0.023 |
| Europe | 2.334 | 1.028 | 0.647 | 4.180 | 0.027 |
| YLDs caused by vitamin A deficiency | -0.048 | 0.012 | -0.072 | -0.025 | <0.001 |
| YLDs caused by iron deficiency | -0.796 | 0.190 | -1.172 | -0.420 | <0.001 |
| YLDs caused by zinc deficiency | -18.16 | 6.076 | -30.215 | -6.106 | 0.004 |
| YLDs caused by tobacco use | 0.003 | 0.001 | 0.001 | 0.005 | 0.012 |
| YLDs caused by smoking | 0.003 | 0.001 | 0.001 | 0.005 | 0.012 |
| YLDs caused by low bone density | -0.191 | 0.078 | -0.347 | -0.036 | 0.017 |
| YLDs caused by PM2.5 | -0.007 | 0.003 | -0.013 | -0.001 | 0.017 |
| Death rates caused by smoking | 0.012 | 0.005 | 0.002 | 0.022 | 0.024 |
| Death rates caused by diabetes and kidneys | 0.006 | 0.002 | 0.003 | 0.009 | <0.001 |

Table S8. Multivariable linear regression model of factors associated with

the average weekly infection rate during the Delta period (N=102).

| **Covariates** | **β** | **SE** | **95% CI** | ***P* value** |
| --- | --- | --- | --- | --- |
| Government effectiveness index |  |  |  |  |
| Low level | ref. |  |  |  |
| Medium level | 0.827 | 0.478 | -0.122 - 1.776 | 0.087 |
| High level | -0.234 | 0.568 | -1.363 - 0.894 | 0.681 |
| Death rates caused by diabetes and kidneys |  |  |  |  |
| Low level | ref. |  |  |  |
| Medium level | -0.006 | 0.560 | -1.19 - 1.107 | 0.912 |
| High level | -0.254 | 0.667 | -1.579 - 1.070 | 0.703 |
| Region |  |  |  |  |
| Africa | ref. |  |  |  |
| American | -0.015 | 0.611 | -1.229 - 1.119 | 0981 |
| Asia | 0.406 | 0.609 | -0.805 - 1.617 | 0.507 |
| Europe | -0.779 | 0.545 | -1.862 - 0.304 | 0.165 |
| % population fully vaccinated |  |  |  |  |
| Low level | ref. |  |  |  |
| Medium level | 0.213 | 0.475 | -0.729 - 1.157 | 0.654 |
| High level | 0.542 | 0.468 | -0.387 - 1.471 | 0.249 |
| Adjusted $R^{2}$ | 14.11 |  |  |  |

Table S9. Univariable linear regression model of factors associated with

the average weekly infection rate during the Omicron period (N = 107).

| **Covariates** | **β** | **SE** | **95% CI** | | ***P* value** |
| --- | --- | --- | --- | --- | --- |
|  |  |  | **Lower bound** | **Upper bound** |  |
| Life expectancy | 0.202 | 0.020 | 0.161 | 0.243 | <0.001 |
| Median age | 0.152 | 0.018 | 0.116 | 0.188 | <0.001 |
| % population aged over 65 | 0.214 | 0.027 | 0.161 | 0.266 | <0.001 |
| % Urban population | 0.054 | 0.009 | 0.037 | 0.071 | <0.001 |
| UHC index | 0.113 | 0.010 | 0.093 | 0.132 | <0.001 |
| GHS index | 0.131 | 0.010 | 0.111 | 0.150 | <0.001 |
| HAQ index | 0.071 | 0.007 | 0.058 | 0.085 | <0.001 |
| Physicians (per 1,000 people) | 0.824 | 0.108 | 0.611 | 1.037 | <0.001 |
| Nurses and midwives per 1,000 people | 0.257 | 0.040 | 0.178 | 0.337 | <0.001 |
| % GDP for health expenditure | 0.493 | 0.069 | 0.355 | 0.630 | <0.001 |
| Hospital beds per 1000 people | 0.429 | 0.102 | 0.226 | 0.632 | <0.001 |
| % population fully vaccinated | 0.043 | 0.010 | 0.023 | 0.062 | <0.001 |
| % population vaccinated  at least one dose | 0.038 | 0.009 | 0.020 | 0.056 | <0.001 |
| Average stringency index | 0.051 | 0.017 | 0.017 | 0.084 | 0.004 |
| Africa | 1.301 | 0.543 | 0.223 | 2.378 | 0.018 |
| Asia | 3.062 | 1.275 | 0.532 | 5.592 | 0.018 |
| Europe | -2.697 | 0.567 | -3.822 | -1.571 | <0.001 |
| Low middle income | 1.813 | 0.702 | 0.420 | 3.205 | 0.011 |
| Upper middle income | 3.901 | 0.705 | 2.503 | 5.299 | <0.001 |
| High income | 4.365 | 0.678 | 3.020 | 5.710 | <0.001 |
| YLDs caused by vitamin A deficiency | -0.089 | 0.010 | -0.108 | -0.07 | <0.001 |
| YLDs caused by low bone density | 0.023 | 0.005 | 0.014 | 0.032 | <0.001 |
| YLDs caused by zinc deficiency | -6.269 | 1.212 | -8.672 | -3.866 | <0.001 |
| YLDs caused by iron deficiency | -0.007 | 0.001 | -0.008 | -0.005 | <0.001 |
| YLDs caused by PM.25 | -0.010 | 0.001 | -0.013 | -0.008 | <0.001 |
| YLDs caused by tobacco use | 0.006 | 0.001 | 0.003 | 0.008 | <0.001 |
| Death rate caused by  non-communicable diseases | -0.006 | 0.001 | -0.009 | -0.004 | <0.001 |
| Death rate caused by chronic respiratory disease | -0.028 | 0.005 | -0.038 | -0.018 | <0.001 |
| Death rate caused by diabetes diseases | -0.037 | 0.007 | -0.051 | -0.023 | <0.001 |
| Death rate caused by cardiovascular diseases | -0.008 | 0.002 | -0.011 | -0.004 | <0.001 |
| Death rate caused by chronic kidney diseases | -0.061 | 0.014 | -0.088 | -0.034 | <0.001 |

Table S10. Multivariable linear regression model of factors associated with

the average weekly infection rate during the Omicron period (N=107).

| **Covariates** | **β** | **SE** | **95% CI** | ***P* value** |
| --- | --- | --- | --- | --- |
| % population vaccinated with a booster dose |  |  |  |  |
| Low level | ref. |  |  |  |
| Medium level | 1.009 | 0.403 | 0.209 - 1.809 | 0.014 |
| High level | 0.842 | 0.440 | -0.301 - 1.715 | 0.059 |
| YLDs caused by metabolic disoirders |  |  |  |  |
| Low level | ref. |  |  |  |
| Medium level | -0.069 | 0.399 | -0.826 - 0.724 | 0.863 |
| High level | -0.455 | 0.423 | -1.295 - 0.384 | 0.284 |
| % population aged over 65 |  |  |  |  |
| Low level | ref. |  |  |  |
| Medium level | 1.849 | 0.445 | 0.967 - 2.733 | <0.001 |
| High level | 2.626 | 0.569 | 1.497 - 3.756 | <0.001 |
| Income |  |  |  |  |
| Low middle income | ref. |  |  |  |
| Upper middle income | -0.775 | 0.589 | -1.943 - 0.393 | 0.191 |
| High income | 0.448 | 0.481 | -0.507 – 1.403 | 0.354 |
| Adjusted $R^{2}$ | 48.68 |  |  |  |

Table S11. Univariable linear regression model of factors associated with

the average weekly fatality rate during the Delta period (N = 102).

| **Covariates** | **β** | **SE** | **95% CI** | | ***P* value** |
| --- | --- | --- | --- | --- | --- |
|  |  |  | **Lower bound** | **Upper bound** |  |
| Life expectancy | 0.060 | 0.026 | 0.010 | 0.111 | 0.020 |
| Government effectiveness index | -0.500 | 0.076 | -0.650 | -0.369 | <0.001 |
| UHC index | 0.042 | 0.013 | 0.016 | 0.067 | 0.002 |
| GHS index | 0.050 | 0.013 | 0.024 | 0.076 | 0.001 |
| HAQ index | 0.021 | 0.009 | 0.004 | 0.038 | 0.015 |
| No. Physicians per 1,000 | 0.249 | 0.116 | 0.018 | 0.479 | 0.035 |
| % GDP for health expenditure | 0.176 | 0.072 | 0.033 | 0.319 | 0.016 |
| % population fully vaccinated | -0.011 | 0.004 | -0.019 | -0.005 | <0.001 |
| Average stringency index | 0.058 | 0.014 | 0.031 | 0.085 | <0.001 |
| Africa | 3.358 | 1.339 | 0.699 | 6.016 | 0.014 |
| American | 2.040 | 1.008 | 0.038 | 4.041 | 0.046 |
| Asia | 3.426 | 1.406 | 0.701 | 6.528 | 0.015 |
| Europe | 3.540 | 1.108 | 0708 | 6.654 | 0.042 |
| Low middle income | 1.932 | 0.750 | 0.443 | 3.420 | 0.012 |
| Upper middle income | 3.150 | 0.741 | 1.679 | 4.621 | 0 |
| High income | 1.902 | 0.716 | 0.481 | 3.323 | 0.009 |
| YLDs caused by vitamin A deficiency | -0.048 | 0.012 | -0.072 | -0.025 | <0.001 |
| YLDs caused by iron deficiency | -0.003 | 0.001 | -0.004 | -0.001 | 0.005 |
| YLDs caused by PM.25 | -0.007 | 0.003 | -0.013 | -0.001 | 0.017 |
| YLDs caused by tobacco | 0.003 | 0.001 | 0.001 | 0.005 | 0.012 |
| Death rate caused by cardiovascular diseases | 0.002 | 0.001 | 0.001 | 0.004 | 0.003 |
| Death rate caused by diabetes diseases | 0.012 | 0.003 | 0.005 | 0.018 | <0.001 |
| Death rate caused by chronic respiratory disease | 0.007 | 0.003 | 0.001 | 0.012 | 0.024 |

Table S12. Multivariable linear regression model of factors associated with

the average weekly fatality rate during the Delta period (N=102).

| **Covariates** | **β** | **SE** | **95% CI** | ***P* value** |
| --- | --- | --- | --- | --- |
| Government effectiveness index |  |  |  |  |
| Low level | ref. |  |  |  |
| Medium level | 0.343 | 0.264 | -0.181 - 0.868 | 0.197 |
| High level | -0.457 | 0.314 | -1.081 - 0.167 | 0.149 |
| Death rates caused by diabetes |  |  |  |  |
| Low level | ref. |  |  |  |
| Medium level | 0.149 | 0.309 | -0.466 - 0.764 | 0.632 |
| High level | -0.001 | 0.369 | -0.734 - 0.731 | 0.967 |
| Region |  |  |  |  |
| Africa | ref. |  |  |  |
| American | 0.254 | 0.338 | -0.417 - 0.925 | 0.454 |
| Asia | -0.123 | 0.337 | -0.793 - 0.546 | 0.716 |
| Europe | -0.489 | 0.301 | -1.088 - 1.096 | 0.108 |
| % population fully vaccinated |  |  |  |  |
| Low level | ref. |  |  |  |
| Medium level | -0.149 | 0.263 | -0.671 - 0.372 | 0.571 |
| High level | 0.283 | 0.259 | -0.230 - 0.797 | 0.276 |
| Adjusted $R^{2}$ | 5.71 |  |  |  |

Table S13. Univariable linear regression model of factors associated with

the average weekly fatality rate during the Omicron period (N = 107).

| **Covariates** | **β** | **SE** | **95% CI** | | ***P* value** |
| --- | --- | --- | --- | --- | --- |
|  |  |  | **Lower bound** | **Upper bound** |  |
| Life expectancy | 0.055 | 0.011 | 0.032 | 0.077 | <0.001 |
| Median age | 0.046 | 0.009 | 0.028 | 0.065 | <0.001 |
| % Urban population | 0.015 | 0.004 | 0.007 | 0.024 | <0.001 |
| % population aged above 65 | 0.066 | 0.013 | 0.040 | 0.093 | <0.001 |
| GHS index | 0.043 | 0.006 | 0.032 | 0.054 | <0.001 |
| UHC index | 0.032 | 0.006 | 0.020 | 0.043 | <0.001 |
| HAQ index | 0.019 | 0.004 | 0.011 | 0.027 | <0.001 |
| No. Physicians per 1,000 people | 0.209 | 0.056 | 0.098 | 0.319 | <0.001 |
| No. Nurses and midwives  per 1,000 people | 0.061 | 0.020 | 0.021 | 0.100 | 0.003 |
| Hospital beds (per 1000 people) | 0.123 | 0.047 | 0.030 | 0.217 | 0.010 |
| % GDP for health expenditure | 0.185 | 0.033 | 0.120 | 0.249 | <0.001 |
| Africa | 2.621 | 0.624 | 1.384 | 3.859 | <0.001 |
| American | -0.718 | 0.278 | -1.269 | -0.167 | 0.011 |
| Asia | 2.621 | 0.624 | 1.384 | 3.859 | <0.001 |
| Europe | -0.718 | 0.278 | -1.269 | -0.167 | 0.011 |
| Low middle income | 1.063 | 0.345 | 0.379 | 1.747 | 0.003 |
| Upper middle income | 1.306 | 0.358 | 0.595 | 2.016 | <0.001 |
| High income | 1.063 | 0.345 | 0.379 | 1.747 | 0.003 |
| % population fully vaccinated | 0.012 | 0.005 | 0.003 | 0.021 | 0.009 |
| % population vaccinated with  a booster dose | 0.009 | 0.004 | 0.006 | 0.017 | 0.038 |
| Average stringency index | 0.024 | 0.007 | 0.009 | 0.039 | 0.002 |
| YLDs caused by iron deficiency | -0.002 | 0.001 | -0.003 | -0.001 | <0.001 |
| YLDs caused by vitamin A deficiency | -0.023 | 0.005 | -0.034 | -0.013 | <0.001 |
| YLDs caused by zinc deficiency | -1.82 | 0.572 | -2.954 | -0.687 | 0.002 |
| YLDs caused by low bone density | 0.006 | 0.002 | 0.001 | 0.01 | 0.011 |
| YLDs caused by PM.25 | -0.003 | 0.001 | -0.004 | -0.002 | <0.001 |
| YLDs caused by tobacco | 0.002 | 0.001 | 0.001 | 0.003 | <0.001 |
| Death rate caused by non-communicable diseases | -0.002 | 0.001 | -0.003 | -0.001 | <0.001 |
| Death rate caused by metabolic disorders | -0.003 | 0.001 | -0.004 | -0.001 | <0.001 |
| Death rate caused by diabetes and kidney diseases | -0.008 | 0.002 | -0.013 | -0.004 | 0.001 |
| Death rate caused by diabetes diseases | -0.011 | 0.003 | -0.018 | -0.005 | 0.001 |
| Death rate caused by cardiovascular disease | -0.002 | 0.001 | -0.004 | -0.001 | 0.003 |
| Death rate caused by chronic kidney disease | -0.017 | 0.006 | -0.029 | -0.004 | 0.009 |

Table S14. Multivariable linear regression model of factors associated with

the average weekly fatality rate during the Omicron period (N=107).

| **Covariates** | **β** | **SE** | **95% CI** | ***P* value** |
| --- | --- | --- | --- | --- |
| % population vaccinated with a booster dose |  |  |  |  |
| Low level | ref. |  |  |  |
| Medium level | 0.461 | 0.207 | 0.051 - 0.872 | 0.028 |
| High level | 0.073 | 0.226 | -0.375 - 0.512 | 0.747 |
| YLDs caused by metabolic disoirders |  |  |  |  |
| Low level | ref. |  |  |  |
| Medium level | 0.069 | 0.205 | -0.338 - 0.478 | 0.738 |
| High level | -0.095 | 0.217 | -0.527 - 0.335 | 0.660 |
| Income |  |  |  |  |
| Low middle income | ref. |  |  |  |
| Upper middle income | 0.138 | 0.302 | -0.461 - 0.738 | 0.024 |
| High income | 0.567 | 0.247 | -0.077 - 1.058 | 0.648 |
| % population aged over 65 |  |  |  |  |
| Low level | ref. |  |  |  |
| Medium level | 0.779 | 0.228 | 0.327 - 1.233 | 0.001 |
| High level | 1.217 | 0.292 | 0.637 - 1.797 | <0.001 |
| Adjusted $R^{2}$ | 30.39 |  |  |  |

Figure S5. World map of the COVID-19 crude mortality rate among countries During Delta period.

**
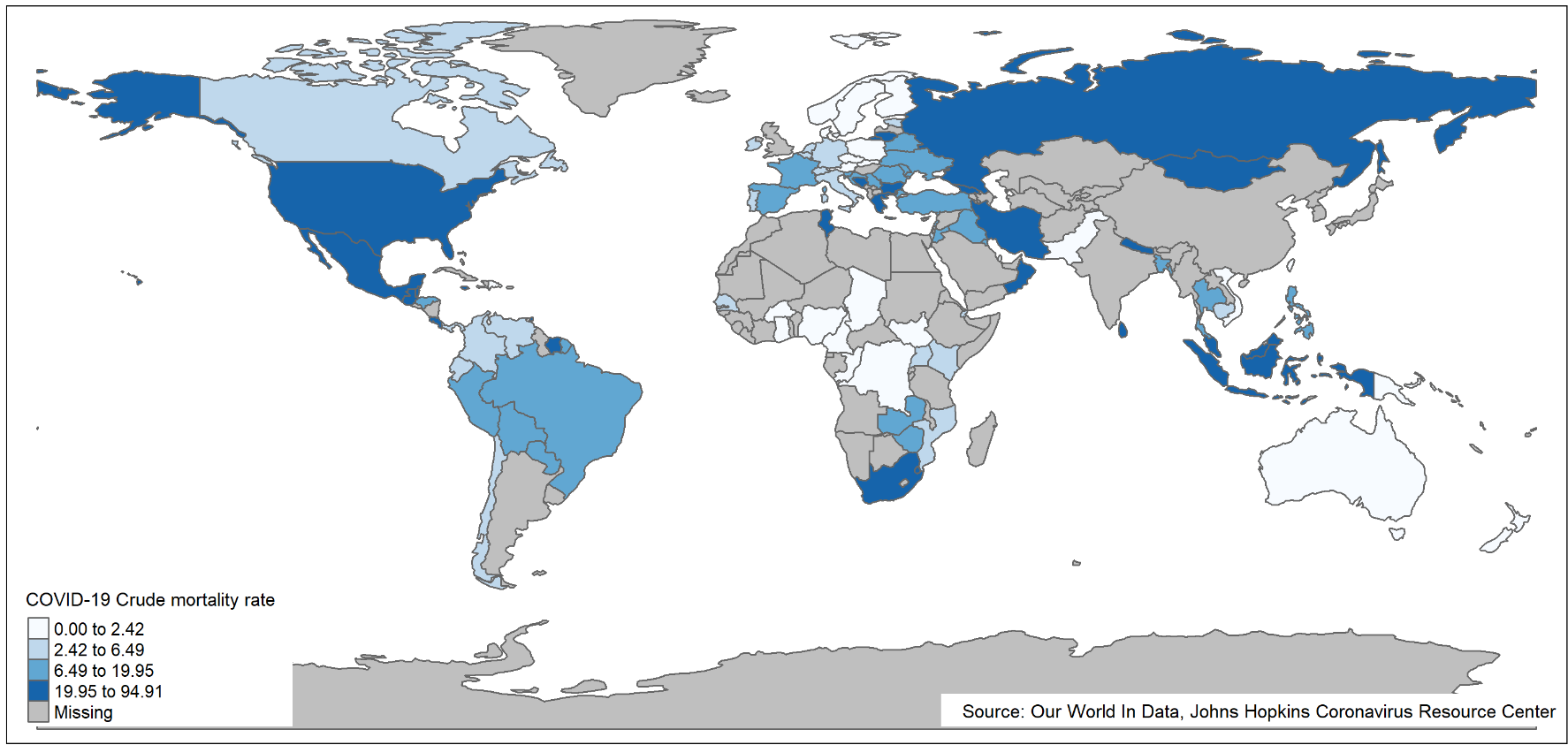
**

Figure S6. World map of the COVID-19 crude mortality rate among countries during Omicron period.

**
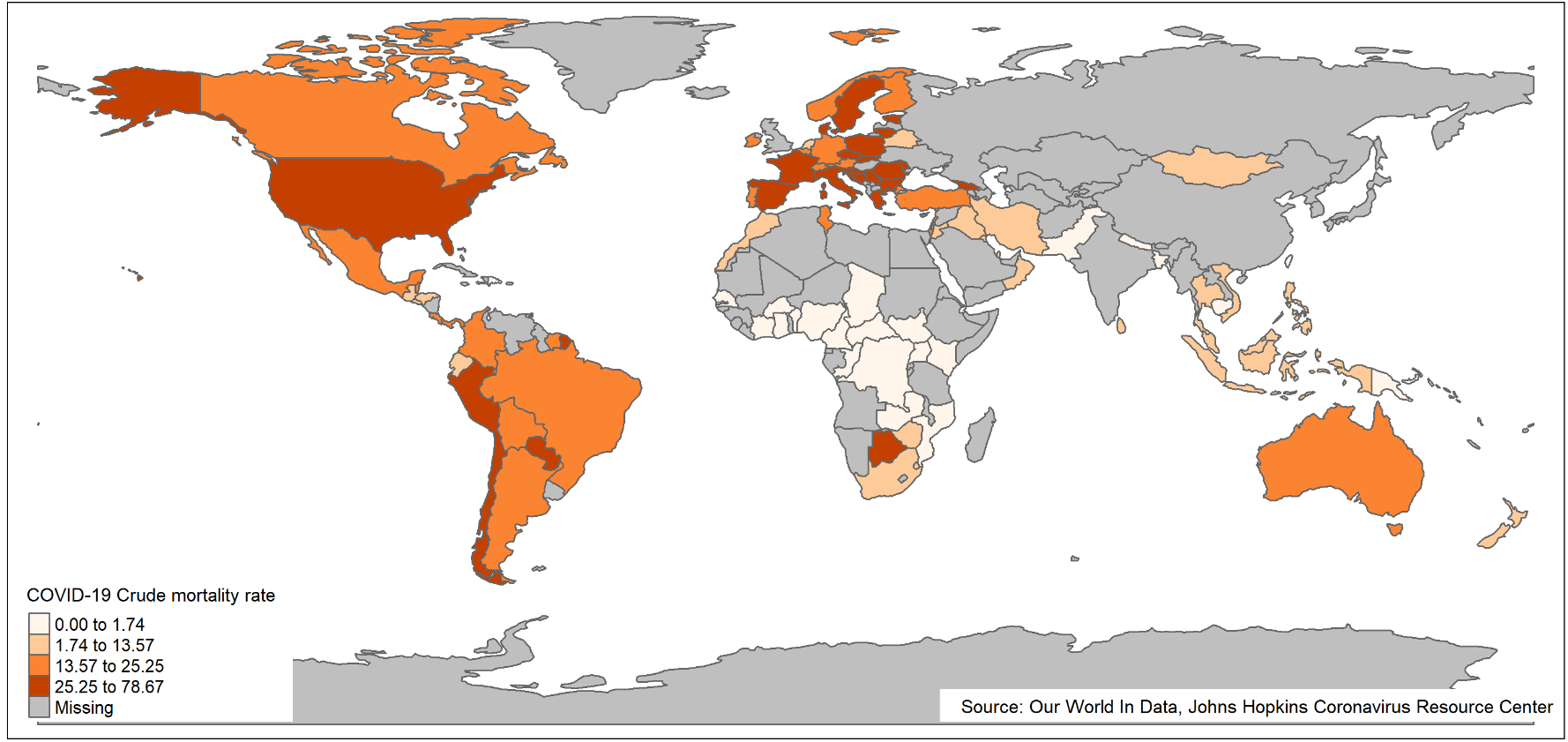
**
